# Vinylogous Nitro-Haloform Reaction Enables Aromatic Amination

**DOI:** 10.1021/acs.orglett.2c01494

**Published:** 2022-06-28

**Authors:** Claudio Monasterolo, Mauro F. A. Adamo

**Affiliations:** Centre for Synthesis and Chemical Biology, Department of Chemistry, Royal College of Surgeons in Ireland, Dublin 2, Ireland

## Abstract

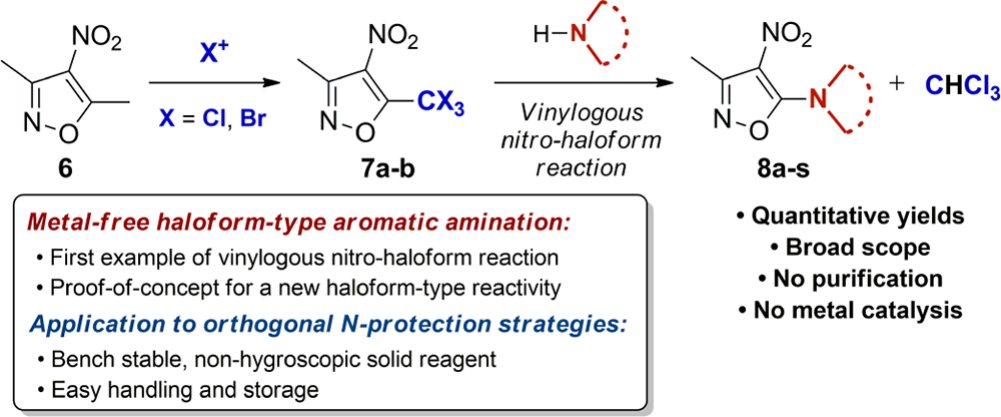

The first example of an aromatic haloform reaction is reported, defining a conceptually new haloform-type approach to the metal-free functionalization of arenes. We demonstrated that heteroarenes bearing a vinylogous nitromethane system, via the stage of a trichloromethane derivative, could undergo aromatic amination to produce N-functionalized arenes in quantitative yields and without the need for transition-metal catalysis. The haloform-type amination was implemented in the development of effective orthogonal N-protection strategies, establishing a new promising N-protecting reagent.

Aromatic amines are ubiquitous structural motifs found in both natural and synthetic compounds and are important building blocks of bioactive species and active pharmaceutical ingredient (APIs).^1^ The chemistry of aromatic amines plays a central role in modern organic synthesis due to their prevalent occurrence across a broad range of applications. Their primary industrial relevance which, through the decades, has driven an intense research effort, delivered many opportune strategies for their construction.^2^ Hence, the preparation of aromatic amines has undergone substantial advancement, from classic noncatalytic S_*N*_Ar processes^3^ to the emergence of transition-metal catalyzed methodologies.^4^ The establishment, in the late 1990s, of palladium-catalyzed cross-coupling amination^5^ marked a major breakthrough in the field and quickly became the benchmark for the preparation of aromatic amines, providing unmatched levels of efficiency and versatility in important industrial and commercial applications.^6^ In recent years, despite the leading role of transition-metal catalysis, the field has experienced a renewed interest in the delivery of new reagents and transformations,^7^ driven by the increasing demand for long-term sustainability and the concerns about the future supply of rare metal species. Although the replacement of transition-metal catalysis seems unrealistic in the near future, alternative approaches could play an important role by providing solutions for specific issues affecting metal-catalyzed processes. For instance, transition-metal species, due to their Lewis acid nature, are known to suffer from compatibility issues in the presence of strongly coordinating substrates. Specifically, small *N*-, *O*-heteroarenes, featuring reduced aromaticity and a substantial Lewis base character, still account for challenging substrates,^8^ due to their ability to engage the metal catalyst in stable coordination complexes, disrupting the catalytic cycle.^9^ The origin of such incompatibility is to be sought in the inherent chemical nature of *N*-, *O*-heteroarenes and transition metals (Lewis base/Lewis acid, respectively), which has hampered the development of a general solution so far. This suggests that switching to metal-free conditions is required in the case of heteroaromatic substrates, thus conferring primary relevance to the development of new metal-free amination methodologies. In this context, our ongoing interest in the chemistry of small *N*-, *O*-heteroarenes prompted us to investigate the development of new metal-free strategies enabling them to react with *N-*nucleophiles under mild conditions and most crucially without the need for transition-metal catalysis. 3,5-Dimethyl-4-nitroisoxazole **6**(10) (Scheme 1 and Figure 1) was chosen as a model substrate in light of the poor aromaticity and the unique ambiphilic reactivity^11^ displayed by the vinylogous nitromethane system embedded in the 4-nitroisoxazole ring.^12^

**Scheme 1:**
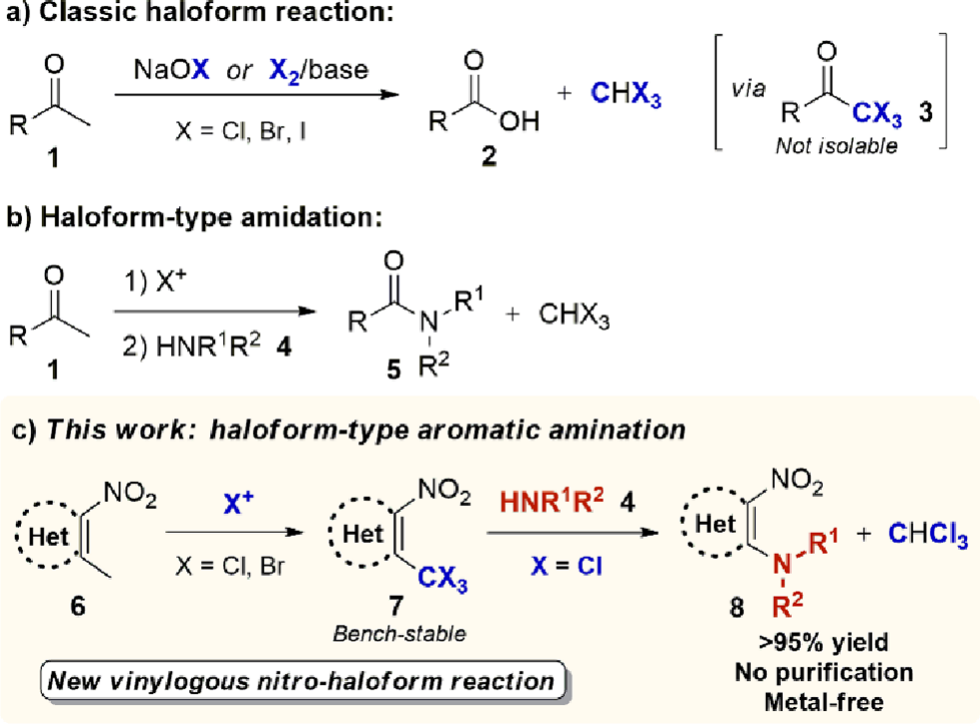
Haloform Reactivity and Applications (a) Classic haloform reaction, i.e., hydrolysis of methylketones **1** via trihalomethylketones **3**. (b) Haloform-type amidation. (c) New haloform-type metal-free aromatic amination.

**Figure 1:**
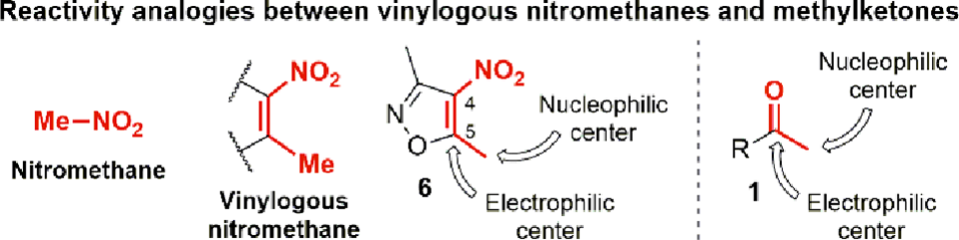
Ambiphilic reactivity of vinylogous nitromethane 3,5-dimethyl-4-nitroisoxazole **6** and reactivity analogies with methylketones **1**.

The peculiar chemical features of **6** have established it as a versatile tool in organic synthesis, finding application in a broad range of transformations^13^ and in the preparation of valuable APIs.^14^ Most significantly, our previous studies demonstrated the existence of profound analogies between the reactivity of heteroaromatic vinylogous nitromethanes, such as **6**, and carbonyl species such as methylketones **1** (Figure 1).^15^

Following from these considerations, the mechanistic rationale underpinning the design of the new amination took inspiration from the classic haloform reaction.^16^ Taking into account the established behavior of methylketones **1** under haloform conditions (Scheme 1a and b),^17^ we envisaged that **6**, through the stage of 4-nitro-5-trihalomethyl derivative **7**, could undergo regioselective amination to 5-aminoisoxazole **8**, via an unprecedented haloform-type process, i.e., vinylogous nitro-haloform reaction (Scheme 1c). We hypothesized that, in the presence of a source of halonium ions X^+^, exhaustive α-halogenation of **6** would deliver the trihalogenated derivative **7** which, in the presence of *N*-nucleophiles, would then undergo haloform-type aromatic amination to **8**, with concomitant formation of CHX_3_ (Scheme 1c). Herein we describe the development of a novel haloform-type strategy for the metal-free aromatic amination of some heteroarenes. The work outlines an innovative application of the haloform reactivity, reaching beyond the limits of the classic carbonyl-based transformation, and provides a conceptually new approach to the functionalization of aromatic compounds.

At the onset of the project, we focused on the study of the halogenation of 3,5-dimethyl-4-nitroisoxazole **6**.^18^ Compound **6** showed substantial unreactivity toward radical conditions in the presence of common chlorinating reagents, like *N*-chlorosuccinimide (NCS), 1,3-dichloro-5,5-dimethylhydantoin (DCDMH), and sulfuryl chloride (Table 1, entries 1–3). Having ruled out the radical pathway, we turned our attention to electrophilic halogenation conditions. The electrophilic chlorination of **6** with NCS required the use of a basic promoter, as no conversion was observed in the absence of a base (Table 1, entry 4). Triethylamine resulted incompatible with the reaction system, leading to degradation of the substrate (Table 1, entry 5). The structural rigidity of the N-base proved to be a critical factor for both the reactivity and stability of the substrate, as demonstrated by the caged tertiary amine 1,4-diazabicyclo[2.2.2]octane (DABCO), which improved the yield of **6** to 86% (Table 1, entry 8). A slight excess of NCS further improved the yield of **7a** to 93% while reducing the reaction time to 8 h (Table 1, entry 9).^19^ In contrast with the highly nucleophilic DABCO, the use of stronger, non-nucleophilic N-bases, such as 1,8- diazabicyclo(5.4.0)undec-7-ene (DBU) and 1,5-diazabicyclo [4.3.0]non-5-ene (DBN), resulted in extensive substrate degradation (Table 1, entries 10 and 11).

**Table 1:**
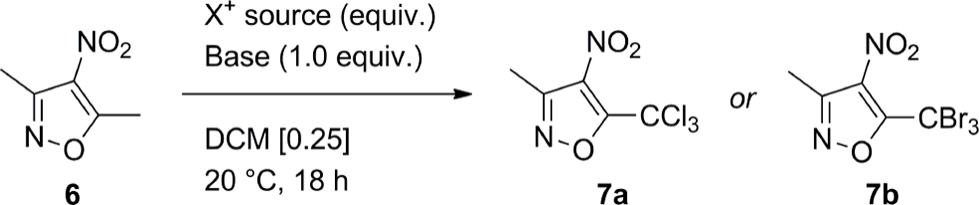
Screening of Conditions for the Electrophilic Halogenation of **6**^a^

entry	X^+^ (equiv)	base	**7a** yield (%)^b^
1^c^	NCS (3.0)	–	–d
2^c^	DCDMH (3.0)	–	–d
3^c^	SO_2_Cl_2_ (3.0)	–	–d
4	NCS (3.0)	–	– (<5)^d^
5	NCS (3.0)	Et_3_N	<5 (42)^e^
6	NCS (3.0)	DMAP	10 (29)
7	NCS (3.0)	DABCO	86 (91)
8^f^	NCS (3.5)	DABCO	93 (>95)
9^f^	NCS (1.0)	DABCO	28 (30)
10	NCS (3.5)	DBU	n.d. (>95)^g^
11	NCS (3.5)	DBN	n.d. (>95)^g^
12	NBS (3.5 + 1.0)	DABCO	84 (>95)

a The reactions were performed by stirring **6** (0.4 mmol), base (0.4 mmol), and halogenating reagent (1.2 mmol) in DCM (1.6 mL).

b Isolated yields. The values in parentheses refer to the conversion of **6**.

c Attempted radical halogenation: benzoyl peroxide (10 mol %), CCl_4_ (8 mL), 80 °C for 24 h.

d **6** recovered unreacted.

e Degradation of **6**.

f Reaction time 8 h.

g Complex mixture of unidentifiable products.

Interestingly, the chlorination showed complete regioselectivity and chemoselectivity, delivering the trichlorinated derivative **7a** as the sole product, independently from the stoichiometry of chlorinating reagent used.^20^ Finally, the bromo-analogue 3-methyl-4-nitro-5-tribromomethylisoxazole **7b** was prepared in 84% yield by using *N*-bromosuccinimide (NBS) in place of NCS (Table 1, entry 12). With substrate **7a** in hand, we proceeded to the study of the haloform-type amination to produce 5-aminoisoxazoles **8** and chloroform. Aniline **4a** was selected as a model *N*-nucleophile to carry out the initial screening of conditions (Table 2). Treating **7a** with excess **4a**, in the absence of solvent, resulted after 18 h in the generation of a new species which was identified with the desired product **8a**, together with minor amount of the side product **9a** (Table 2, entries 1 and 2), thus demonstrating the viability of our working hypothesis. To address the erosion of chemoselectivity, a comprehensive investigation of reaction conditions was undertaken. The screening of different reaction media and temperatures identified the use of tetrahydrofuran at 50 °C as the combination of choice, furnishing **8a** to 54%, together with 19% of undesired **9a** (Table 2, entry 7). Next, the effect of additional basic species was investigated. The organic base DABCO did not sensibly improve the outcome of the reaction (Table 2, entry 8). Switching to inorganic bases, potassium hydroxide proved incompatible with the substrate, leading to fast degradation of **7a** (Table 2, entry 9). On the contrary, the addition of solid potassium carbonate had a dramatic effect on the reaction, enhancing both the yield and the chemoselectivity, delivering **8a** in 89% yield while substantially suppressing the formation of **9a** (Table 2, entry 10). The reaction was further optimized by using stoichiometric amounts of both aniline and K_2_CO_3_. Under optimized conditions, **8a** was obtained in 94% yield and in pure form after a simple extractive workup (Table 2, entry 11). With optimized conditions in hand, we investigated the scope of the transformation by reacting different aromatic and aliphatic amines **4b**–**s** with **7a** to produce 5-aminoisoxazoles **8a**–**s** (Scheme 2). Excellent results were obtained in the presence of primary and secondary amines, together with a remarkable functional group compatibility. Moreover, the products were obtained in pure form after extractive workup, avoiding the need for purification. An NMR study carried out on the crude mixture (see the Supporting Information (SI) for details) identified chloroform as the sole halogenated byproduct, which suggested the participation of a polar mechanism rather than a radical pathway.^21^

**Table 2:**
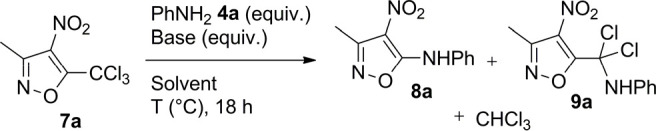
Screening of Conditions for the Haloform-type Aromatic Amination of **7a** with aniline **4a**^a^

entry	**4a**	solvent	base	*T* (°C)	**8a** yield (%)^b^
1	10.0	–	–	20	12
2	10.0	–	–	50	79 (16)^c^
3	2.5	DCM	–	20	8
4	2.5	toluene	–	20	5
5	2.5	THF	–	20	11
6	2.5	toluene	–	50	15
7	2.5	THF	–	50	54 (19)^c^
8^d^	2.5	THF	DABCO	50	45
9^d^	2.5	THF	KOH	50	n.d.^e^
10^d^	2.5	THF	K_2_CO_3_	50	89 (<5)^c^
11^f^	1.1	THF	K_2_CO_3_	50	94^c^

a Reaction conditions: **2** (0.25 mmol), solvent (0.25 mL), sealed tube.

b Yields determined by ^1^H NMR analysis of the crude reaction mixture, unless otherwise stated.

c Isolated yields. The values in brackets refer to the yield of **9a**.

d Base (0.50 mmol, 2.0 equiv).

e Fast degradation of **7a**.

f K_2_CO_3_ (0.25 mmol, 1.0 equiv)

**Scheme 2:**
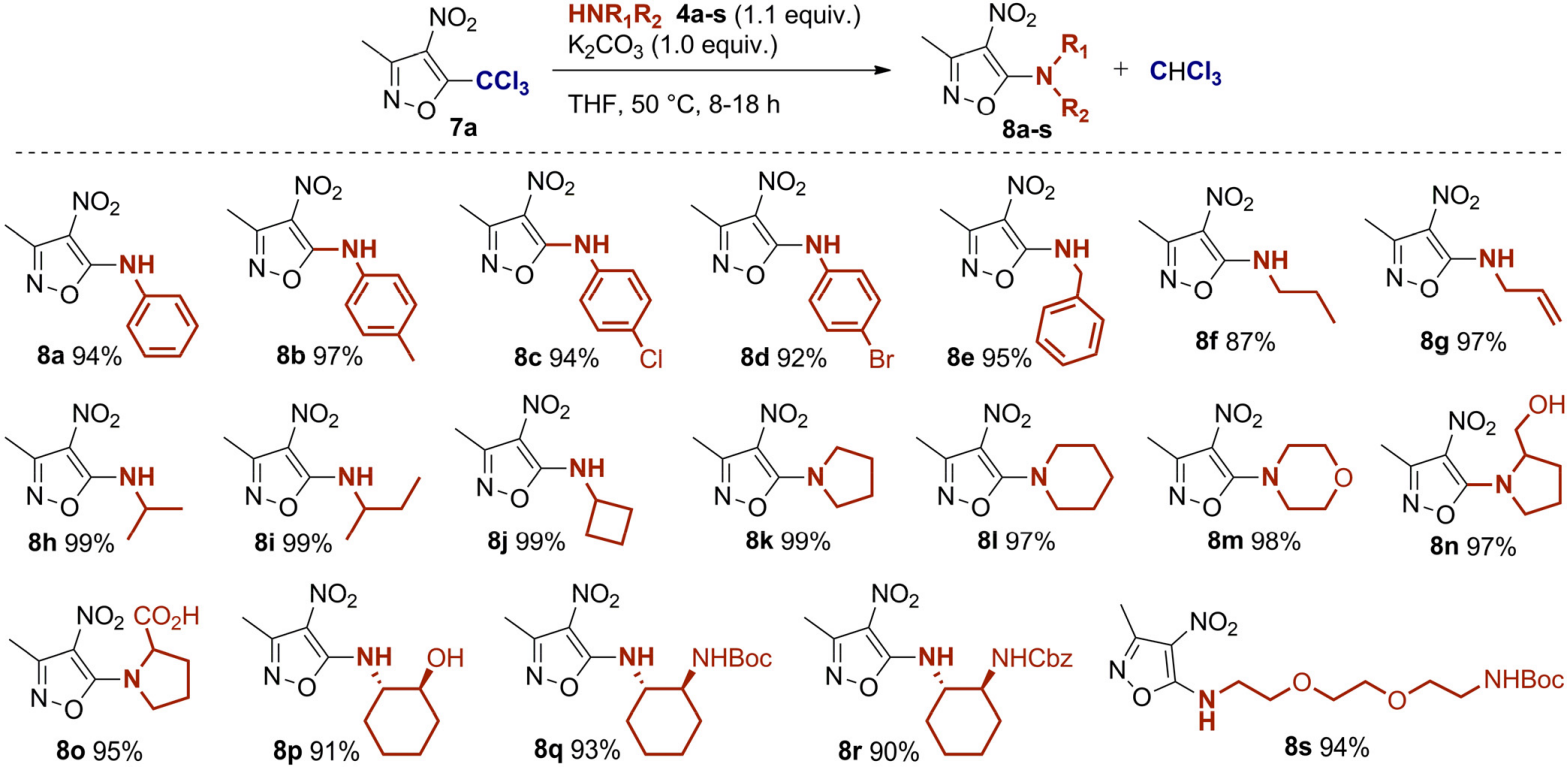
Scope of the Metal-Free Haloform-type Aromatic Amination of **7a** with Amines 4a–**s** Reaction conditions: **7a** (0.4 mmol), amine **4a**–**s** (0.44 mmol), K_2_CO_3_ (0.4 mmol) in THF (0.5 mL) at 50 °C, sealed tube, see ESI for detailed procedures. Isolated yields of analytically pure products **8a**–**s** after extractive workup.

The excellent yields and operational simplicity of the methodology prompted us to explore its use as novel a N-protection strategy for amines (Scheme 3). We reasoned that the unique reactivity of **7a**, together with the mild conditions and selectivity of the transformation, constituted an ideal set of features for its use as a N-protecting reagent. Primary and secondary amines could be efficiently protected, in the form of *N*-isoxazolyl amines **8**, via haloform-type amination with **7a**. The subsequent deprotection step could exploit the known ability of 4-nitroisoxazoles to undergo ring-opening to carboxylates, known as the Sarti Fantoni reaction.^22^ Deprotection of **8** to free amine **4** would entail a novel cascade pathway involving tandem ring-opening/decarboxylation, via the stage of carbamate intermediate **11** (Scheme 3, a).

**Scheme 3:**
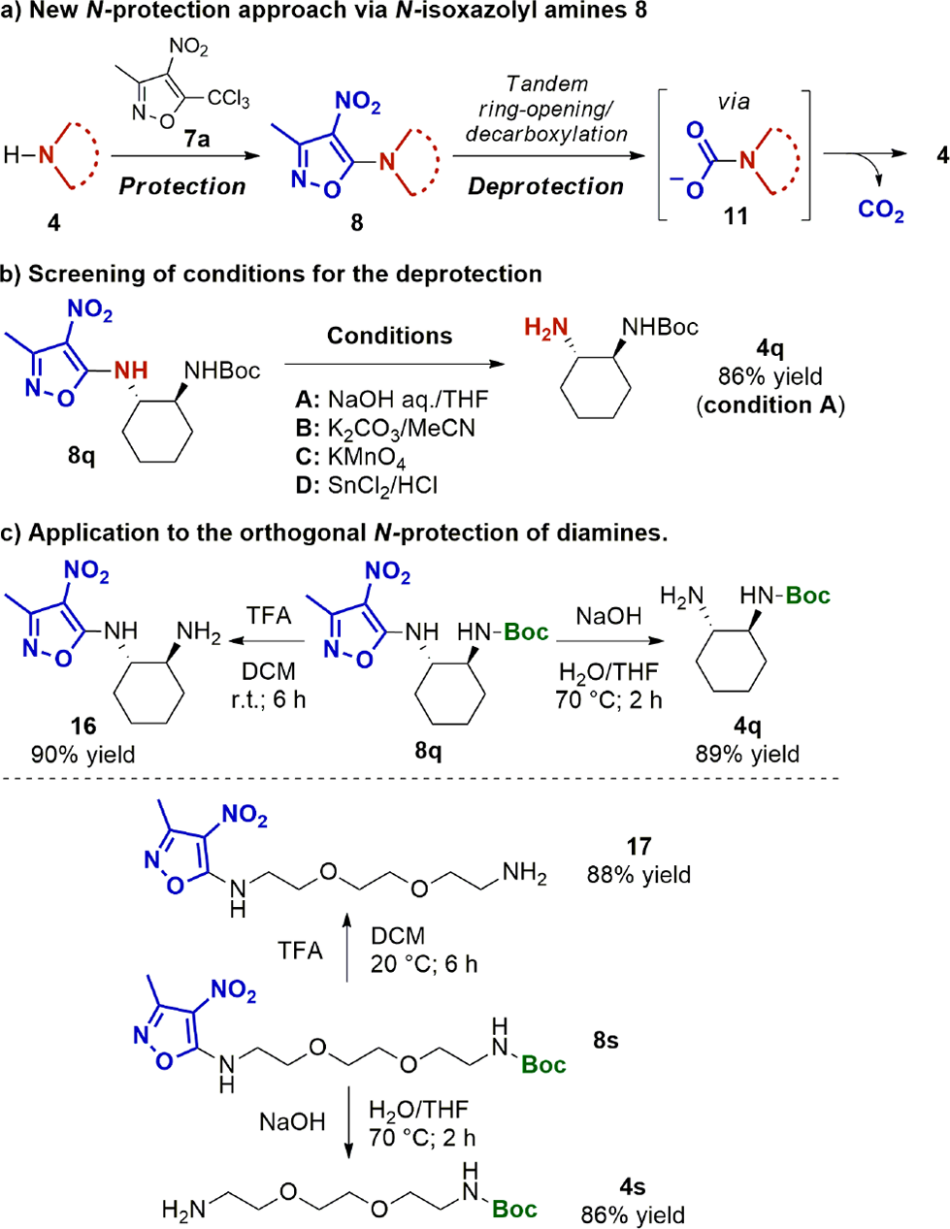
Application to the Protection of N-Substrates

To demonstrate the feasibility of the proposed strategy, we first focused on the search of suitable deprotection conditions, using *N*-Boc,*N*^*1*^-isoxazolyl diamine **8q** as the model substrate (Scheme 3b). Selective deprotection of **8q** to **4q** took place smoothly by treatment with aqueous NaOH in THF, thus indicating the base-labile nature of the *N*-isoxazolyl protecting group. The nature of the basic system was critical to the reaction, as replacing NaOH/THF with K_2_CO_3_/MeCN proved ineffective. In addition, the *N-*isoxazolyl functionality showed remarkable stability toward acids as well as compatibility with both oxidative and reductive conditions; see the SI for details on the reaction conditions screened. On the basis of these findings, we finally applied **7a** to the development of new effective orthogonal N-protection strategies for diamines in combination with acid-labile *N*-Boc functionalities (Scheme 3c).^23^

In conclusion, we have developed a conceptually new haloform-type approach to the metal-free aromatic amination of some type of heteroarenes. The aromatic haloform-type reaction reported herein enables the straightforward preparation of functionalized amino arenes under mild conditions and avoiding the use of transition-metal catalysis. We demonstrated that 3-methyl-4-nitro-5-trichloromethylisoxazole **7a**, easily prepared via electrophilic chlorination of the parent compound **6**, in the presence of primary and secondary amines underwent aromatic amination to produce 5-aminoisoxazoles **8** in quantitative yields and without the need for purification. The metal-free amination proceeded via an unprecedented vinylogous haloform mechanism, involving the 1,4-conjugate addition of *N*-nucleophiles to a vinylogous nitro-trichloromethane system, followed by chloroform elimination, and was therefore accordingly named *vinylogous nitro-haloform reaction*. The study represents the first example of aromatic haloform-type process, for the first time extending the classic haloform reactivity to an unprecedented class of aromatic substrates. The haloform-type amination provided a new approach to the N-protection of primary and secondary amines and to the orthogonal protection of diamines, demonstrating the synthetic utility of the method. Building on the preliminary findings reported herein, further studies are underway to widen the scope to structurally diverse substrates as well as different classes of aromatic transformations.

## Supporting Information Available

Experimental procedures, characterization data and NMR spectra for all compounds (PDF)
