# Supplementary material for: Vinylogous Nitro-Haloform Reaction Enables Aromatic Amination
Source: Org Lett. 2022 Jun 28;24(26):4729–33. doi: 10.1021/acs.orglett.2c01494 (PMC9274776; doi:10.1021/acs.orglett.2c01494)
Supplement: Supplementary file 1 — ol2c01494_si_001.pdf [file ol2c01494_si_001.pdf]

Vinylogous Nitro-Haloform Reaction Enables Aromatic Amination

Supporting Information

Claudio Monasterolo*† and Mauro F. A. Adamo*

*† Dr C. Monasterolo – *Centre for Synthesis and Chemical Biology, School of Chemistry, University College Dublin, Belfield, Dublin 4, Ireland*
E-mail: claudio.monasterolo@ucd.ie

* Prof. M. F. A. Adamo – *Centre for Synthesis and Chemical Biology, Department of Chemistry, Royal College of Surgeons in Ireland, 123 St Stephen's Green, Dublin 2, Ireland*
E-mail: madamo@rcsi.ie

Table of contents

Materials and method S-2
General information S-2
Supporting experimental data S-3
Chemoselectivity study for the electrophilic halogenation of 3,5-dimethyl-4-nitroisoxazole **6** S-3
NMR study of the haloform-type amination: polar vs radical mechanism S-4
Vinylogous nitro-haloform reaction: proposed mechanism S-6
Screening of conditions for the deprotection of *N*-isoxazolyl protecting group via tandem ring-opening/decarboxylation S-7
Experimental procedures and characterizations S-8
Base-promoted electrophilic halogenation of **6**: S-8
Preparation of 3-methyl-4-nitro-5-trichloromethylisoxazole **7a** S-8
Preparation of 3-methyl-4-nitro-5-tribromomethylisoxazole **7b** S-9
Haloform-type metal-free aromatic amination of **7a** S-10
Application of **7a** as N-protecting reagent for the orthogonal N-protection of diamines S-17
Base-promoted orthogonal deprotection of **8q** to **4q** S-17
Acid-promoted orthogonal deprotection of **8q** to **16** S-18
Orthogonal N-deprotection of N-Boc,N1-isoxazolyl diamine **8s** S-19
Synthetic example at 5 mmol scale S-20
NMR Spectra S-21
References S-47

Materials and methods

General information

NMR spectra were recorded on a Bruker 400 spectrometer and on a Varian VNMRS 400 spectrometer at 25 °C. Assignments are based on standard ^{1}H-^{1}H and ^{1}H-^{13}C two-dimensional techniques. Chemical shifts (δ) are reported in ppm relative to residual solvent signals for ^{1}H-NMR and ^{13}C-NMR ($CDCl_3$: ^{1}H-NMR δ = 7.26 ppm and ^{13}C NMR δ = 77.16 ppm; C_6D_6: ^{1}H-NMR δ = 7.16 ppm and ^{13}C NMR δ = 128.06 ppm). Coupling constants (*J*) are in Hz. Multiplicities are reported as follow: s = singlet, d = doublet, dd = doublet of doublets, t = triplet, q = quartet, m = multiplet and br = broad. Mass spectra were recorded on a Micromass LCT spectrometer using electrospray ionisation (ESI) techniques. Commercially available reagents were purchased from Sigma-Aldrich, Fluorochem Ltd. and Acros Organics. Commercial reagents and analytical grade solvents were used as supplied, unless otherwise stated. *N*-Chlorosuccinimide was recrystallized from hot glacial acetic acid.[1] *N*-Bromosuccinimide was recrystallized from hot water.[2] Dichloromethane and chloroform were dried over 4Å molecular sieves and stored in J Young flasks over 4Å molecular sieves under N_2. Dry THF was obtained from an Inert Pure Solv Micro solvent purification system and stored in J Young flasks over 4Å molecular sieves under N_2. Oxygen-free nitrogen was obtained from BOC gases and passed over activated 4Å molecular sieves. Reactions were monitored by TLC analysis performed on Merck pre-coated Kieselgel 60 F_{254} TLC aluminium plates, visualized via UV, $KMnO_4$ solution or ninhydrin solution. Flash column chromatography was performed using Davisil silica with particle size 40-63 µm or neutral alumina (activated, neutral, Brockmann activity I).

Supporting experimental data

Chemoselectivity study for the base-promoted electrophilic halogenation of 3,5-dimethyl-4-nitro isoxazole 6

Table 1. Chemoselectivity of the base-promoted electrophilic halogenation of **6** with NCS and NBS.

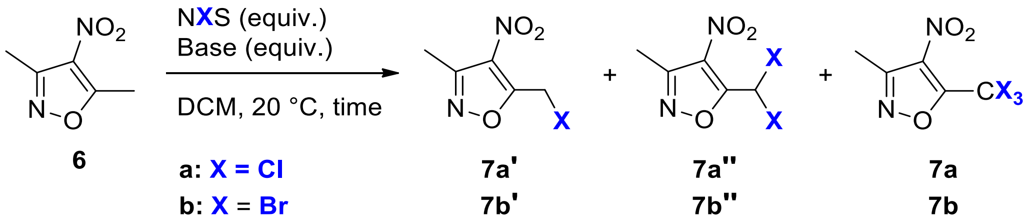

Entry	NXS (equiv.)	Base (equiv.)	Time (h)	6 Conversion (%)[a]	7a'/7a''/7a (%)[b]	7b'/7b''/7b (%)[b]
**1**	NCS (3.0)	DABCO (1.0)	18	91	0 : 0 : 86	-
**2**	NCS (3.5)	DABCO (1.0)	8	>95	0 : 0 : 93	-
**3**	NCS (1.0)	DABCO (1.0)	18	30	0 : 0 : 28	-
**4**	NCS (1.0)	K_2CO_3 (1.0)	18	30	0 : 0 : 27	-
**5**	NBS (3.5)	DABCO (1.0)	18	>95	-	0 : 19 : 72
**6**[c]	NBS (3.5 + 1.0)	DABCO (1.0)	24	>95	-	0 : 10 : 84
**7**[d]	NCS (3.0)	Et_3N (1.0)	18	38	0 : 0 : <5	-

[a] Conversion calculated via ^{1}H-NMR analysis of the crude reaction mixture. [b] Isolated yields. [c] After 18 hours additional 1.0 equivalent of NBS was added. [d] Substrate degradation was observed in the presence of Et_3N.

Chemoselectivity of the electrophilic chlorination

The base-promoted electrophilic chlorination of **6** with NCS showed complete chemoselectivity, producing exclusively the trichlorinated product **7a**, even in the presence of sub-stoichiometric amounts of NCS (Table 1, entries 3 and 4). The monochlorinated **7a'** and the dichlorinated derivative **7a''** were not observed in the reaction mixture, even after short reaction times. These observations suggested a progressive enhancement of the **6** α-position reactivity resulting from the introduction of the first and second α-chlorine substituents, which resulted in the exhaustive chlorination to **7a**.

Chemoselectivity of the electrophilic bromination

On the other hand, the electrophilic bromination of **6** under previously optimized conditions, showed reduced chemoselectivity, resulting in reduced yields of the desired product **7b** (72% yield), due to the presence of 19% of the dibrominated derivative **7b''** (Table 1, entry 5). The yield of **7b** could be increased to 84% with additional 1.0 equivalent of NBS added after 18 hours (Table 1, entry 6). The lower chemoselectivity of the bromination was likely due to the steric hindrance of the bromine atoms compared to chlorine, which countered the activation of the α-position towards electrophilic halogenation resulting from the electronic effect of the α-halogen substituents.

NMR study of the haloform-type amination: polar vs radical mechanism

The mechanism of the haloform-type amination was investigated by NMR analysis of the crude reaction mixture, focusing on the identification of the halogenated by-products generated over the course of the reaction, in order to differentiate between the two possible reaction pathways: electrophilic (polar) and radical. The study identified chloroform as the sole halogenated species present in the reaction mixture at different reaction times. Considering the volatile nature of the chlorinated species potentially produced in the transformation, an ad-hoc set-up of the reaction and optimized reaction conditions were developed. To minimize the loss of volatiles and ensure the accuracy of the analyses, the reaction was run in a glass sealed tube using benzene-d_6 as both reaction solvent (replacing THF) and NMR solvent.

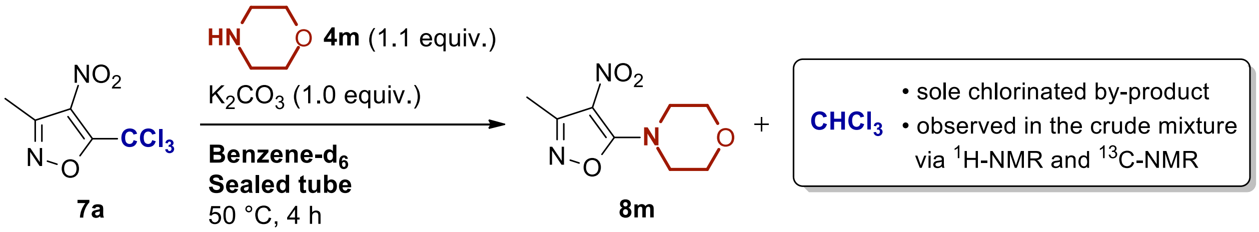

Scheme 1. NMR study for the identification of the chlorinated by-products generated over the course of the haloform-type amination.

Procedure: In a 10 mL glass sealed tube, **7a** (73.7 mg, 0.3 mmol) was dissolved in benzene-d_6 (0.6 mL). To the solution was added potassium carbonate (41.5 mg, 0.3 mmol) followed by morpholine **4m** (29.0 uL, 0.33 mmol). The tube was sealed and placed in an oil bath at 50 °C. The mixture was stirred at 50 °C for 4 hours. The sealed tube was removed from the oil bath and cooled to room temperature, the stirring discontinued and the mixture decanted. Using a 500 uL gas-tight glass syringe, an aliquot of the crude mixture (250 uL) was transferred directly into a gas-tight NMR tube, followed by benzene-d_6 (0.4 mL), and the tube was sealed. The ^{1}H-NMR and ^{13}C-NMR analyses indicated $CHCl_3$ as the only chlorinated species in the reaction mixture ($CHCl_3$ in benzene-d_6: ^{1}H-NMR δ = 6.23 ppm, ^{13}C-NMR δ = 77.72 ppm). The other species were identified as the 5-aminoisoxazole product **8m**, unreacted substrate **7a** and amine **4m** (Figure 1).

Figure 1. NMR analyses in benzene-d_6 of the crude reaction mixture, showing the formation of $CHCl_3$.

^{1}H-NMR crude reaction mixture (C_6D_6)

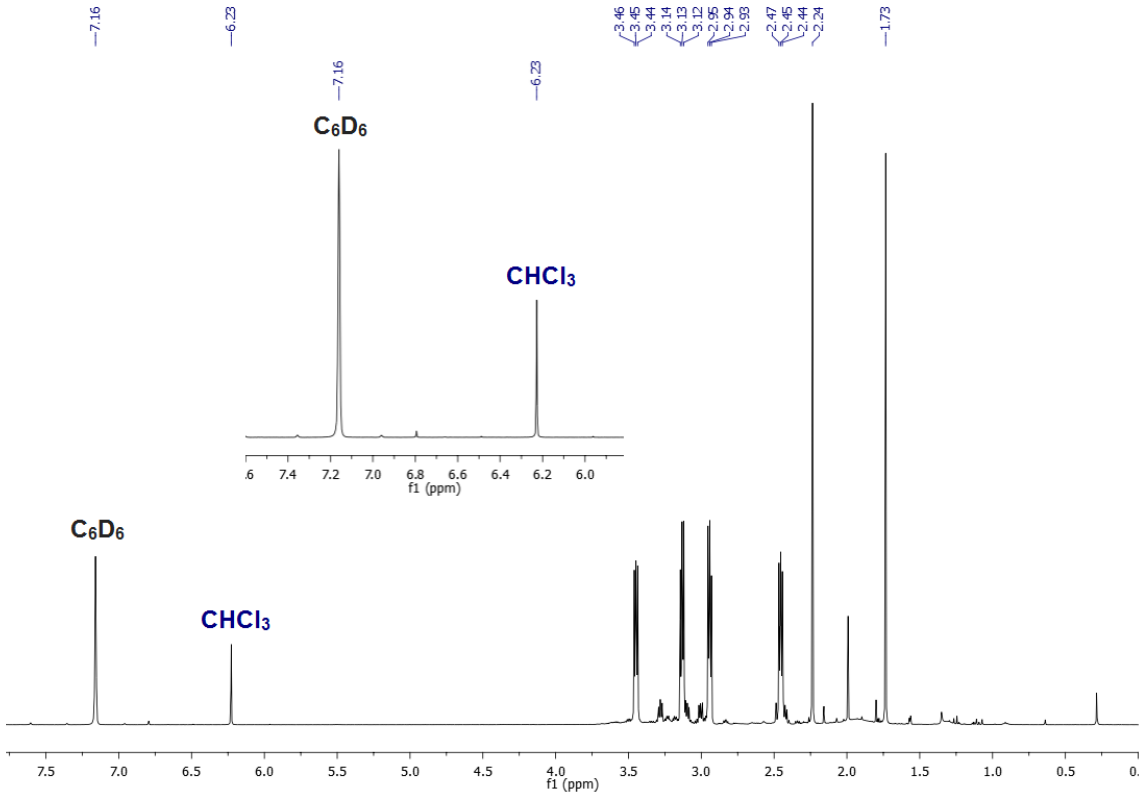

^{13}C-NMR crude reaction mixture (C_6D_6)

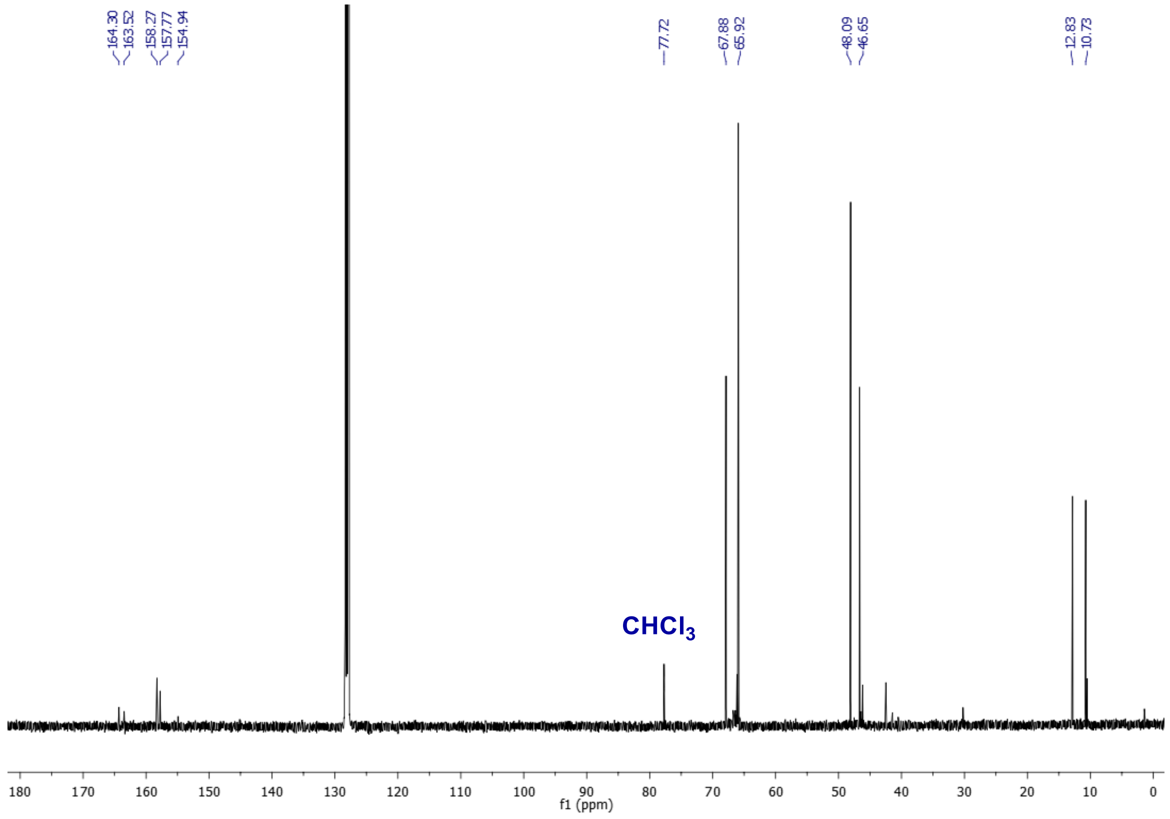

Vinylogous nitro-haloform reaction: proposed mechanism

The newly developed metal-free aromatic amination can be described as a vinylogous nitro-haloform reaction. The process entails the base-promoted nucleophilic aromatic substitution (S_NAr) of **7a** with primary and secondary amines **4**, and involves a two-step addition/elimination mechanism, via the stage of Meisenheimer complex intermediate (Scheme 2).[3] Vinylogous 1,4-addition of the amine **4** to the C-5 of **7a** produces the Meisenheimer complex **19**, which undergoes deprotonation by K_2CO_3 to intermediate species **20**. Elimination of CCl_3^- anion delivers the 5-aminoisoxazole products **8**, together with $CHCl_3$, restoring the aromaticity of the system. It is important to note that, in line with recent reports which redefined important mechanistic aspects behind nucleophilic aromatic substitution reactions, an alternative concerted substitution pathway (cS_NAr) could be taking place in place of the S_NAr two-stage addition/elimination process described above.[4] These studies demonstrated that the concerted cS_NAr pathway intervenes in a range of nucleophilic aromatic substitution reactions, in place of the widely accepted two-stage mechanism, thus making it necessary to reconsider the actual relevance of the standard addition/elimination mechanism in S_NAr processes. As a result, despite the plausibility of the mechanism proposed in Scheme 2 for the new haloform-type amination reaction, further studies are required to establish the actual pathway involved in the novel heteroaromatic amination process.

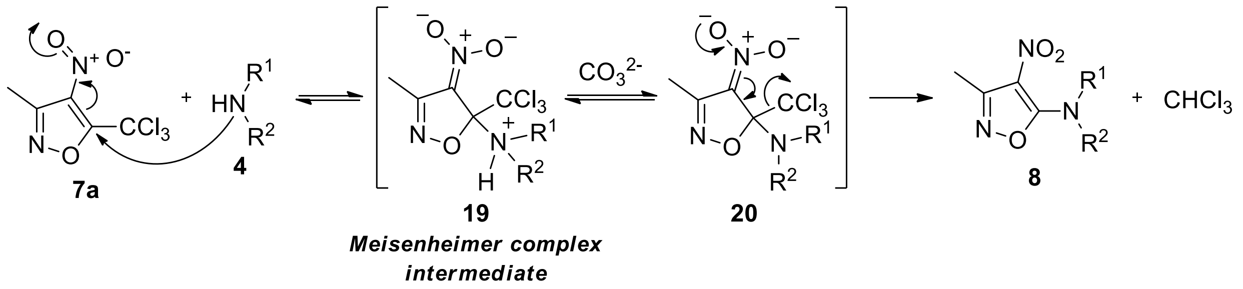

Scheme 2. Proposed mechanism for the vinylogous nitro-haloform reaction.

Screening of conditions for the deprotection of *N*-isoxazolyl protecting group via tandem ring-opening/decarboxylation

The deprotection of the *N*-isoxazolyl protecting group was studied by screening previously reported conditions for the ring-opening of 4-nitroisoxazoles: basic (A and B); oxidative (C) and reductive/acidic conditions (D), using **8q** as a model substrate of a polyfunctionalized amine (Scheme 3).

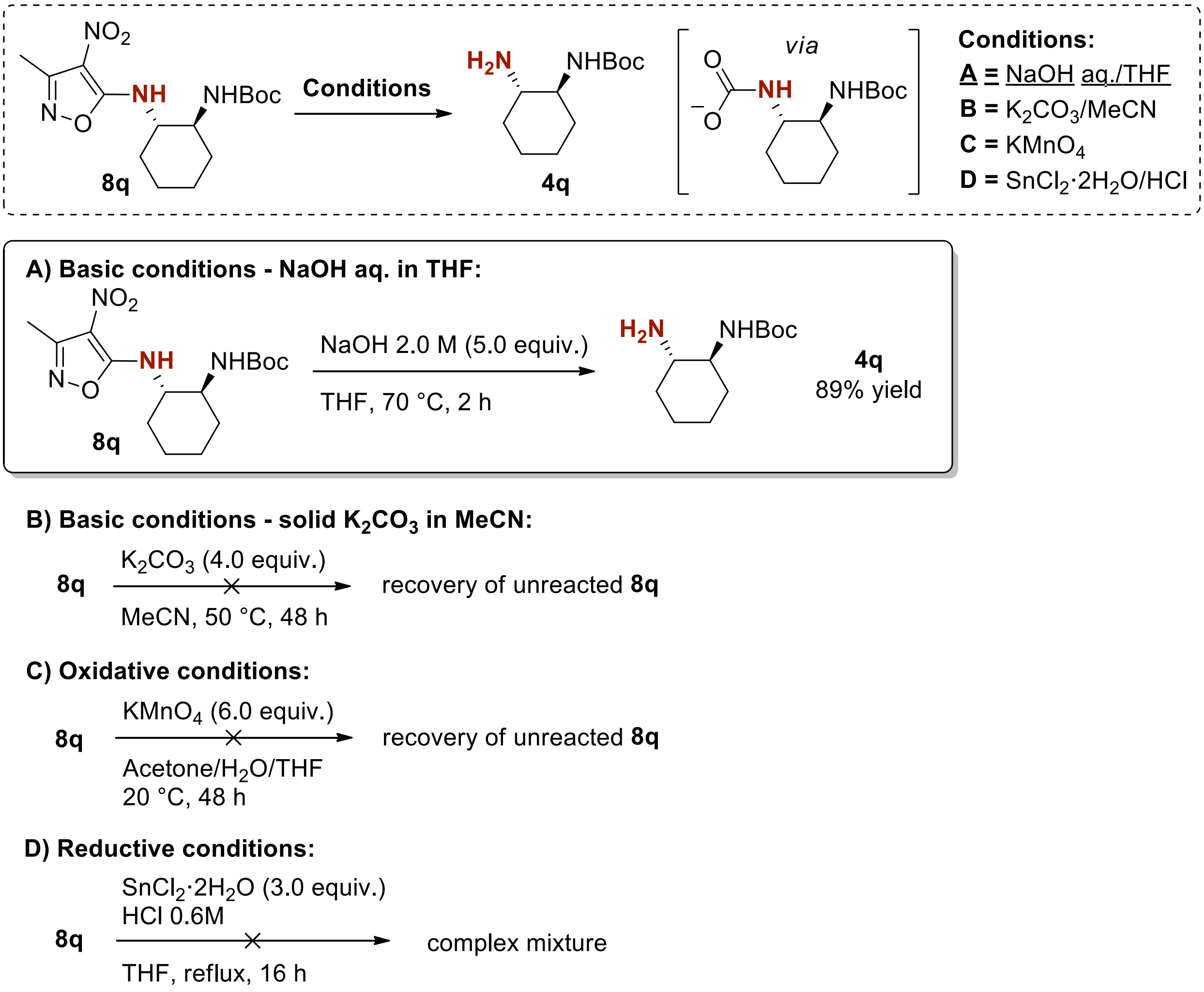

Scheme 3. Screening of deprotection conditions for *N*-isoxazolyl protected amine **8q**.

The use of NaOH 2.0 M in THF at 70 °C for 2 hours (Conditions A) proved effective in the deprotection of **8q** via tandem ring-opening/decarboxylation, producing **4q** in 89% yield. On the contrary, the alternative basic system K_2CO_3 in MeCN was ineffective, returning unreacted **8q** (Conditions B). Oxidative and reductive conditions were also ineffective, the former resulting in recovery of unreacted **8q**, while the latter producing a complex mixture of products (Scheme 3).

Experimental procedures and characterizations

Base-promoted electrophilic halogenation of 6

Preparation of 3-methyl-4-nitro-5-trichloromethylisoxazole, 7a

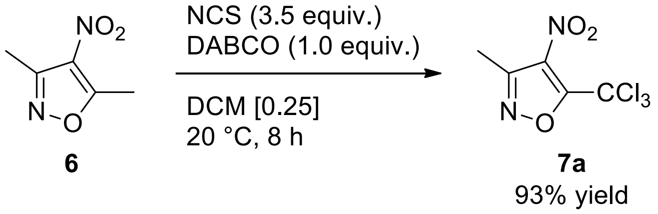

To a stirred solution of 3,5-dimethyl-4-nitroisoxazole **6** (2.0 g, 14.08 mmol) in DCM (40 mL) was added 1,4-diazabicyclo[2.2.2]octane (DABCO) (1.58 g, 14.08 mmol) at room temperature. The solution was cooled to 0 °C with an ice bath and *N*-chlorosuccinimide (NCS) (6.58 g, 49.28 mmol, **Note 1**) was added portionwise over 5-10 minutes. The ice bath was removed and the reaction was stirred at 20 °C for 8 hours, monitoring the progression of the reaction by TLC (pentane/Et_2O 8:2). The mixture was then filtered on Celite, washing with DCM. The filtrate was washed with saturated NH_4Cl (50 mL), water (50 mL) and brine (50 mL), the organic phase dried over sodium sulfate, filtered and the solvent removed under reduced pressure. The crude product was purified by column chromatography on silica gel eluting with pentane/Et_2O 98:2 to obtain **7a** as a white crystalline solid (93% yield).

Note 1: Recently recrystallized NCS was used. However, similar results could be obtained also with lower purity NCS. In this case, to ensure the completion of the reaction, an additional amount of NCS (0.1 - 0.2 equiv.) was added after 8 hours.

3-Methyl-4-nitro-5-trichloromethylisoxazole, 7a

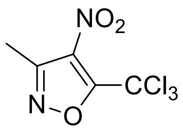

White solid, 3.21 g, 93% yield, R_f = 0.58 pentane/Et_2O 8:2.

^{1}H-NMR (400 MHz, $CDCl_3$): δ 2.60 (s, 3H).

^{13}C-NMR (101 MHz, $CDCl_3$): δ 164.1, 158.1, 128.1, 83.8, 11.7.

HRMS (ESI): calculated for $C_5H_3N_2O_3Cl_3$ ($[M]^+$) 243.9209; found: 243.9216.

Preparation of 3-methyl-4-nitro-5-tribromomethylisoxazole, 7b

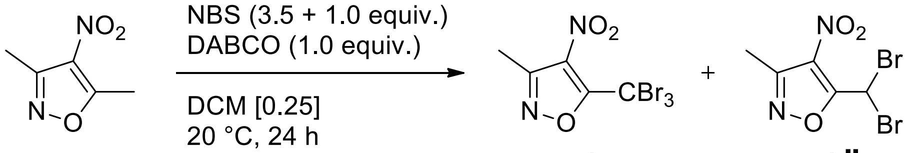

6 **7b** 84% yield **7b''** 10% yield

To a stirred solution of 3,5-dimethyl-4-nitroisoxazole **6** (1.0 g, 7.04 mmol) in DCM (20 mL) was added 1,4-diazabicyclo[2.2.2]octane (DABCO) (790 mg, 7.04 mmol) at room temperature. The solution was cooled to 0 °C with an ice bath and *N*-bromosuccinimide (NBS) (4.39 g, 24.64 mmol) was added portionwise over 5 minutes. The ice bath was removed and the reaction was stirred at 20 °C for 18 hours. After 18 hours NBS (1.25 g, 7.04 mmol) was added, and the reaction stirred at 20 °C for additional 6-8 hours, monitoring the progression by TLC (pentane/Et_2O 8:2). The mixture was then filtered on Celite, washing with DCM (50 mL). The filtrate was washed with saturated NH_4Cl (30 mL), water (30 mL) and brine (30 mL), the organic phase dried over sodium sulfate, filtered and the solvent removed under reduced pressure. The crude product was purified by column chromatography on silica gel eluting with pentane/Et_2O 99:1 to obtain **7b** (84% yield).

3-Methyl-4-nitro-5-tribromomethylisoxazole, 7b

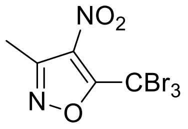

White solid, 2.24 g, 84% yield, R_f = 0.51 (pentane/Et_2O 8:2).

^{1}H-NMR (400 MHz, $CDCl_3$): δ 2.58 (s, 3H).

^{13}C-NMR (101 MHz, $CDCl_3$): δ 165.2, 158.2, 127.1, 110.1, 12.0.

HRMS (ESI): calculated for $C_5H_4N_2O_3Br_3$ ($[M+H]^+$) 376.7767; found: 376.7775.

3-Methyl-4-nitro-5-dibromomethylisoxazole, 7b''

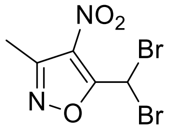

Light yellow solid, 0.21 g, 10% yield, R_f = 0.47 (pentane/Et_2O 8:2).

^{1}H-NMR (400 MHz, $CDCl_3$): δ 7.20 (s, 1H), 2.60 (s, 3H).

^{13}C-NMR (101 MHz, $CDCl_3$): δ 167.0, 156.1, 110.2, 20.8, 11.8.

HRMS (ESI): calculated for $C_5H_4N_2O_3Br_2$ ($[M]^+$) 297.8589; found: 297.8600.

Haloform-type metal-free aromatic amination of 7a

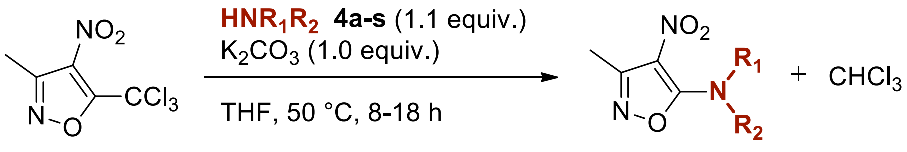

General procedure for the haloform-type amination:

3-methyl-4-nitro-5-trichloromethylisoxazole **7a** (100.0 mg, 0.407 mmol) was dissolved in THF (0.5 mL) in a 10 mL test tube at room temperature, avoiding any precautions to exclude moisture and air. Potassium carbonate (56 mg, 0.407 mmol) was added to the solution followed by the amine **4a-s** (0.448 mmol, 1.1 equiv.). The reaction tube was sealed with a rubber septum, heated to 50 °C and stirred at this temperature for the indicated time. The reaction mixture was partitioned between H_2O (10 mL) and DCM (10 mL) and the aqueous phase extracted with DCM (3 x 10 mL). The combined organic phases were dried over sodium sulfate, filtered and the solvent removed under reduced pressure to obtain pure products **8a-s**. The products were obtained in analytically pure form after the extractive work-up, without the need for column chromatography or further purification.

(3-Methyl-4-nitro-isoxazol-5-yl)-phenylamine, 8a

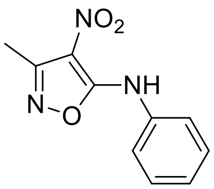

Yellow solid, 84 mg, 94% yield. Reaction time = 18 hours. R_f = 0.63 (pentane/EtOAc 6:4).

^{1}H-NMR (400 MHz, $CDCl_3$): δ 9.47 (br s, 1H), 7.49 – 7.42 (m, 4H), 7.28 – 7.24 (m, 1H), 2.54 (s, 3H).

^{13}C-NMR (101 MHz, $CDCl_3$): δ 162.5, 156.0, 135.2, 129.9, 126.2, 120.1, 112.0, 12.0.

HRMS (ESI): calculated for $C_{10}H_9N_3O_3$ ($[M]^+$) 219.0644; found 219.0652.

(3-Methyl-4-nitro-isoxazol-5-yl)-*para*-tolylamine, 8b

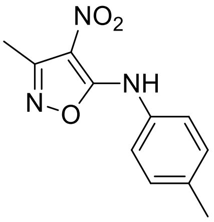

Brown solid, 92 mg, 97% yield. Reaction time = 18 hours. R_f = 0.60 (pentane/EtOAc 6:4).

^{1}H-NMR (400 MHz, $CDCl_3$): δ 9.43 (br s, 1H), 7.35 (d, *J* = 8.4 Hz, 2H), 7.23 (d, *J* = 8.4 Hz, 2H), 2.53 (s, 3H), 2.36, (s, 3H).

^{13}C-NMR (101 MHz, $CDCl_3$): δ 162.6, 156.0, 136.3, 132.6, 130.4, 120.2, 111.9, 21.1, 12.0.

HRMS (ESI): calculated for $C_{11}H_{11}N_3O_3$ ($[M]^+$) 233.0800; found 233.0798.

(4-Chlorophenyl)-(3-methyl-4-nitro-isoxazol-5-yl)-amine, 8c

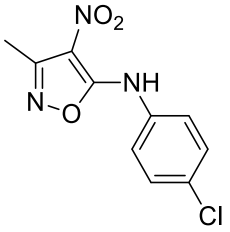

Yellow solid, 97 mg, 94% yield. Reaction time = 18 hours. R_f = 0.58 (pentane/EtOAc 6:4).

^{1}H NMR (400 MHz, $CDCl_3$): δ 10.81 (br s, 1H), 7.58 – 7.56 (m, 2H), 7.51 – 7.49 (m, 2H), 2.42 (s, 3H).

^{13}C-NMR (101 MHz, $CDCl_3$): δ 162.4, 155.8, 134.9, 130.2, 129.1, 124.5, 111.6, 11.7.

HRMS (ESI): calculated for $C_{10}H_8ClN_3O_3$ ($[M]^+$) 253.0254; found 253.0251.

(4-Bromophenyl)-(3-methyl-4-nitro-isoxazol-5-yl)-amine, 8d

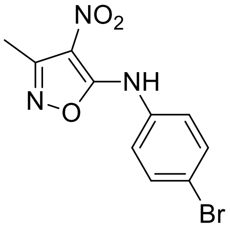

Yellow solid, 111 mg, 92% yield. Reaction time = 18 hours. R_f = 0.60 (pentane/EtOAc 6:4).

^{1}H NMR (400 MHz, $CDCl_3$): δ 10.79 (br s, 1H), 7.64 – 7.62 (m, 2H), 7.52 – 7.50 (m, 2H), 2.42 (s, 3H).

^{13}C-NMR (101 MHz, $CDCl_3$): δ 162.4, 155.8, 135.3, 132.0, 124.7, 118.4, 111.6, 11.7.

HRMS (ESI): calculated for $C_{10}H_8BrN_3O_3$ ($[M]^+$) 296.9749; found 296.9741.

(3-Methyl-4-nitro-isoxazol-5-yl)-benzylamine, 8e

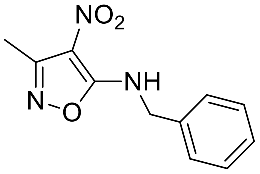

Orange solid, 90 mg, 95% yield. Reaction time = 16 hours. R_f = 0.26 (pentane/EtOAc 7:3).

^{1}H NMR (400 MHz, $CDCl_3$): δ 7.80 (br s, 1H), 7.40 – 7.34 (m, 5H), 4.70 (d, J = 6.4 Hz, 2H), 2.48 (s, 3H).

^{13}**C-NMR** (101 MHz, $CDCl_3$): δ 165.2, 156.5, 135.5, 129.3, 128.7, 128.0, 111.1, 46.8, 12.0.

HRMS (ESI): calculated for $C_{11}H_{11}N_3O_3$ ($[M]^+$) 233.0800; found 233.0798.

(3-Methyl-4-nitro-isoxazol-5-yl)-propylamine, 8f

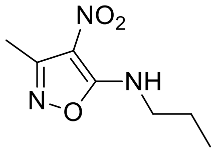

Yellow solid, 65 mg, 87% yield. Reaction time = 12 hours. R_f = 0.60 (pentane/EtOAc 6:4).

^{1}**H NMR** (400 MHz, $CDCl_3$): δ 7.56 (br s, 1H), 3.50 (q, *J* = 6.8 Hz, 2H), 2.46 (s, 3H), 1.78 – 1.69 (m, 2H), 1.01 (t, *J* = 7.4 Hz, 3H).

^{13}**C-NMR** (101 MHz, $CDCl_3$): δ 165.5, 156.3, 110.9, 44.6, 23.0, 12.0, 11.2.

HRMS (ESI): calculated for $C_7H_{11}N_3O_3$ ($[M]^+$) 185.0800; found 185.0795.

(3-Methyl-4-nitro-isoxazol-5-yl)-allylamine, 8g

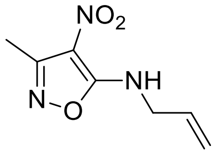

Brown solid, 72 mg, 97% yield. Reaction time = 12 hours. R_f = 0.61 (pentane/EtOAc 6:4).

^{1}**H NMR** (400 MHz, $CDCl_3$): δ 7.64 (br s, 1H), 5.95 – 5.87 (m, 1H), 5.35 – 5.27 (m, 2H), 4.16 – 4.14 (m, 2H), 2.46 (s, 3H).

^{13}**C-NMR** (101 MHz, $CDCl_3$): δ 165.3, 156.4, 131.7, 118.8, 111.0, 45.0, 11.9.

HRMS (ESI): calculated for $C_7H_9N_3O_3$ ($[M]^+$) 183.0644; found 183.0637.

(3-Methyl-4-nitro-isoxazol-5-yl)-isopropylamine, 8h

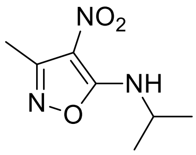

Yellow solid, 74 mg, 99% yield. Reaction time = 16 hours. R_f = 0.51 (pentane/EtOAc 6:4).

^{1}**H NMR** (400 MHz, $CDCl_3$): δ 7.40 (br s, 1H), 4.15 – 4.06 (m, 1H), 2.45 (s, 3H), 1.37 (d, *J* = 6.4 Hz, 6H).

^{13}**C-NMR** (101 MHz, $CDCl_3$): δ 164.6, 156.2, 110.7, 46.0, 23.0, 12.0.

HRMS (ESI): calculated for $C_7H_{11}N_3O_3$ ($[M]^+$) 185.0800; found 185.0806.

(3-Methyl-4-nitro-isoxazol-5-yl)-*sec*-butylamine, 8i

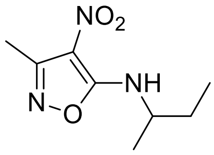

Yellow solid, 80 mg, 99% yield. Reaction time = 16 hours. R_f = 0.52 (pentane/EtOAc 6:4).

^{1}H NMR (400 MHz, $CDCl_3$): δ 7.39 (br s, 1H), 3.92 – 3.86 (m, 1H), 2.44 (s, 3H), 1.70 – 1.63 (m, 2H), 1.33 (d, *J* = 6.6 Hz, 3H), 0.96 (t, *J* = 7.4 Hz, 3H)

^{13}C-NMR (101 MHz, $CDCl_3$): δ 164.9, 156.2, 110.7, 51.5, 29.8, 20.7, 12.0, 10.3.

HRMS (ESI): calculated for $C_8H_{13}N_3O_3$ ($[M]^+$) 199.0957; found 199.0966.

(3-Methyl-4-nitro-isoxazol-5-yl)-cyclobutylamine, 8j

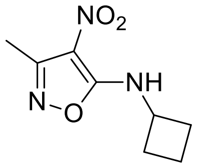

Brown solid, 79 mg, 99% yield. Reaction time = 16 hours. R_f = 0.51 (pentane/EtOAc 6:4).

^{1}H NMR (400 MHz, $CDCl_3$): δ 7.65 (br s, 1H), 4.39 – 4.33 (m, 1H), 2.48 – 2.44 (m, 2H), 2.44 (s, 3H), 2.20 – 2.10 (m, 2H), 1.87 – 1.78 (m, 2H).

^{13}C-NMR (101 MHz, $CDCl_3$): δ 164.2, 156.2, 110.7, 47.7, 31.1, 15.1, 12.0.

HRMS (ESI): calculated for $C_8H_{11}N_3O_3$ ($[M]^+$) 197.0800; found 197.0810.

(3-Methyl-4-nitro-isoxazol-5-yl)-pyrrolidine, 8k

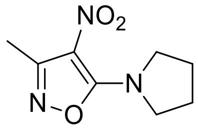

Brown solid, 79 mg, 99% yield. Reaction time = 18 hours. R_f = 0.29 (pentane/EtOAc 7:3).

^{1}H NMR (400 MHz, $CDCl_3$): δ 3.79 – 3.76 (m, 4H), 2.45 (s, 3H), 2.05 – 2.01 (m, 4H).

^{13}C-NMR (101 MHz, $CDCl_3$): δ 162.7, 158.1, 112.1, 50.6, 25.5, 13.0.

HRMS (ESI): calculated for $C_8H_{11}N_3O_3$ ($[M]^+$) 197.0800; found 197.0809.

(3-Methyl-4-nitro-isoxazol-5-yl)-piperidine, 8l

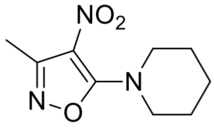

Brown oil, 83 mg, 97% yield. Reaction time = 18 hours. R_f = 0.29 (pentane/EtOAc 7:3).

^{1}H NMR (400 MHz, $CDCl_3$): δ 3.61 – 3.60 (m, 4H), 2.41 (s, 3H), 1.71 (br s, 6H).

^{13}C-NMR (101 MHz, $CDCl_3$): δ 164.4, 158.4, 112.6, 49.7, 25.6, 23.7, 13.2.

HRMS (ESI): calculated for $C_9H_{13}N_3O_3$ ($[M]^+$) 211.0957; found 251.0959.

(3-Methyl-4-nitro-isoxazol-5-yl)-morpholine, 8m

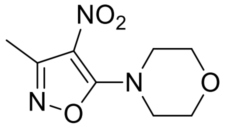

Brown solid, 85 mg, 98% yield. Reaction time = 18 hours. R_f = 0.26 (pentane/EtOAc 7:3).

^{1}H NMR (400 MHz, $CDCl_3$): δ 3.85 – 3.83 (m, 4H), 3.75 – 3.72 (m, 4H), 2.46 (s, 3H).

^{13}C-NMR (101 MHz, $CDCl_3$): δ 164.5, 158.6, 113.4, 66.4, 48.6, 13.2.

HRMS (ESI): calculated for $C_8H_{11}N_3O_4$ ($[M]^+$) 213.0750; found 213.0759.

[1-(3-Methyl-4-nitro-isoxazol-5-yl)-pyrrolidin-2-yl]-methanol, 8n

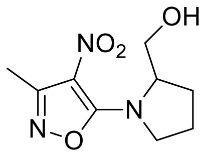

Brown solid, 90 mg, 97% yield. Reaction time = 18 hours. R_f = 0.26 (pentane/EtOAc 6:4).

^{1}H NMR (400 MHz, $CDCl_3$): δ 4.55 – 4.52 (m, 1H), 3.95 – 3.92 (m, 1H), 3.72 – 3.55 (m, 3H), 2.81 (br s, 1H), 2.38 (s, 3H), 2.13 – 2.10 (m, 3H), 1.94 – 1.91 (m, 1H).

^{13}C-NMR (101 MHz, $CDCl_3$): δ 162.6, 158.0, 112.5, 62.7, 62.3, 52.2, 27.7, 24.2, 12.9.

HRMS (ESI): calculated for $C_9H_{14}N_3O_4$ ($[M+H]^+$) 228.0984; found 228.0990.

1-(3-Methyl-4-nitro-isoxazol-5-yl)-pyrrolidine-2-carboxylic acid, 8o

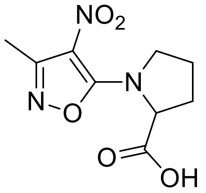

Brown solid, 93 mg, 95% yield. Reaction time = 18 hours. R_f = 0.11(pentane/EtOAc 6:4).

^{1}H NMR (400 MHz, $CDCl_3$): δ 9.35 (br s, 1H), 5.13 – 5.12 (m, 1H), 3.94 – 3.78 (m, 2H), 2.41 (s, 3H), 2.40 – 2.36 (m, 1H), 2.29 – 2.24 (m, 1H), 2.07 – 2.03 (m, 2H).

^{13}C NMR (101 MHz, $CDCl_3$): δ 175.7, 162.5, 158.1, 112.6, 62.3, 51.3, 30.7, 23.3, 12.9.

HRMS (ESI): calculated for $C_9H_{11}N_3O_5$ ($[M]^+$) 241.0699; found 241.0702.

(1*S*,2*S*)-2-((3-Methyl-4-nitroisoxazol-5-yl)amino)cyclohexanol, 8p

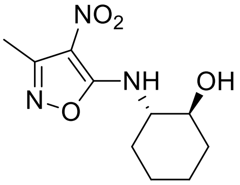

White solid, 91% yield. Reaction time = 18 hours. R_f = 0.48 (pentane/Et_2O/DCM 5:5:2)

^{1}H NMR (400 MHz, $CDCl_3$): δ 7.61 (br s, 1H), 3.69 – 3.62 (m, 1H), 3.59 – 3.53 (m, 1H), 2.45 (s, 3H), 2.19 – 2.09 (m, 2H), 1.82 – 1.75 (m, 2H), 1.46 – 1.32 (m, 4H).

^{13}C-NMR (101 MHz, $CDCl_3$): δ 165.6, 156.3, 111.2, 73.3, 59.6, 34.5, 31.8, 24.5, 24.2, 12.0.

HRMS (ESI): calculated for $C_{10}H_{16}N_3O_4$ ($[M+H]^+$) 242.1141; found 242.1138.

***Tert*-Butyl ((1*S*,2*S*)-2-((3-methyl-4-nitroisoxazol-5-yl)amino)cyclohexyl)carbamate, 8q**

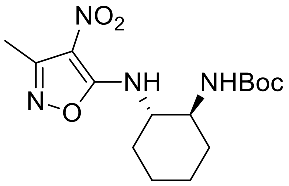

White solid, 93% yield. Reaction time = 18 hours. R_f = 0.55 (pentane/Et_2O/DCM 5:5:2)

^{1}H NMR (400 MHz, $CDCl_3$): δ 8.15 – 8.13 (m, 1H), 4.55 – 4.53 (m, 1H), 3.76 – 3.61 (m, 1H), 3.54 – 3.44 (m, 1H), 2.46 (s, 3H), 2.25 – 2.21 (m, 1H), 2.06 – 2.02 (m, 1H), 1.83 – 1.80 (m, 2H), 1.50 – 1.46 (m, 2H), 1.36 (s, 9H), 1.31 – 1.13 (m, 2H).

^{13}C-NMR (101 MHz, $CDCl_3$): δ 156.1, 156.5, 156.3, 110.8, 80.4, 59.9, 53.3, 32.4, 28.5, 28.3, 24.9, 24.4, 12.0.

HRMS (ESI): calculated for $C_{15}H_{25}N_4O_5$ ($[M+H]^+$) 341.1825; found 341.1820.

Benzyl ((1*S*,2*S*)-2-((3-methyl-4-nitroisoxazol-5-yl)amino)cyclohexyl)carbamate, 8r

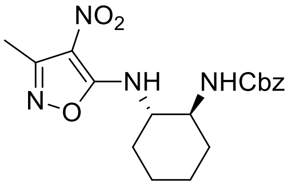

Light yellow solid, 90% yield. Reaction time = 18 hours. R_f = 0.67 (pentane/Et_2O/DCM 5:5:2)

^{1}H NMR (400 MHz, $CDCl_3$): δ 8.00 – 7.98 (m, 1H), 7.40 – 7.28 (m, 4H), 5.11 – 4.99 (m, 2H), 4.84 – 4.81 (m, 1H), 3.75 – 3.68 (m, 1H), 3.59 – 3.50 (m, 1H), 2.43 (s, 3H), 2.22 – 2.19 (m, 1H), 2.08 – 2.05 (m, 1H), 1.83 – 1.81 (m, 2H), 1.52 – 1.25 (m, 4H).

^{13}C-NMR (101 MHz, $CDCl_3$): δ 165.1, 156.9, 156.3, 136.2, 128.6, 128.4, 128.3, 110.8, 67.3, 59.2, 54.0, 32.6, 24.8, 24.4, 12.0.

HRMS (ESI): calculated for $C_{18}H_{23}N_4O_5$ ($[M+H]^+$) 375.1668; found 375.1677.

***Tert*-Butyl (2-(2-(2-((3-methyl-4-nitroisoxazol-5-yl)amino)ethoxy)ethoxy)ethyl)carbamate, 8s**

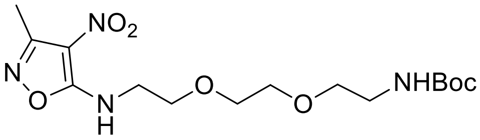

Yellow oil, 143 mg, 94% yield. Reaction time = 12 hours. R_f = 0.15 (pentane/Et_2O/DCM 1:1:1)

^{1}H NMR (400 MHz, $CDCl_3$): δ 4.98 (br s, 1H), 3.73 (s, 4H), 3.68 – 3.60 (m, 4H), 3.56 – 3.53 (m, 2H), 3.34 – 3.30 (m, 2H), 2.47 (s, 3H), 1.43 (s, 9H).

^{13}C-NMR (101 MHz, $CDCl_3$): δ 165.3, 156.5, 156.1, 111.0, 79.4, 70.7, 70.6, 70.3, 68.9, 42.5, 40.5, 28.5, 12.0.

HRMS (ESI): calculated for $C_{15}H_{26}N_4O_7$ ($[M]^+$) 374.1801; found 374.1799.

Application of 7a as N-protecting reagent for the orthogonal N-protection of diamines

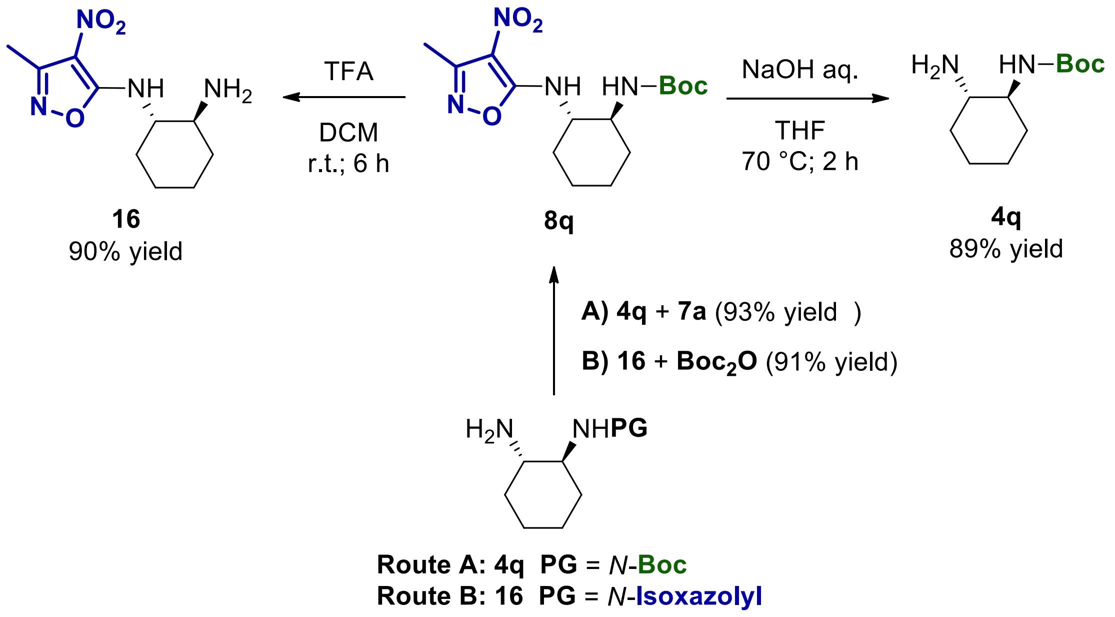

Scheme 4. Orthogonal deprotection of 1,2-diaminocyclohexane-derived *N*-Boc,*N*[1]-isoxazolyl diamine **8q**.

Base-promoted orthogonal N-deprotection of 8q to 4q

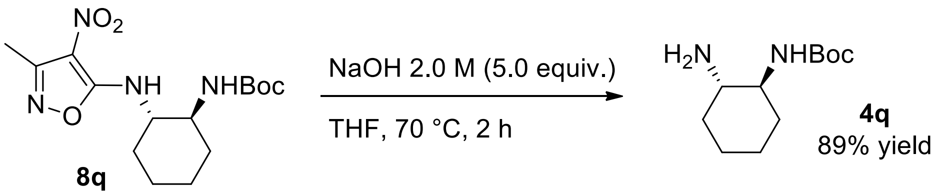

In a 5 mL glass test tube, **8q** (34 mg, 0.1 mmol) was dissolved in THF (0.4 mL) under stirring at room temperature. NaOH 2.0 M solution in H_2O (0.25 mL, 0.5 mmol) was then added in one portion. The tube was sealed and the reaction mixture heated to 70 °C in an oil bath, and vigorously stirred at that temperature for 2 hours. The crude mixture was diluted with DCM (5 mL) and water (5 mL), the phases separated and the aqueous phase extracted with DCM (3 x 5 mL). The combined organic layers were dried over sodium sulfate, filtered and the solvent evaporated under reduced pressure to obtain pure **4q** (19 mg, 89% yield).

***Tert*-Butyl ((1*S*,2*S*)-2-aminocyclohexyl)carbamate, 4q**

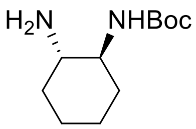

Yellow oil, 89% yield, R_f = 0.10 (DCM/MeOH 20:1)

¹H NMR (400 MHz, $CDCl_3$): δ 4.48 (br s, 1H), 3.12 – 3.10 (m, 1H), 2.30 (td, *J* = 10.4, 4.0 Hz, 1H), 2.00 – 1.93 (m, 2H), 1.70 – 1.67 (m, 2H), 1.43 (s, 9H), 1.27 – 1.09 (m, 5H).

¹³C-NMR (101 MHz, $CDCl_3$): δ 156.3, 79.4, 57.8, 55.8, 55.2, 35.4, 33.1, 28.5, 25.3.

HRMS (ESI): calculated for $C_{11}H_{23}N_2O_2$ ($[M+H]^+$) 215.1760; found 215.1765.

Acid-promoted orthogonal N-deprotection of 8q to 16

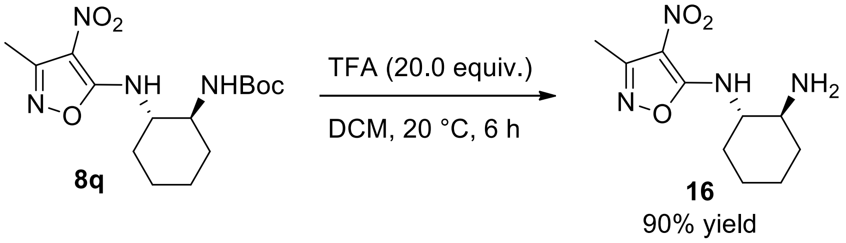

In a 5 mL glass test tube, **8q** (34 mg, 0.1 mmol) was dissolved in DCM (0.5 mL) under stirring at room temperature. TFA (153 uL, 2.0 mmol) was then added dropwise and the mixture was stirred at room temperature for 6 hours. The reaction was then quenched by dropwise addition of saturated $NaHCO_3$ (1.0 mL), followed by water (5 mL) and DCM (5 mL). The layers were separated and the aqueous phase extracted with DCM (3 x 5 mL). The combined organic layers were dried over sodium sulfate, filtered and the solvent evaporated under reduced pressure to obtain pure **16** (22 mg, 90% yield).

(1*S*,2*S*)-*N*¹-(3-Methyl-4-nitroisoxazol-5-yl)cyclohexane-1,2-diamine, 16

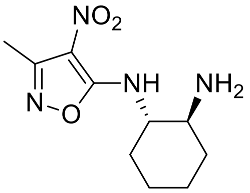

White solid, 90% yield, R_f = 0.12 (DCM/MeOH 20:1)

¹H NMR (400 MHz, $CDCl_3$): δ 7.64 (br s, 1H), 3.48 – 3.41 (m, 1H), 2.66 (td, *J* = 10.4, 4.1 Hz, 1H), 2.46 (s, 3H), 2.18 – 2.14 (m, 1H), 2.03 – 2.00 (m, 1H), 1.82 – 1.75 (m, 2H), 1.45 – 1.20 (m, 5H).

¹³C-NMR (101 MHz, $CDCl_3$): δ 165.5, 156.3, 111.1, 60.5, 55.1, 35.9, 32.6, 24.9, 24.9, 12.0.

HRMS (ESI): calculated for $C_{10}H_{17}N_4O_3$ ($[M+H]^+$) 241.1301; found 241.1308.

Orthogonal *N*-deprotection of *N*-Boc,*N*¹-isoxazolyl diamine 8s

The general nature of the orthogonal *N*-deprotection strategy developed for the 1,2-DACH-derived diamine **8q**, was demonstrated by the selective *N*-deprotection of the linear *N*-Boc,*N*¹-isoxazolyl diamine **8s** derived from polyethylenglycol, to produce compounds **4s** or **17** (Scheme S5).

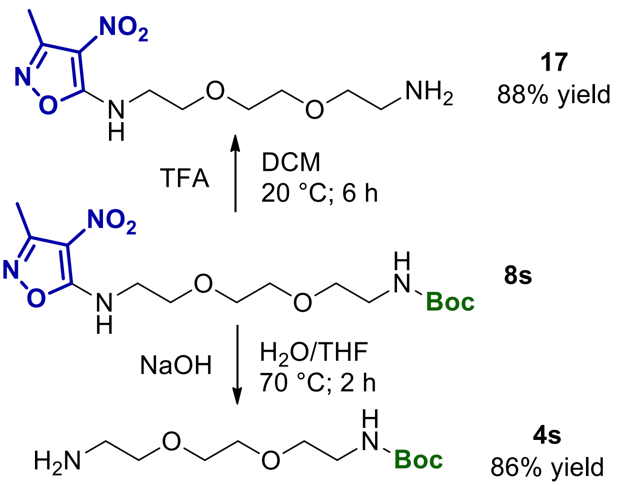

Scheme S5. Orthogonal *N*-deprotection of the PEG-derived linear diamine **8s**.

tert-Butyl (2-(2-(2-aminoethoxy)ethoxy)ethyl)carbamate, 4s

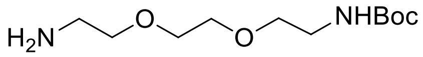

Yellow oil, 21 mg, 86% yield, R_f = 0.13 (DCM/MeOH 30:1) and

¹H NMR (400 MHz, $CDCl_3$): δ 5.15 (br s, 1H), 3.61 – 3.59 (m, 4H), 3.53 – 3.47 (m, 4H), 3.31 – 3.27 (m, 2H), 2.85 (t, *J* = 5.2 Hz, 2H), 1.49 – 1.27 (m, 2H), 1.41 (s, 9H).

¹³C-NMR (101 MHz, $CDCl_3$): δ 156.1, 79.2, 73.6, 70.4, 70.3, 70.3, 41.9, 40.4, 28.5.

HRMS (ESI): calculated for $C_{11}H_{25}N_2O_4$ ($[M+H]^+$) 249.1814; found 249.1819.

*N*¹-(2-(2-(2-Aminoethoxy)ethoxy)ethyl)-3-methyl-4-nitroisoxazol-5-amine, 17

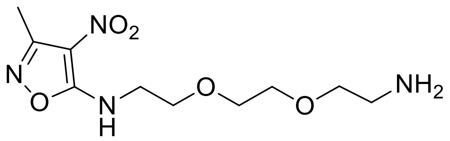

Yellow oil, 24 mg, 88% yield, R_f = 0.27 (DCM/MeOH 25:1)

¹H NMR (400 MHz, $CDCl_3$): δ 4.05 (br s, 2H), 3.72 – 3.66 (m, 4H), 3.65 – 3.60 (m, 6H), 3.54 – 3.48 (m, 2H), 2.85 (br s, 1H), 2.42 (s, 3H).

¹³C-NMR (101 MHz, $CDCl_3$): δ 165.2, 156.4, 110.9, 73.2, 70.6, 70.2, 68.8, 42.4, 41.5, 11.9.

HRMS (ESI): calculated for $C_{10}H_{19}N_4O_5$ ($[M+H]^+$) 275.2817; found 275.2810.

Detailed synthetic method example at 5 mmol scale: (3-Methyl-4-nitro-isoxazol-5-yl)-phenylamine, 8a

3-methyl-4-nitro-5-trichloromethylisoxazole **7a** (1,230 g, 5 mmol) was dissolved in THF (50 mL) in a 100 mL round bottomed flask at room temperature, avoiding any precautions to exclude moisture and air. Potassium carbonate (700 mg, 2 mmol) was added to the solution followed by the amine **4a-s** (5.5 mmol, 1.1 equiv.). The round bottomed flak was charged with a magnetic follower, sealed with a rubber septum, heated to 50 °C and stirred at this temperature for the indicated time. The reaction mixture was partitioned between H_2O (100 mL) and DCM (100 mL) and the aqueous phase extracted with DCM (3 x 50 mL). The combined organic phases were dried over sodium sulfate, filtered and the solvent removed under reduced pressure to obtain 1,060 g of pure products **8a**, 97% yield.

NMR Spectra

7a ^{1}H-NMR (400 MHz, $CDCl_3$)

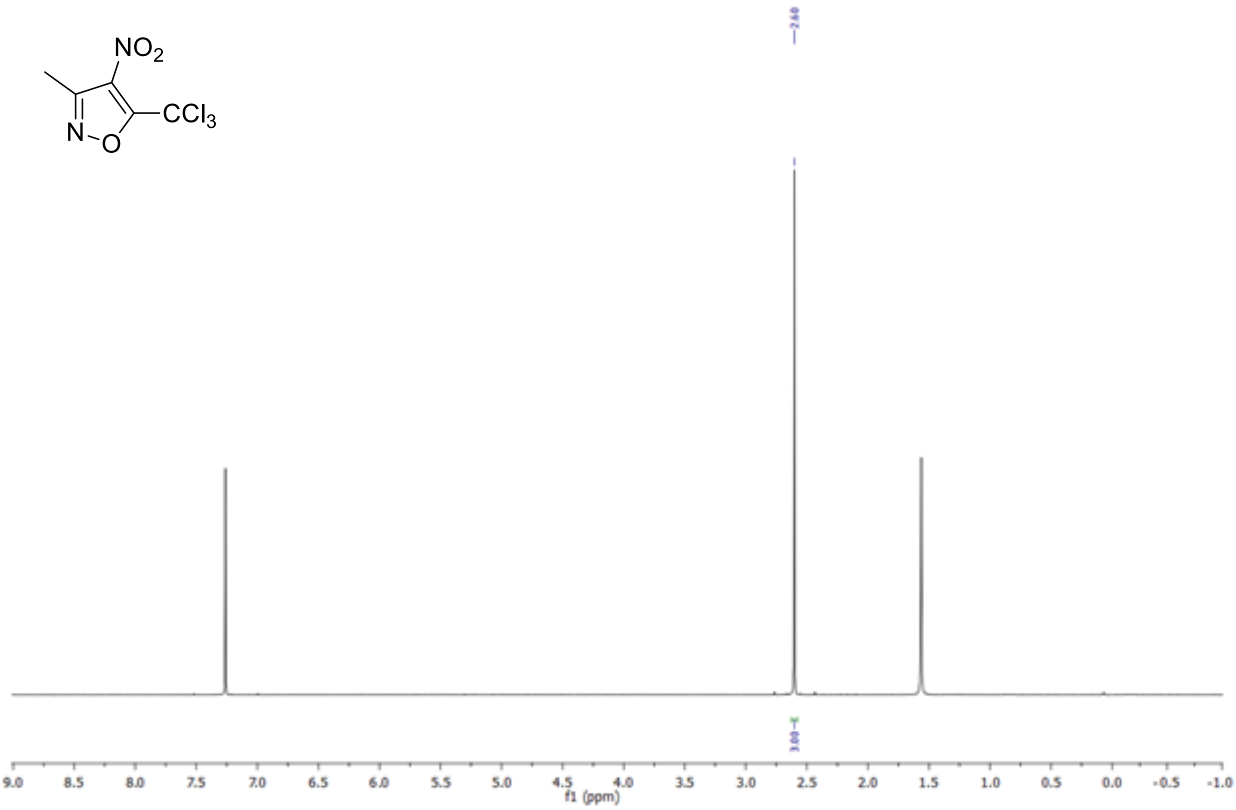

7a ^{13}C-NMR (101 MHz, $CDCl_3$)

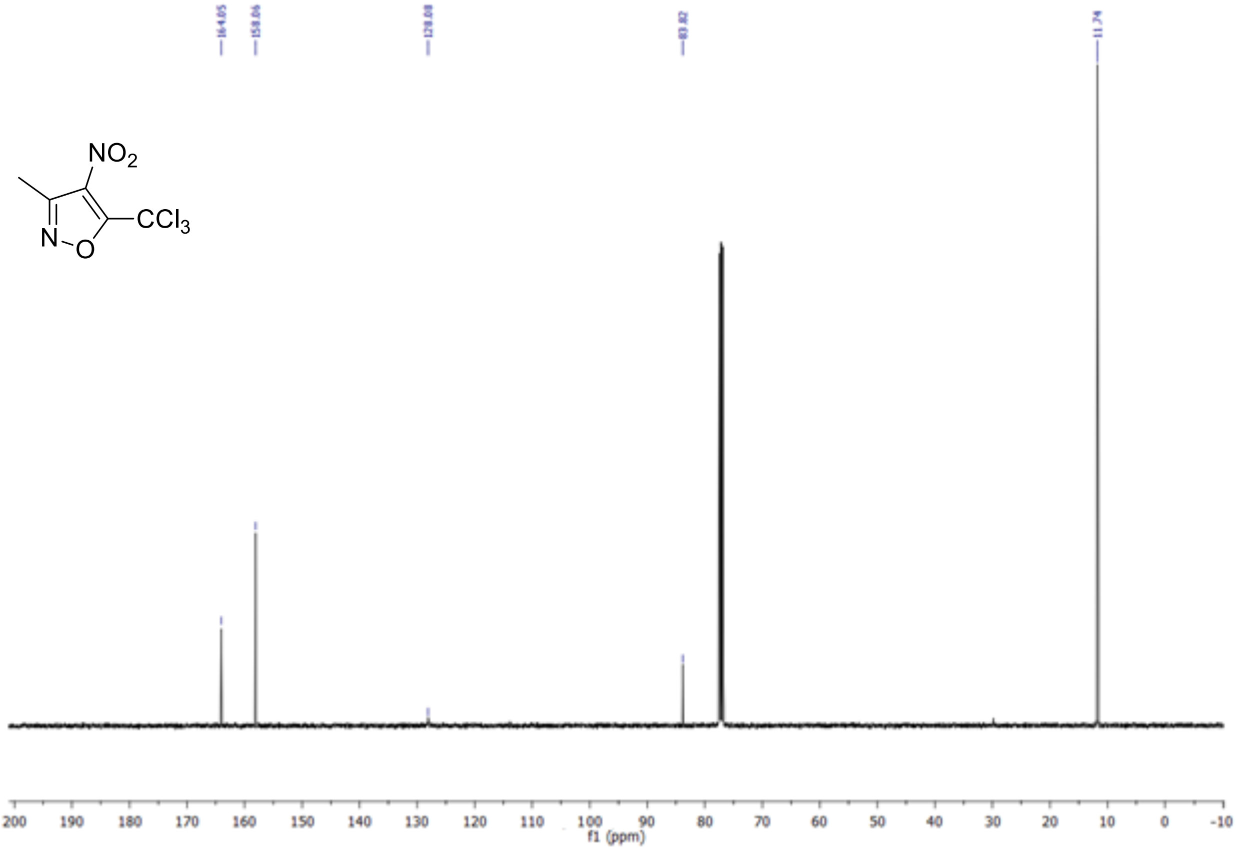

7b ^{1}H-NMR (400 MHz, $CDCl_3$)

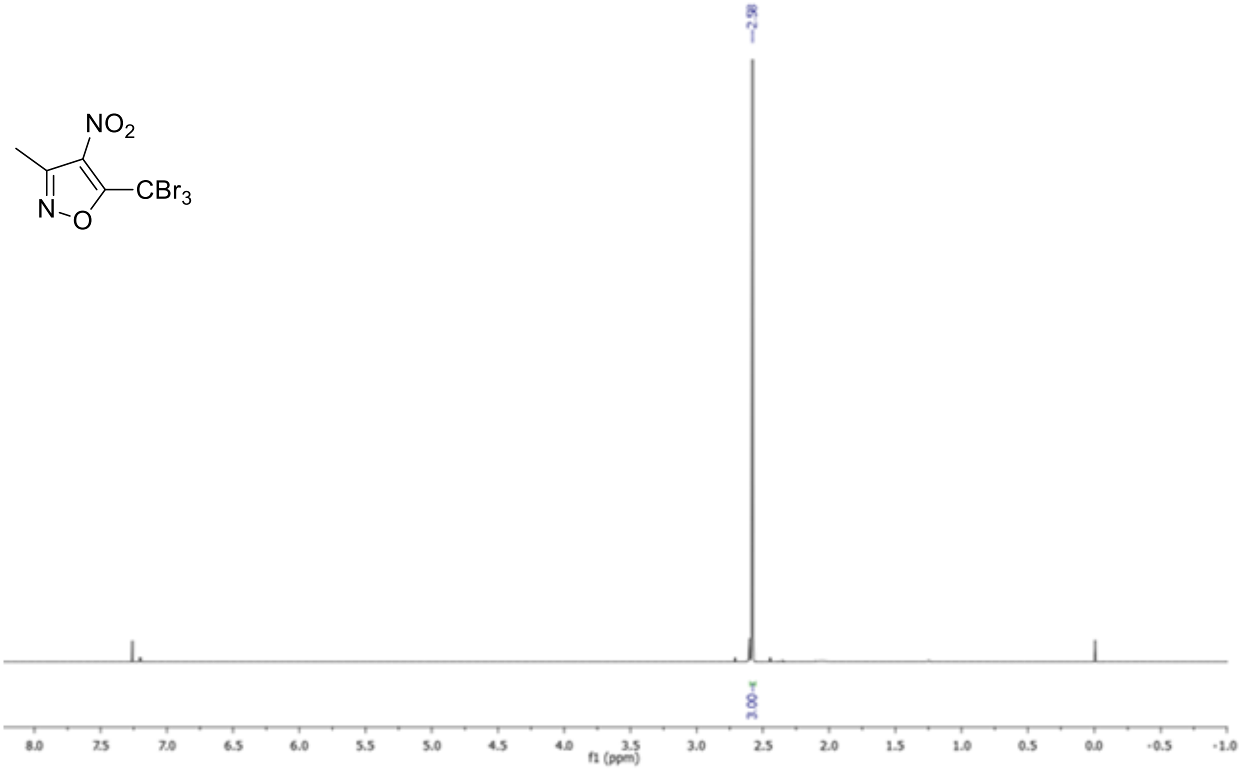

7b ^{13}C-NMR (101 MHz, $CDCl_3$)

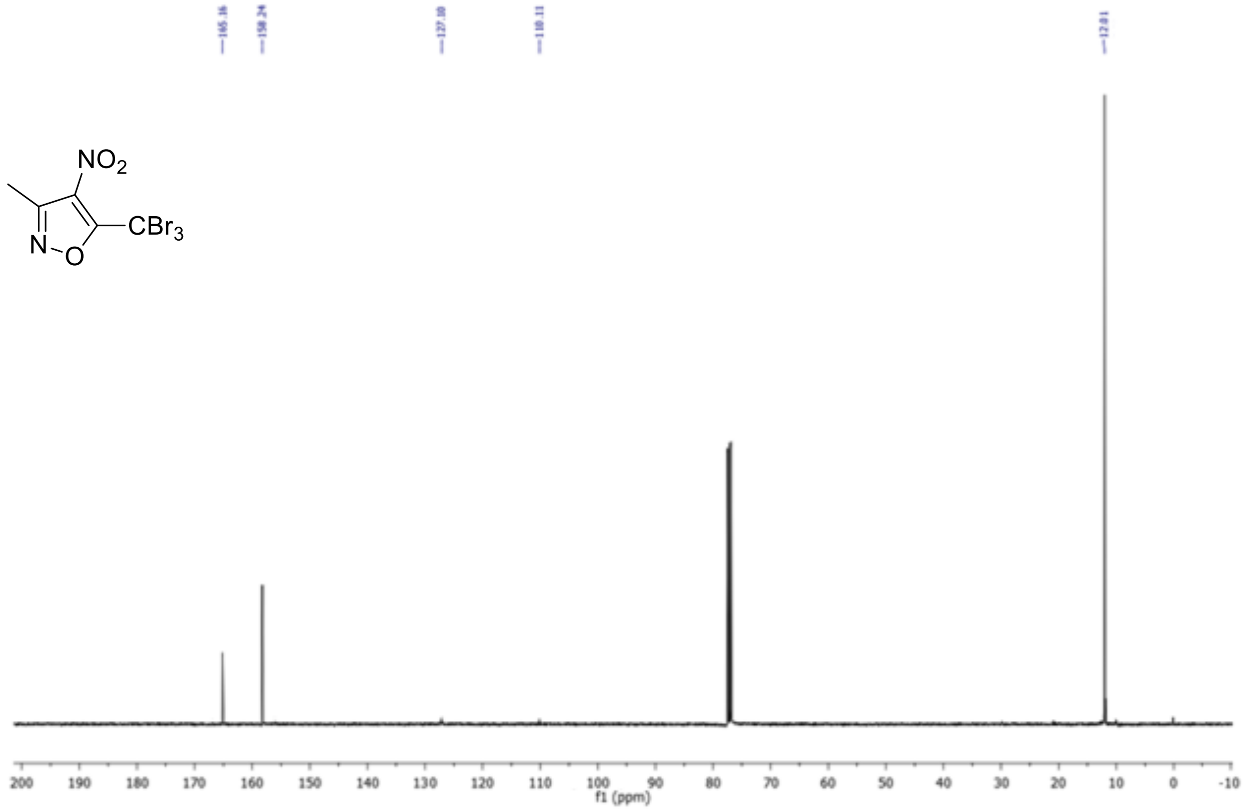

7b’’ ^{1}H-NMR (400 MHz, $CDCl_3$)

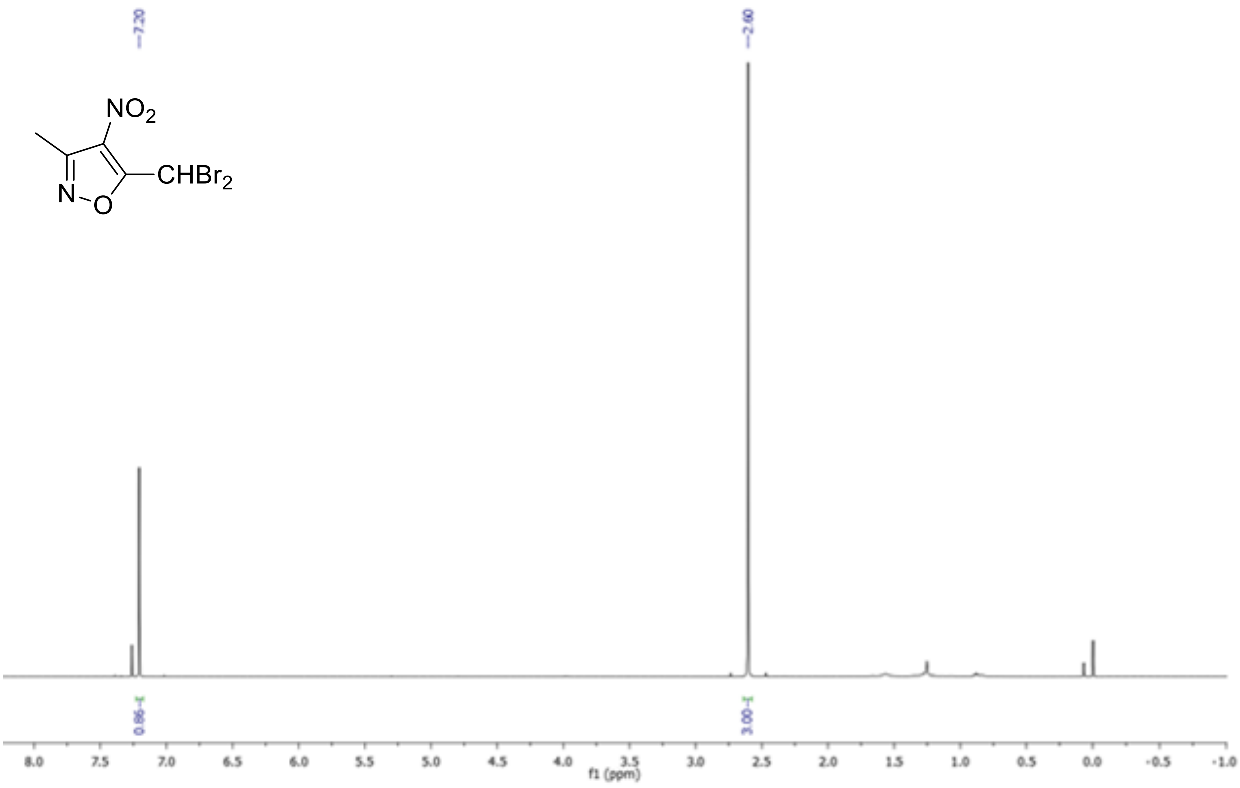

7b’’ ^{13}C-NMR (101 MHz, $CDCl_3$)

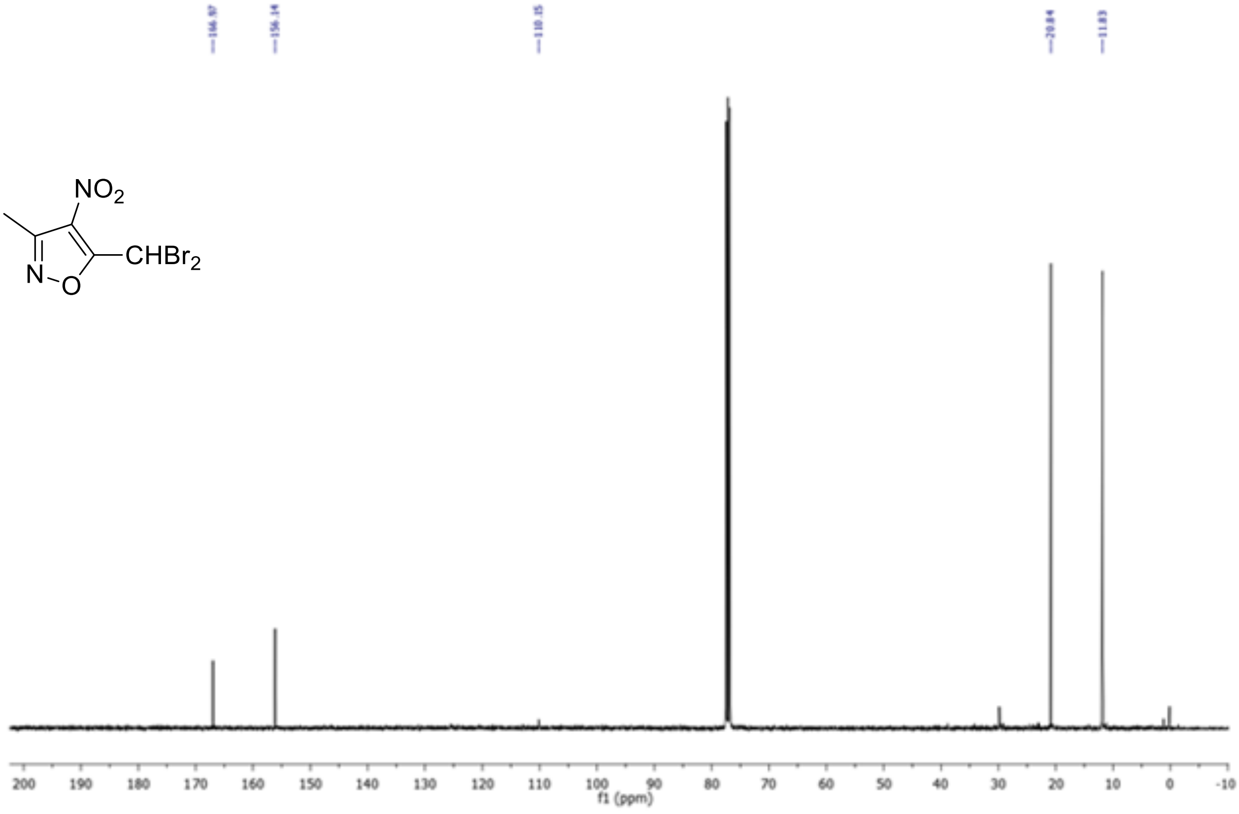

8a ^{1}H-NMR (400 MHz, $CDCl_3$)

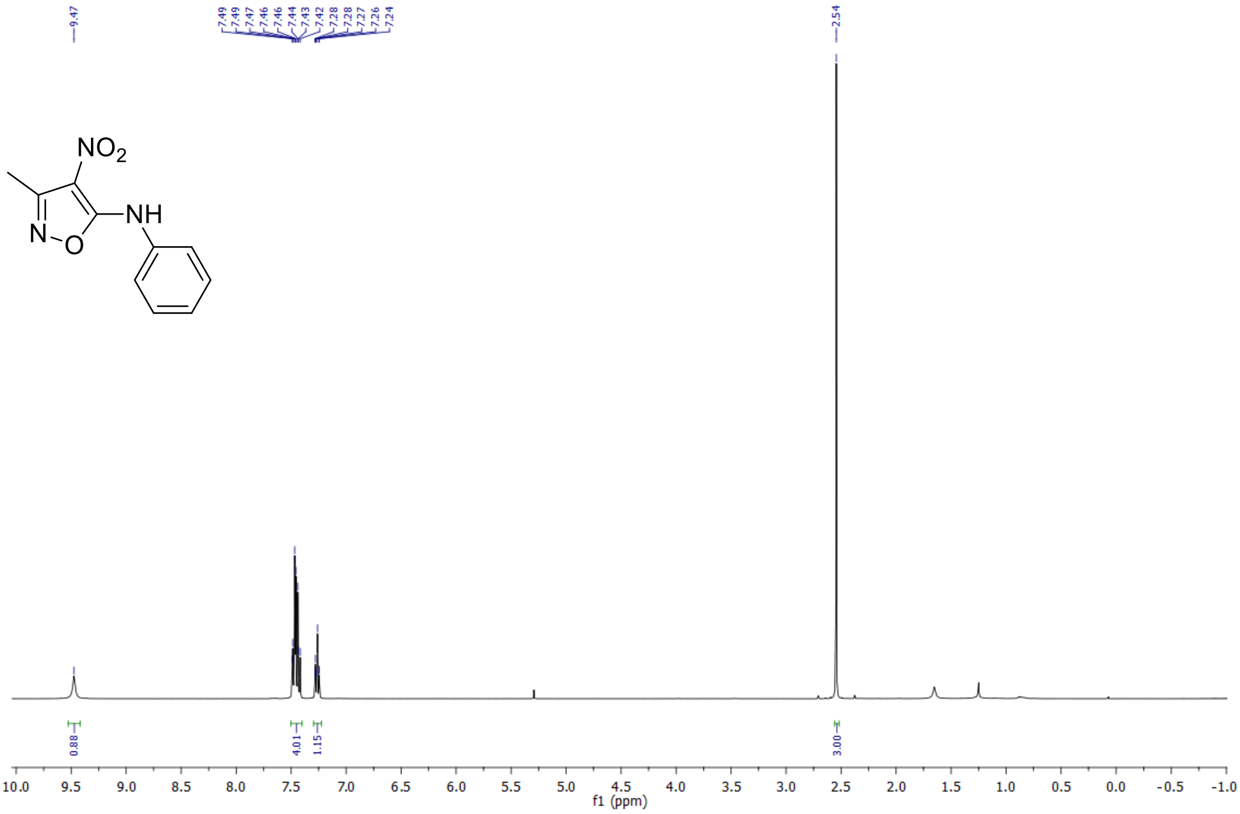

8a ^{13}C-NMR (101 MHz, $CDCl_3$)

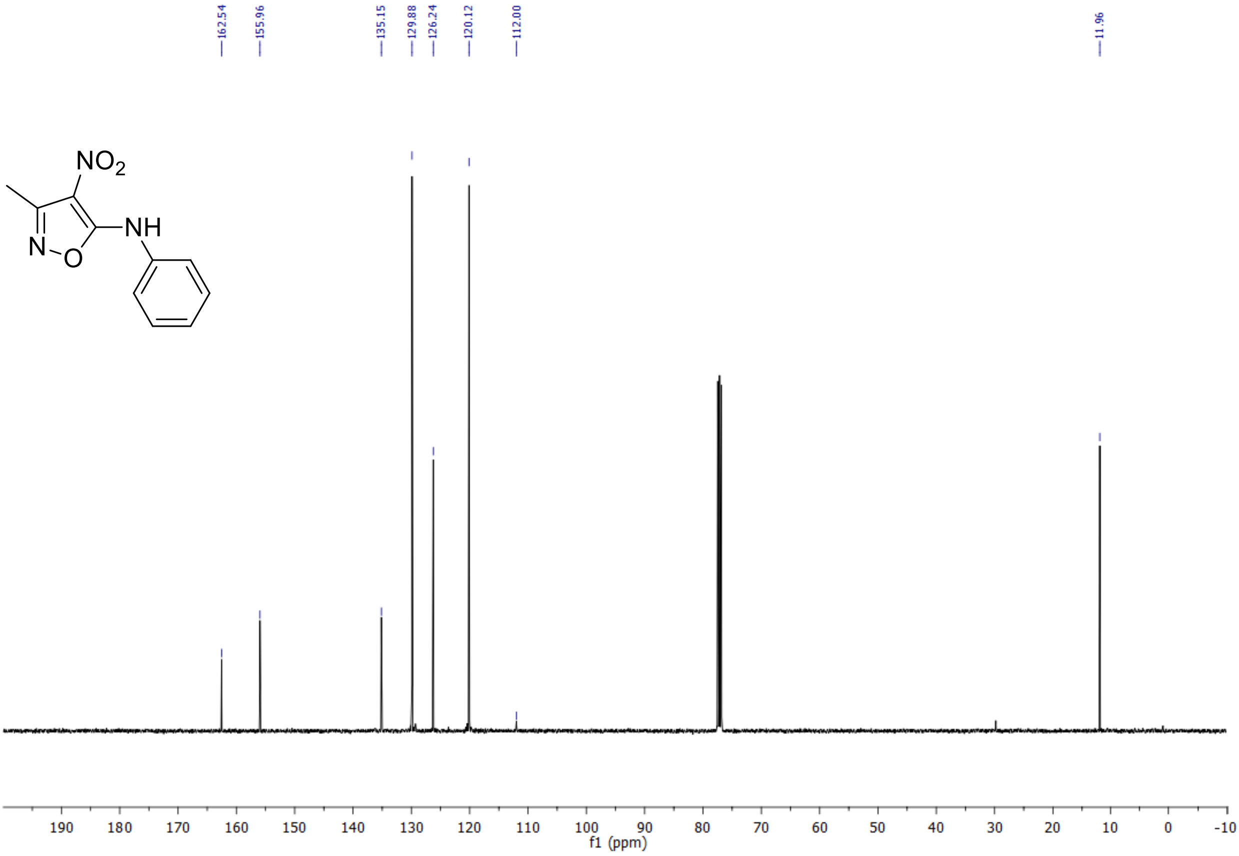

8b ^{1}H-NMR (400 MHz, $CDCl_3$)

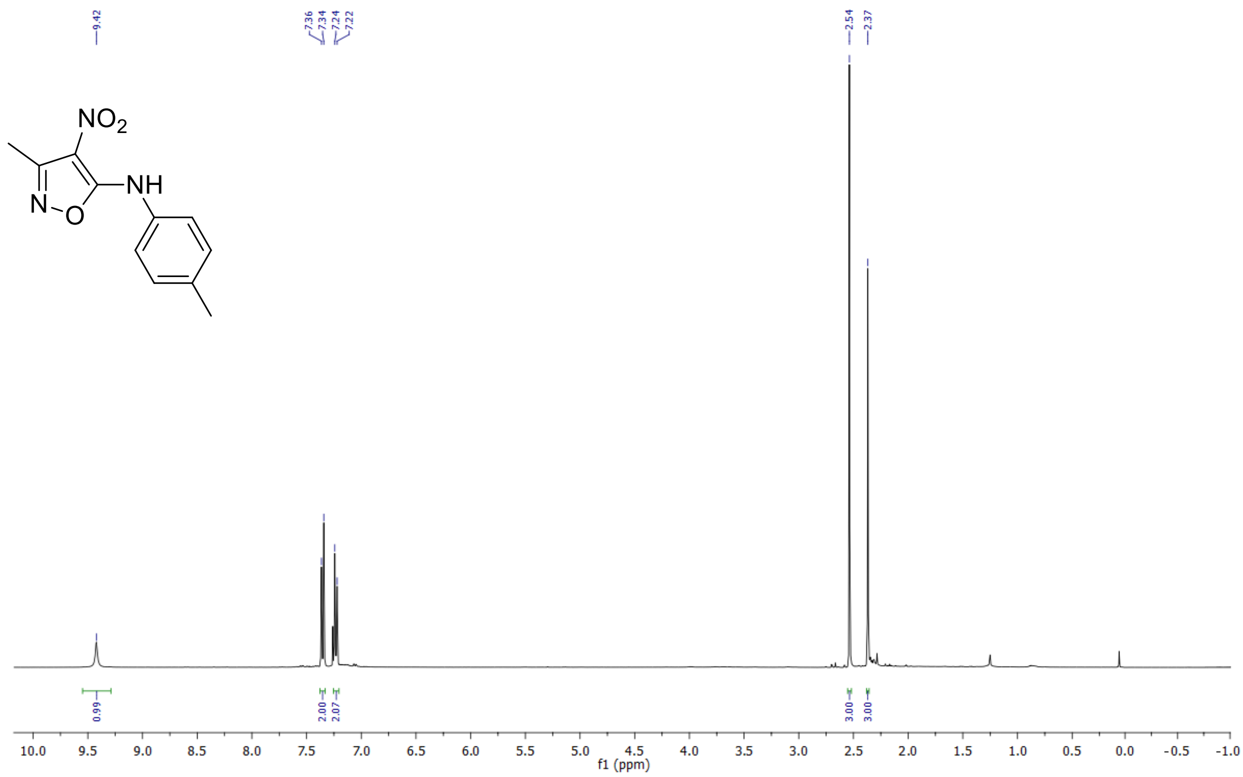

8b ^{13}C-NMR (101 MHz, $CDCl_3$)

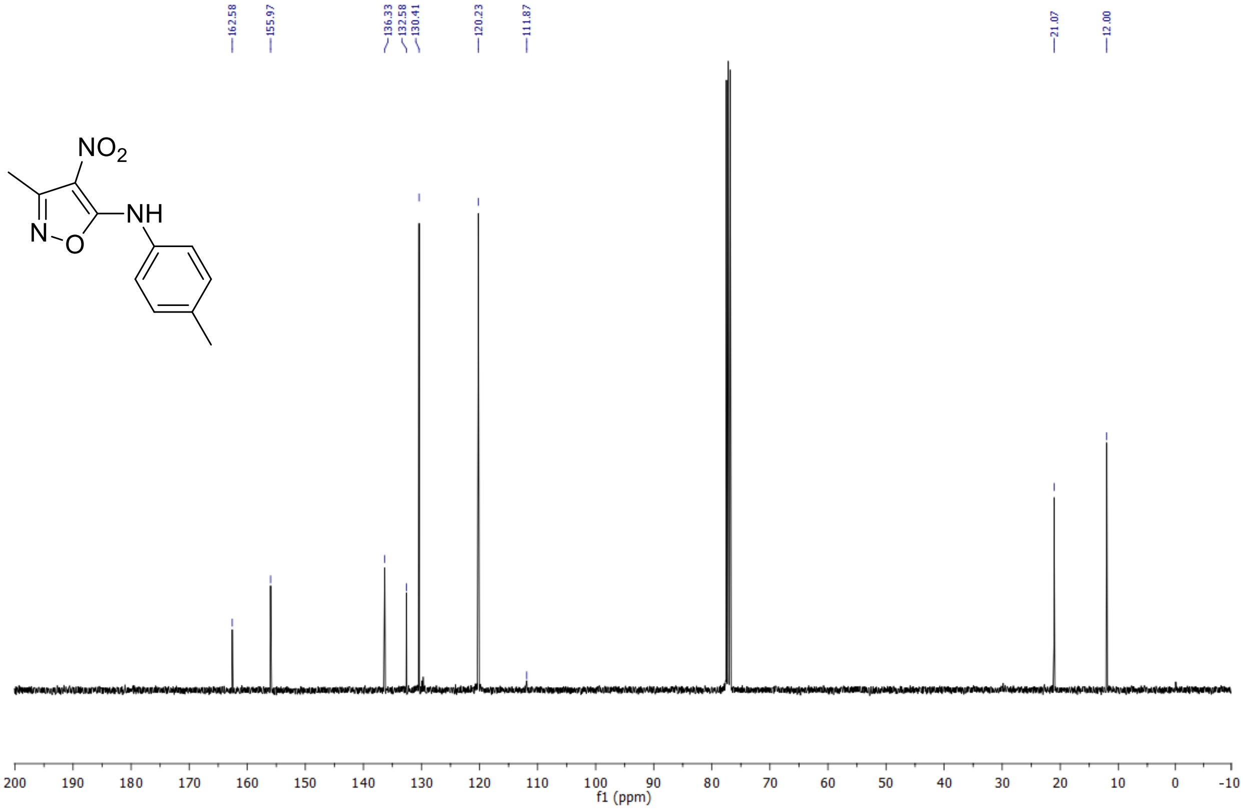

8c ^{1}H-NMR (400 MHz, CDCl$_3$)

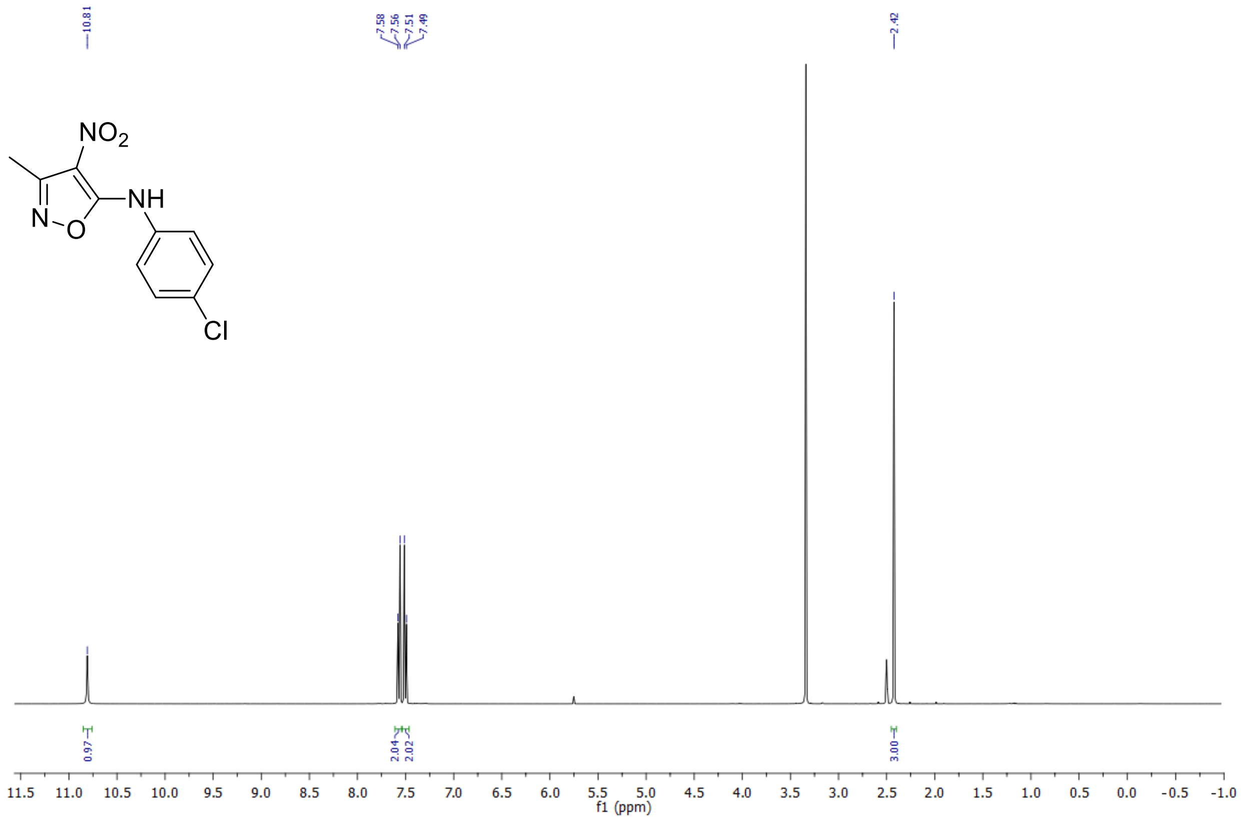

8c ^{13}C-NMR (101 MHz, CDCl$_3$)

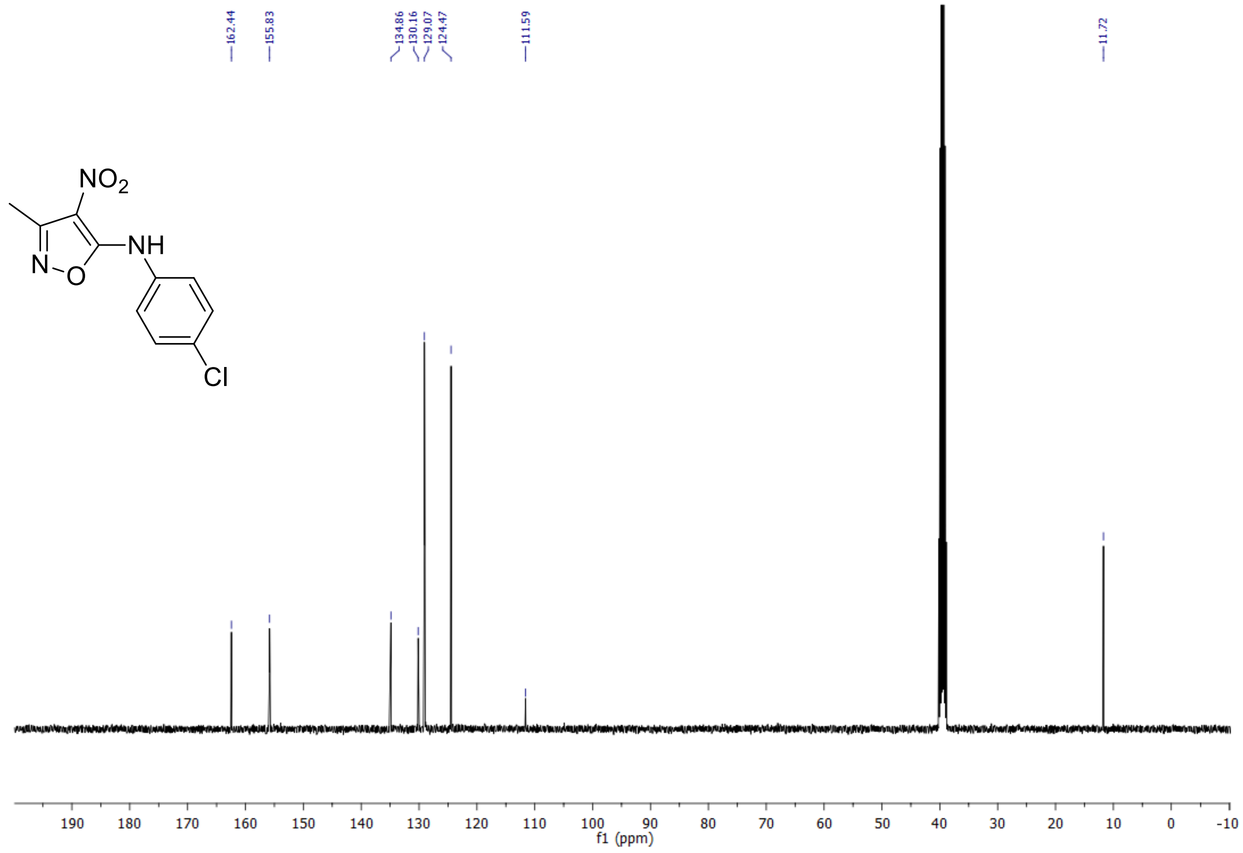

8d ^{1}H-NMR (400 MHz, $CDCl_3$)

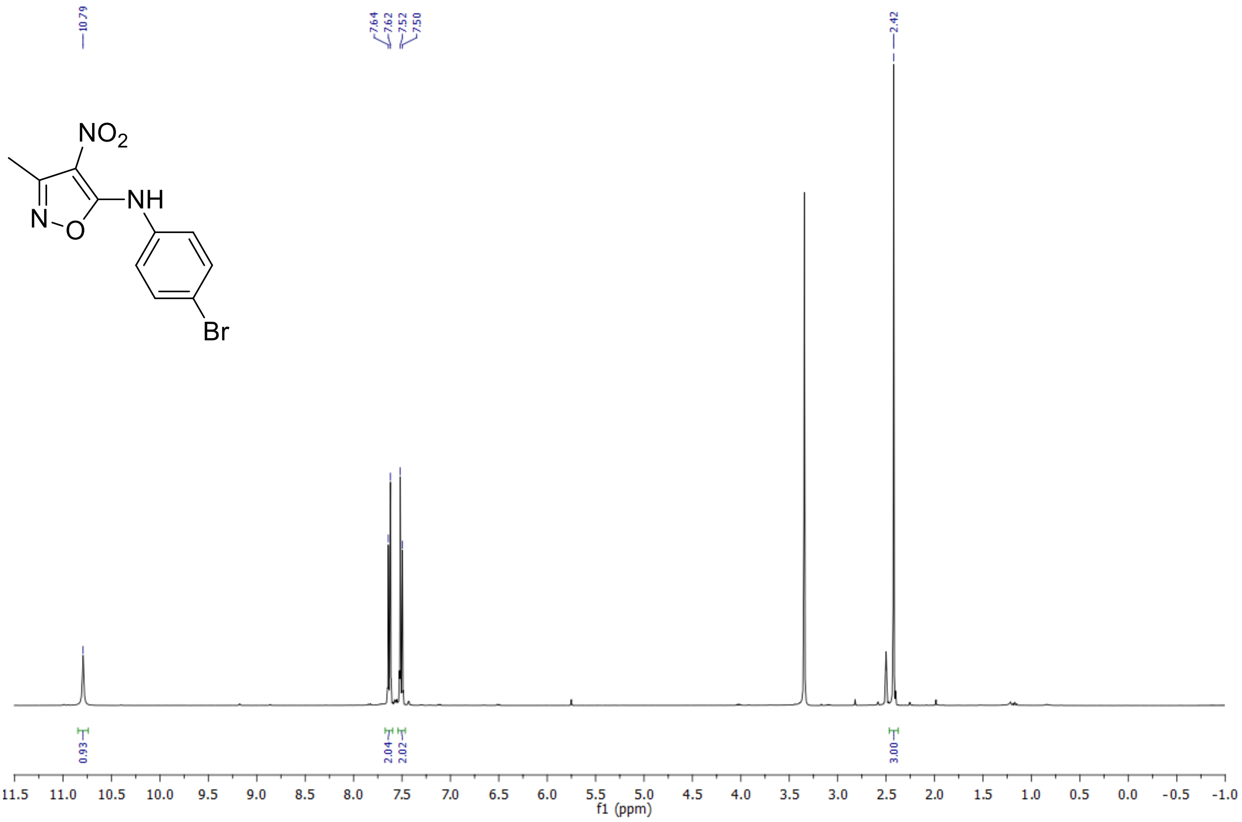

8d ^{13}C-NMR (101 MHz, $CDCl_3$)

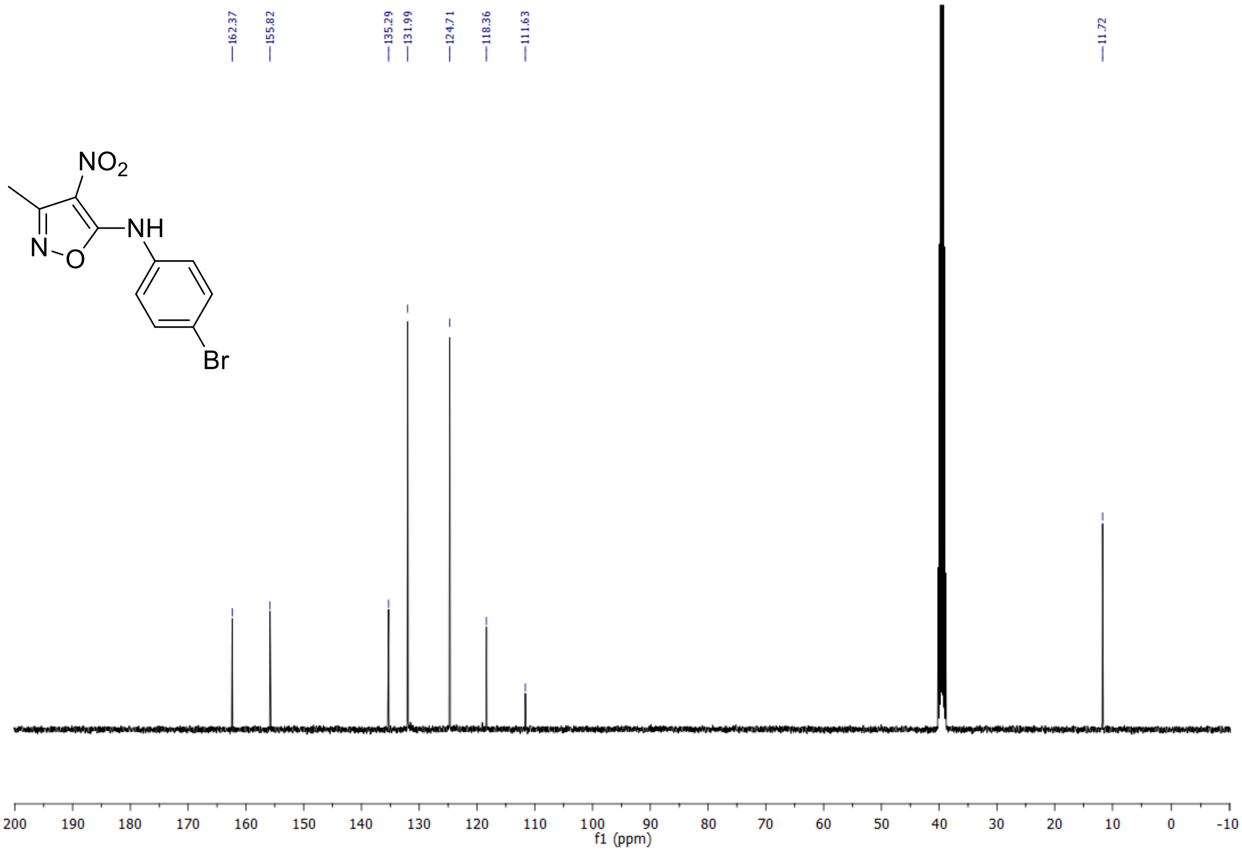

8e ^{1}H-NMR (400 MHz, $CDCl_3$)

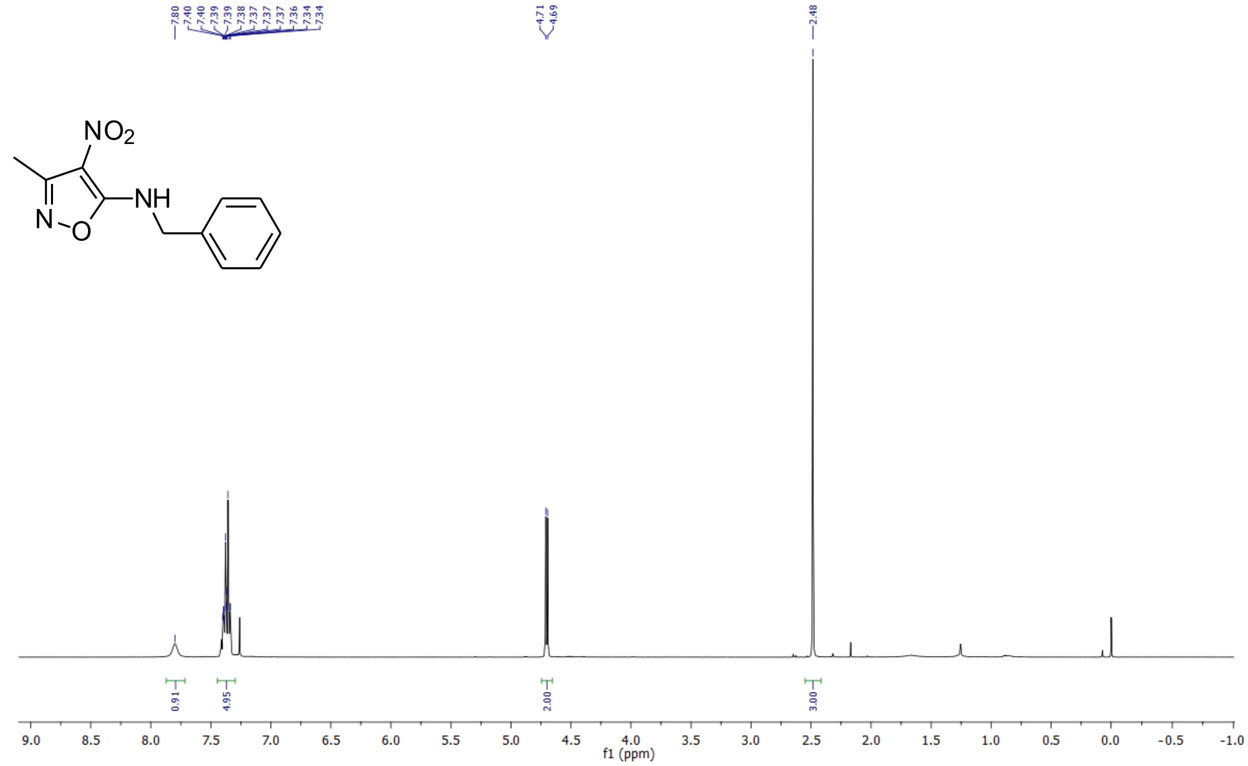

8e ^{13}C-NMR (101 MHz, $CDCl_3$)

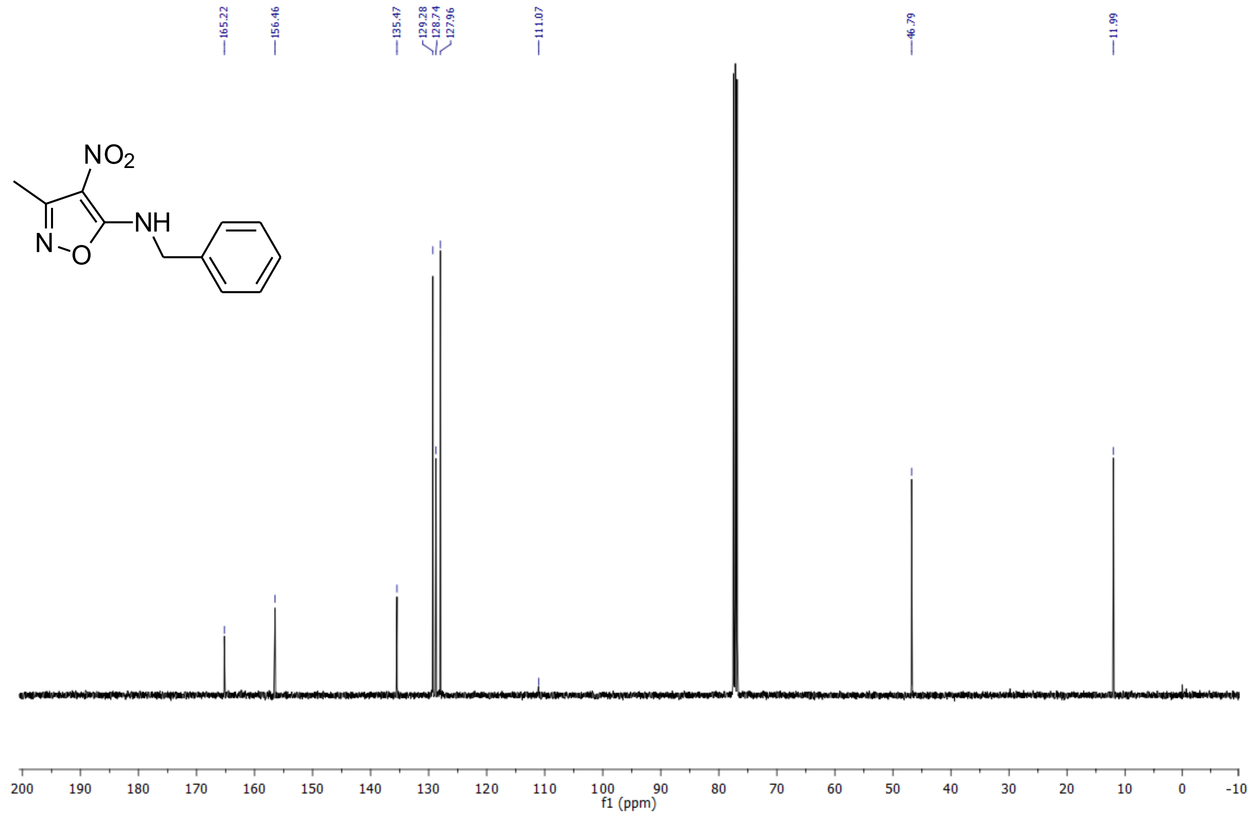

8f ^{1}H-NMR (400 MHz, $CDCl_3$)

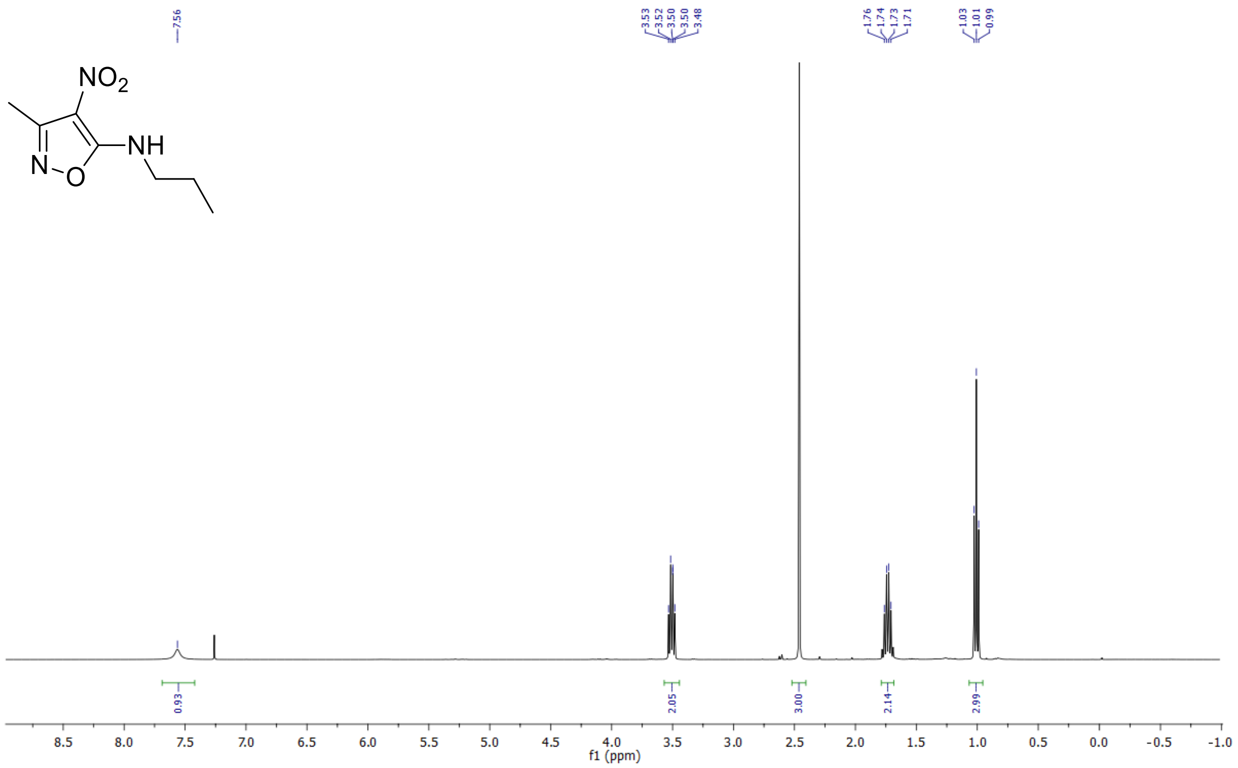

8f ^{13}C-NMR (101 MHz, $CDCl_3$)

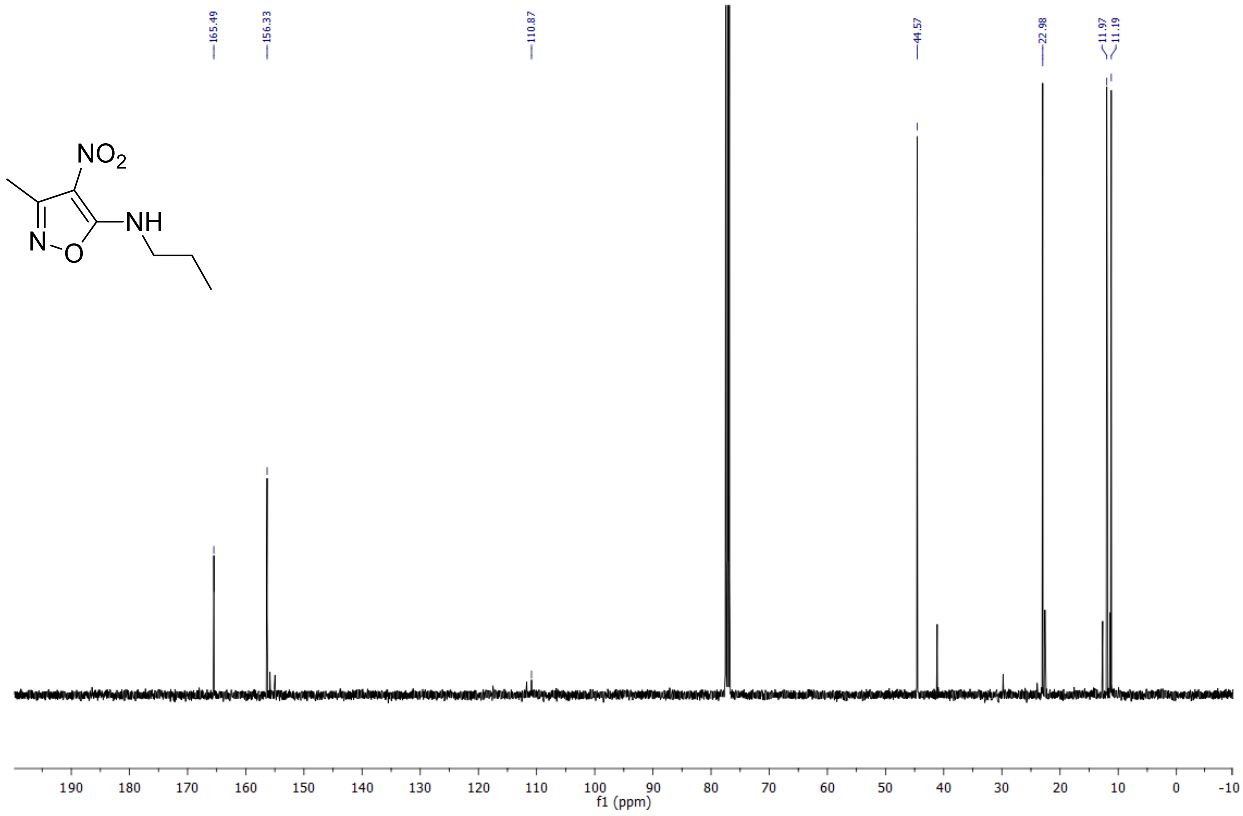

8g ^{1}H-NMR (400 MHz, $CDCl_3$)

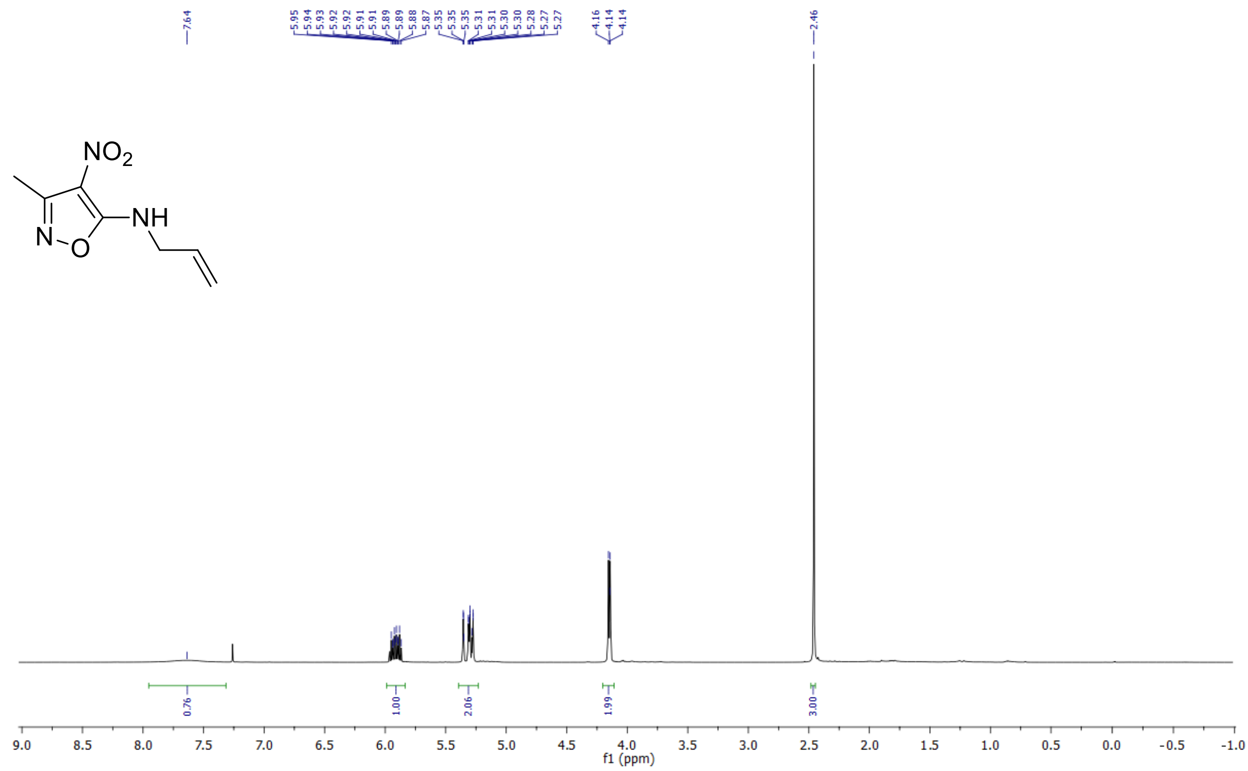

8g ^{13}C-NMR (101 MHz, $CDCl_3$)

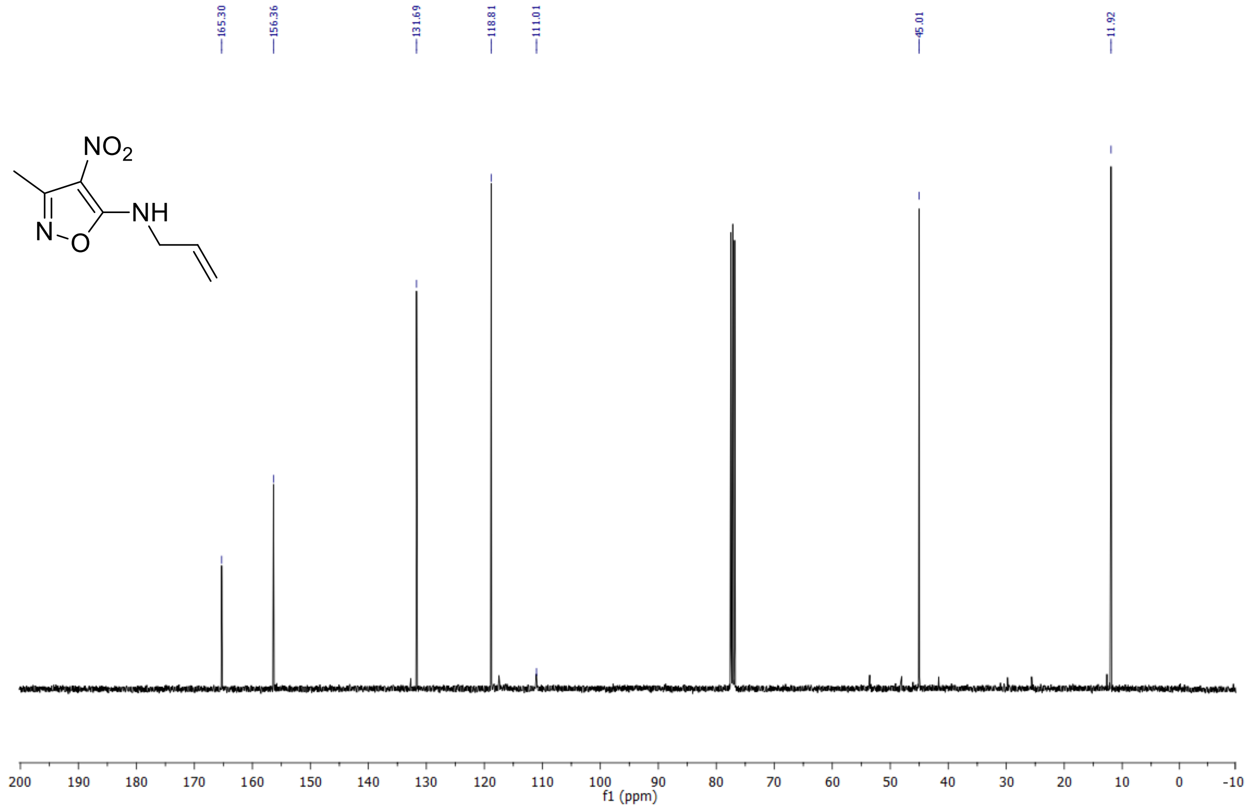

8h ^{1}H-NMR (400 MHz, $CDCl_3$)

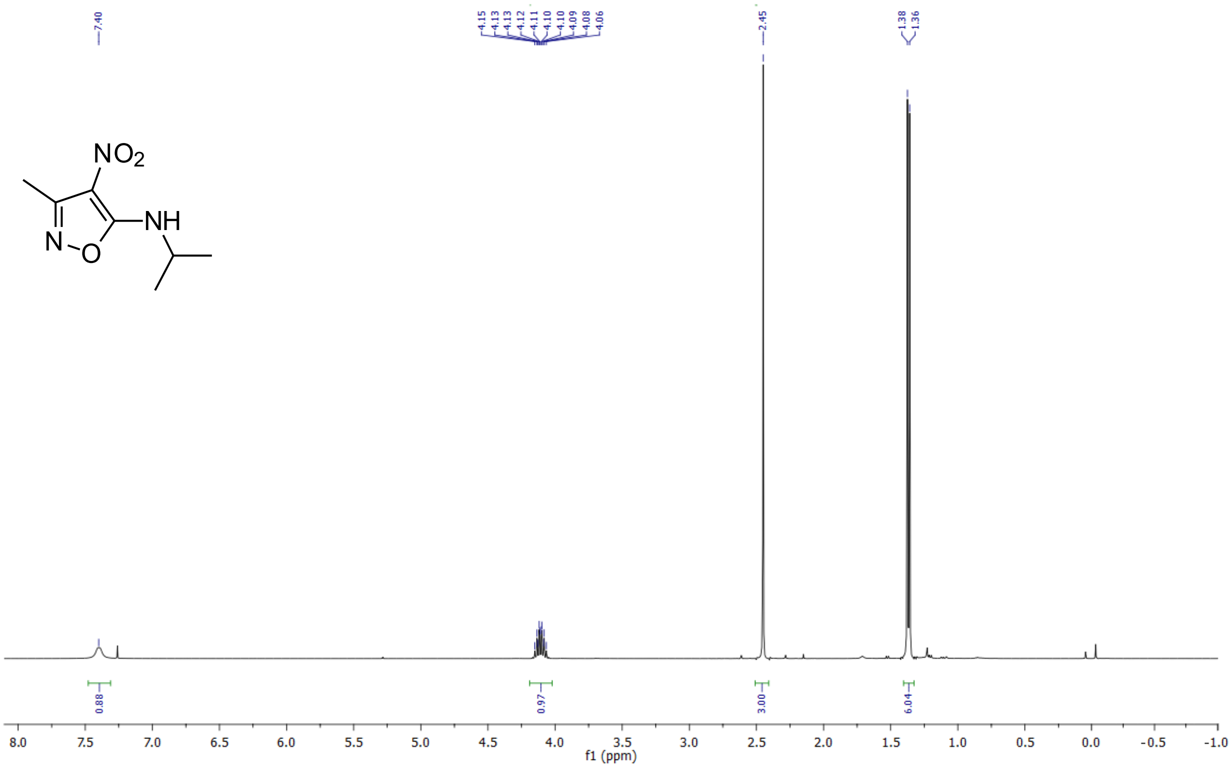

8h ^{13}C-NMR (101 MHz, $CDCl_3$)

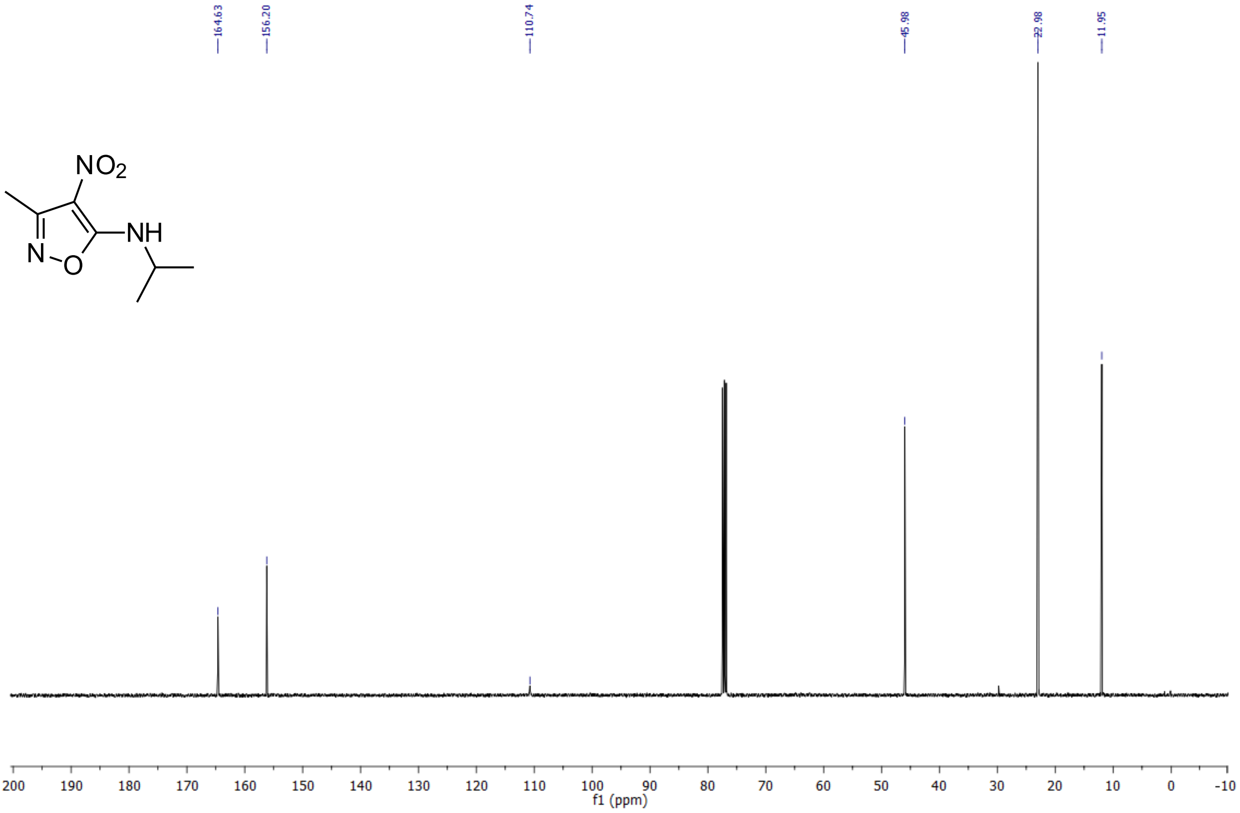

8i ^{1}H-NMR (400 MHz, $CDCl_3$)

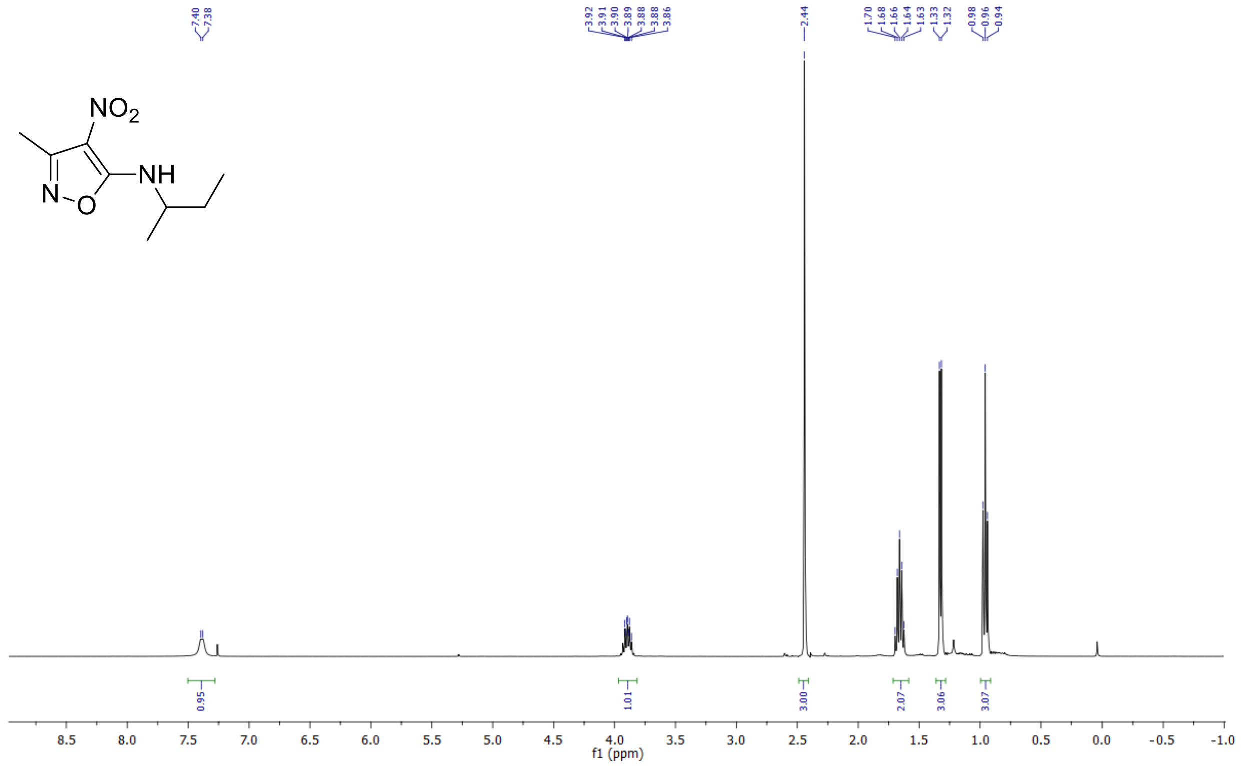

8i ^{13}C-NMR (101 MHz, $CDCl_3$)

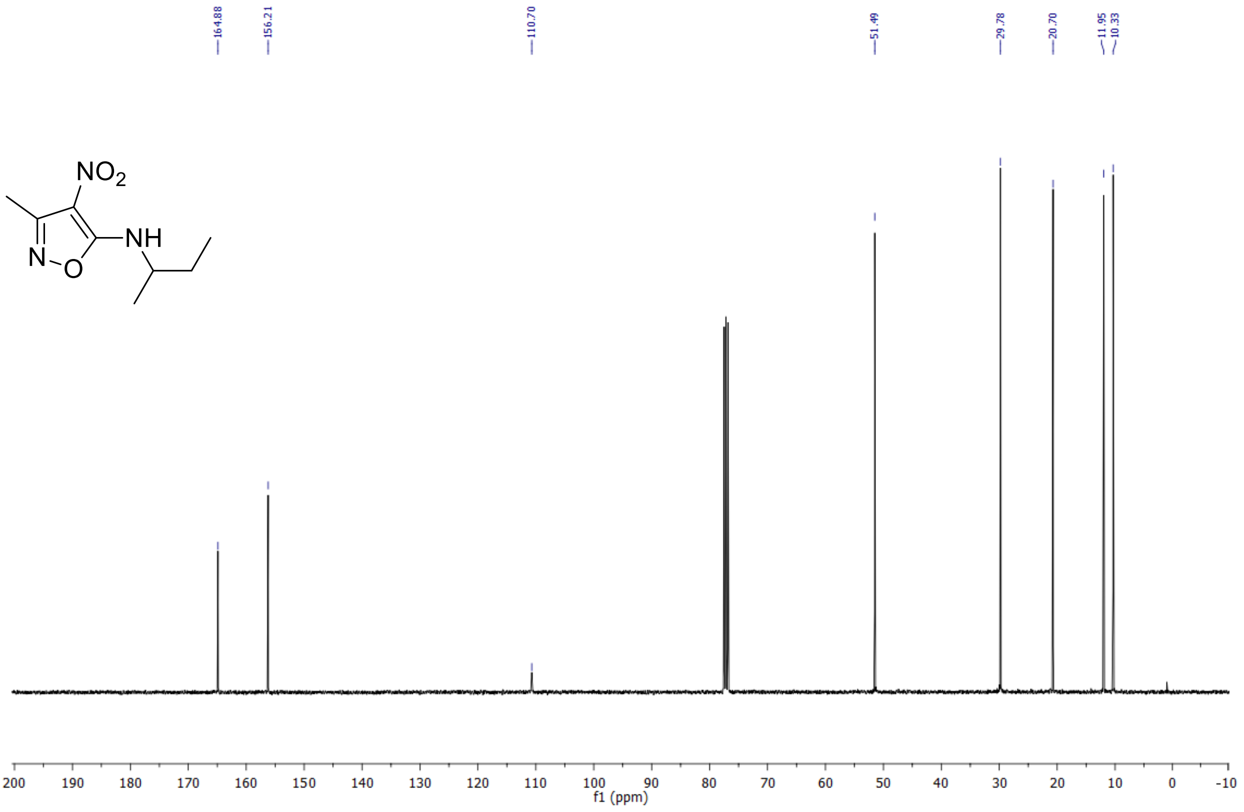

8j ^{1}H-NMR (400 MHz, $CDCl_3$)

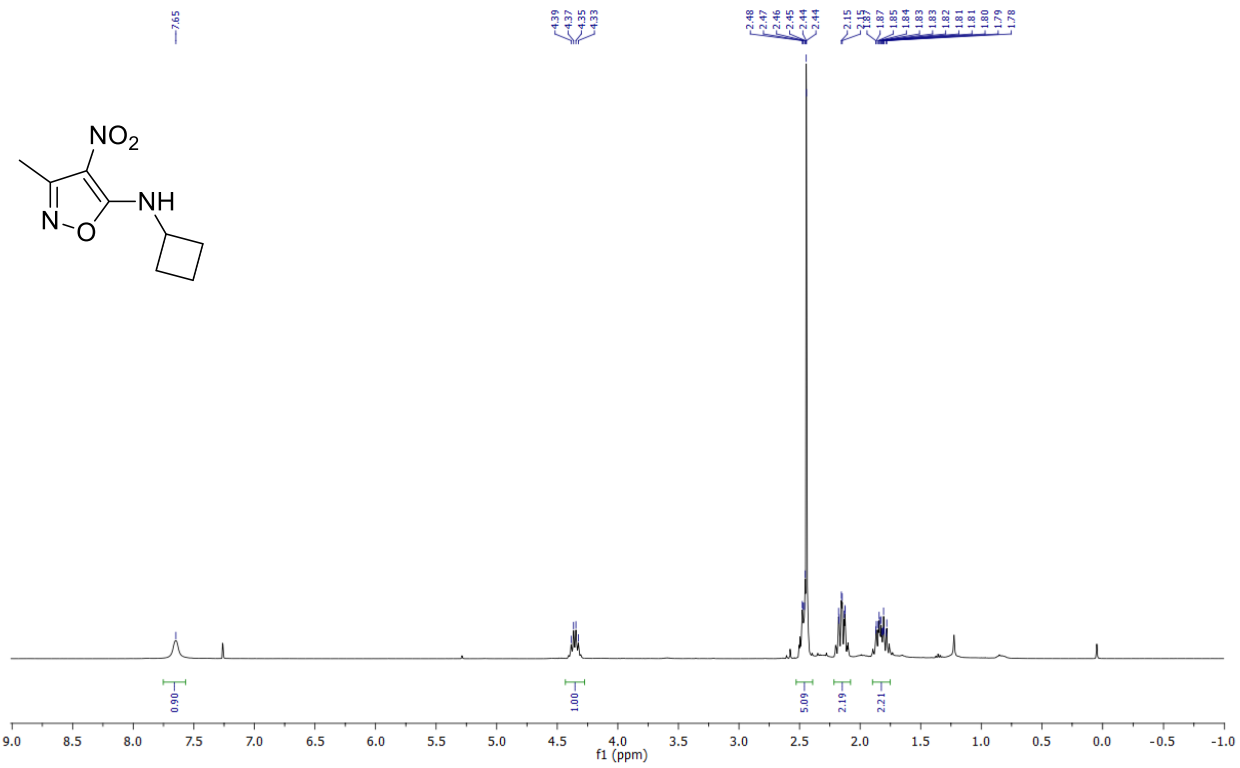

8j ^{13}C-NMR (101 MHz, $CDCl_3$)

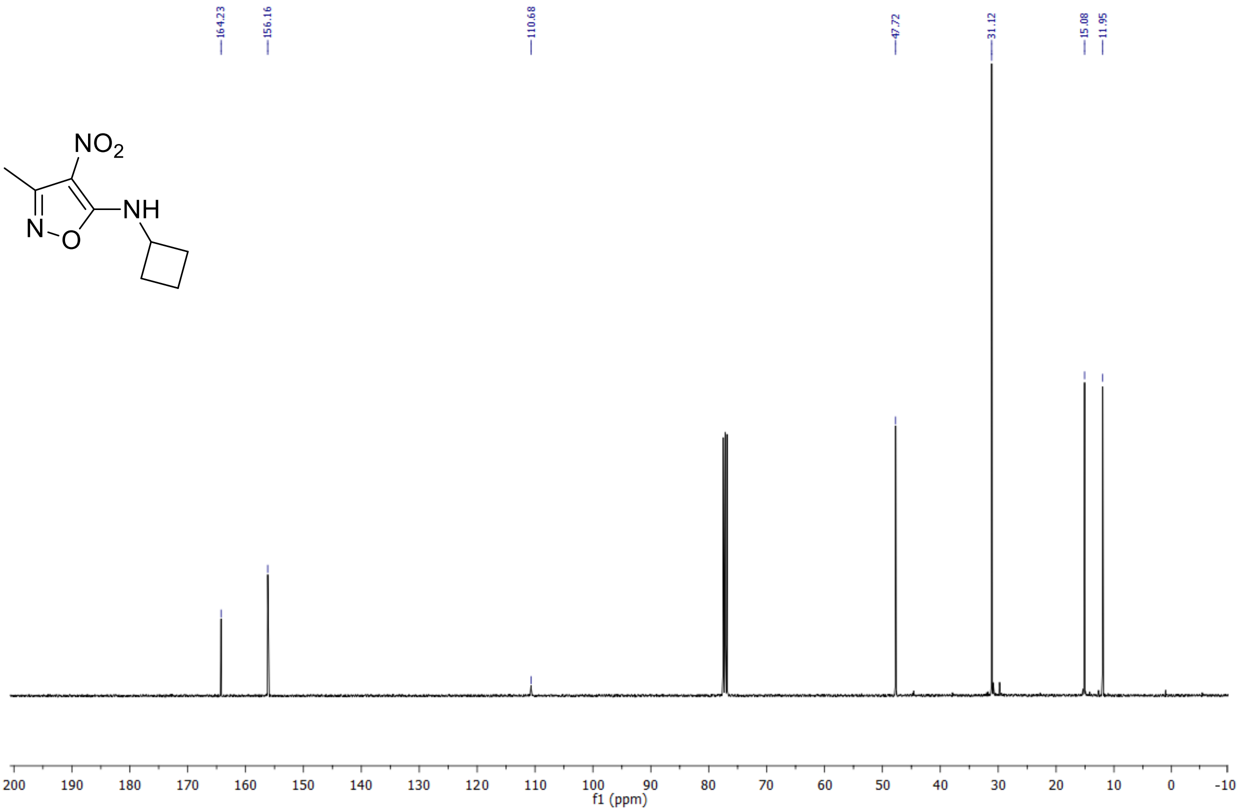

8k ^{1}H-NMR (400 MHz, $CDCl_3$)

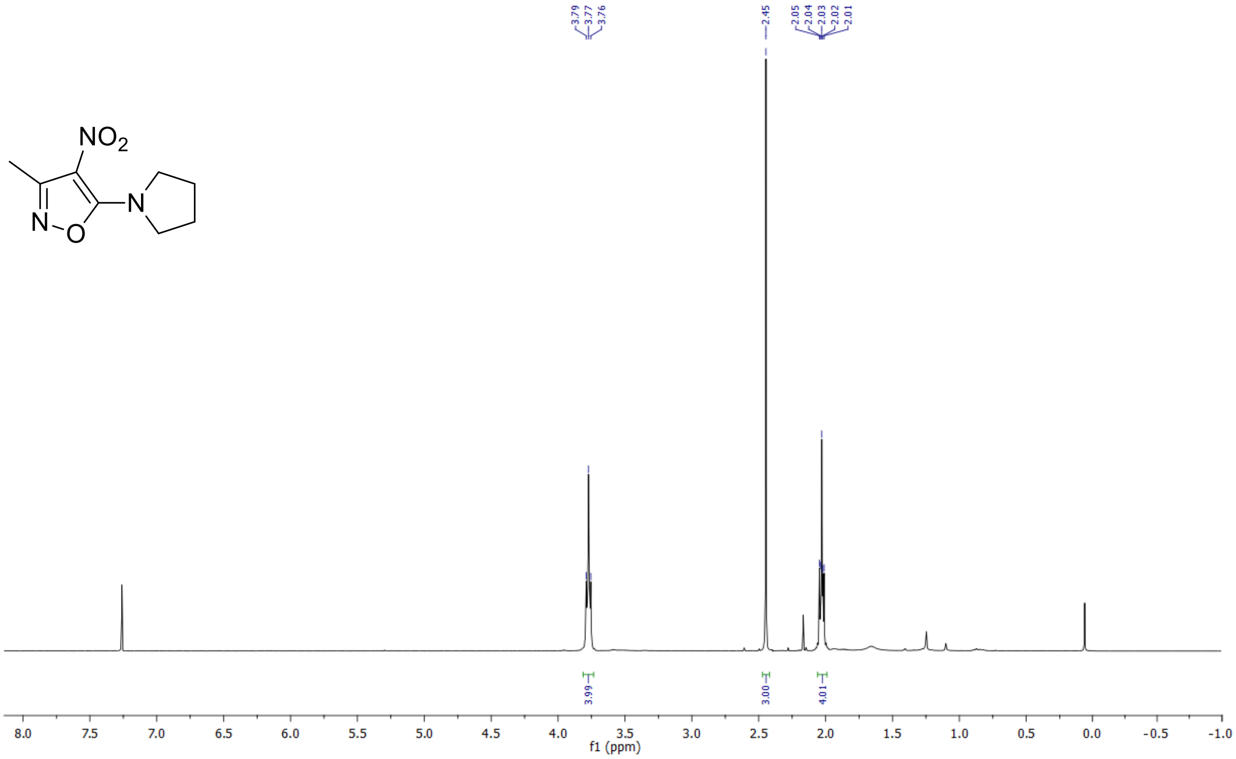

8k ^{13}C-NMR (101 MHz, $CDCl_3$)

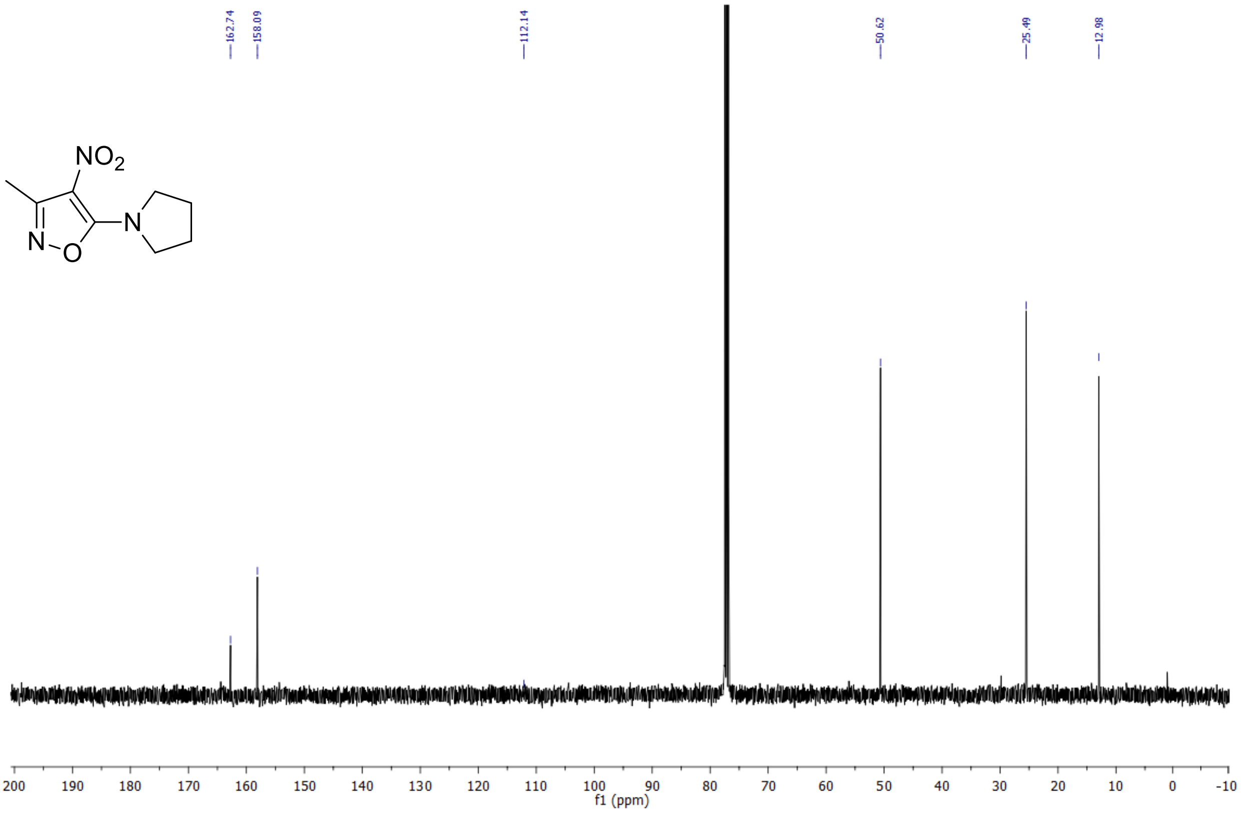

8l ^{1}H-NMR (400 MHz, $CDCl_3$)

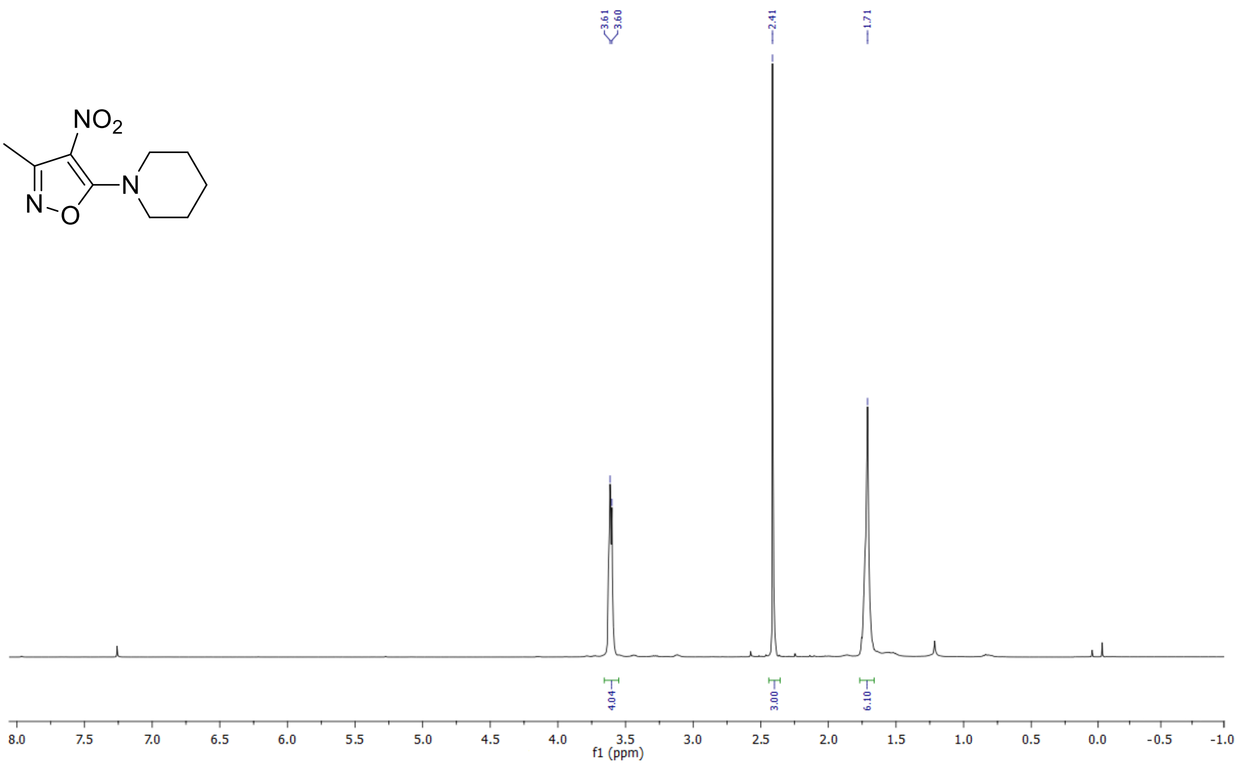

8l ^{13}C-NMR (101 MHz, $CDCl_3$)

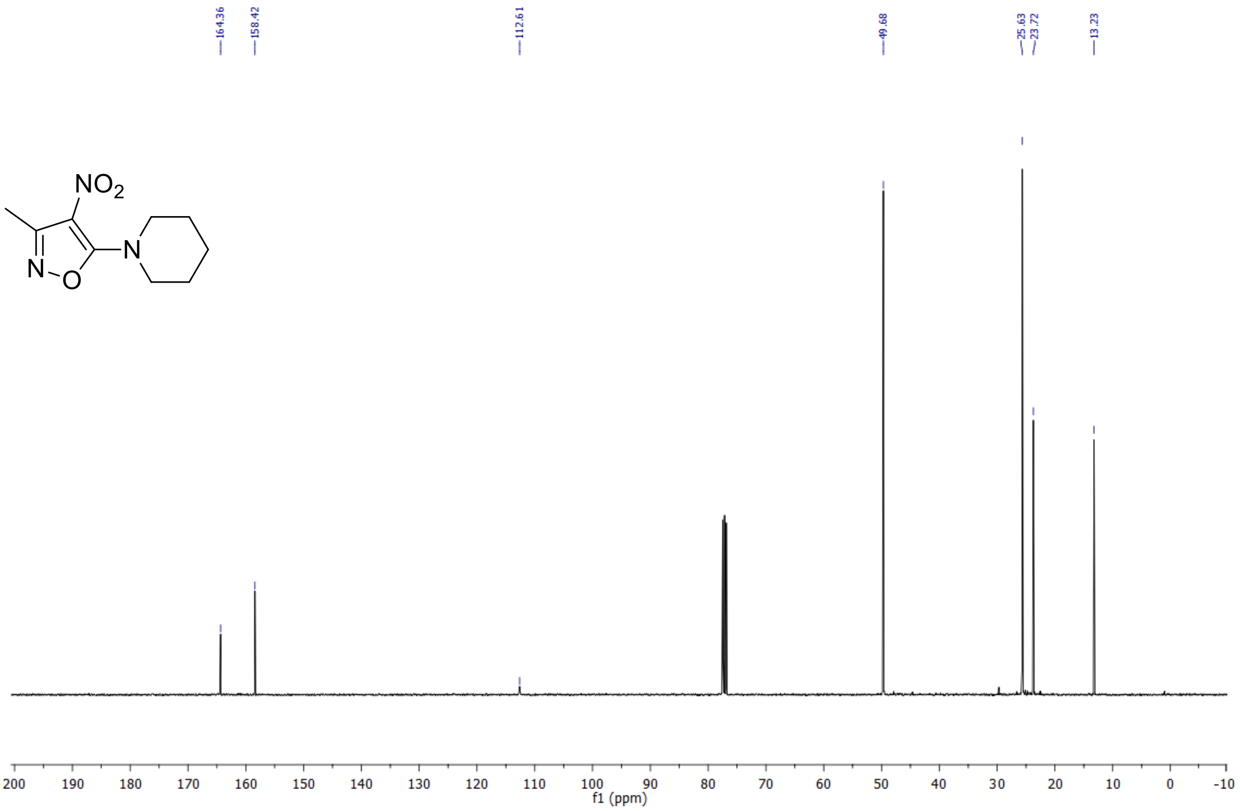

8m ^{1}H-NMR (400 MHz, $CDCl_3$)

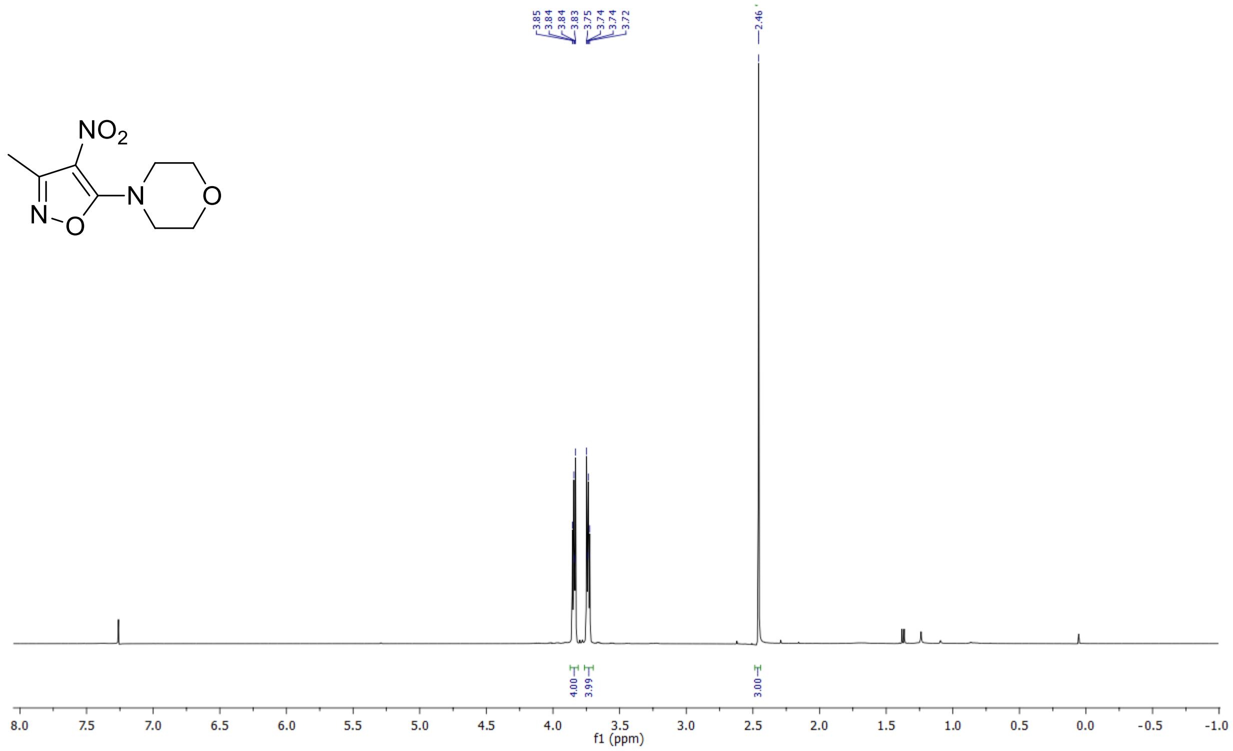

8m ^{13}C-NMR (101 MHz, $CDCl_3$)

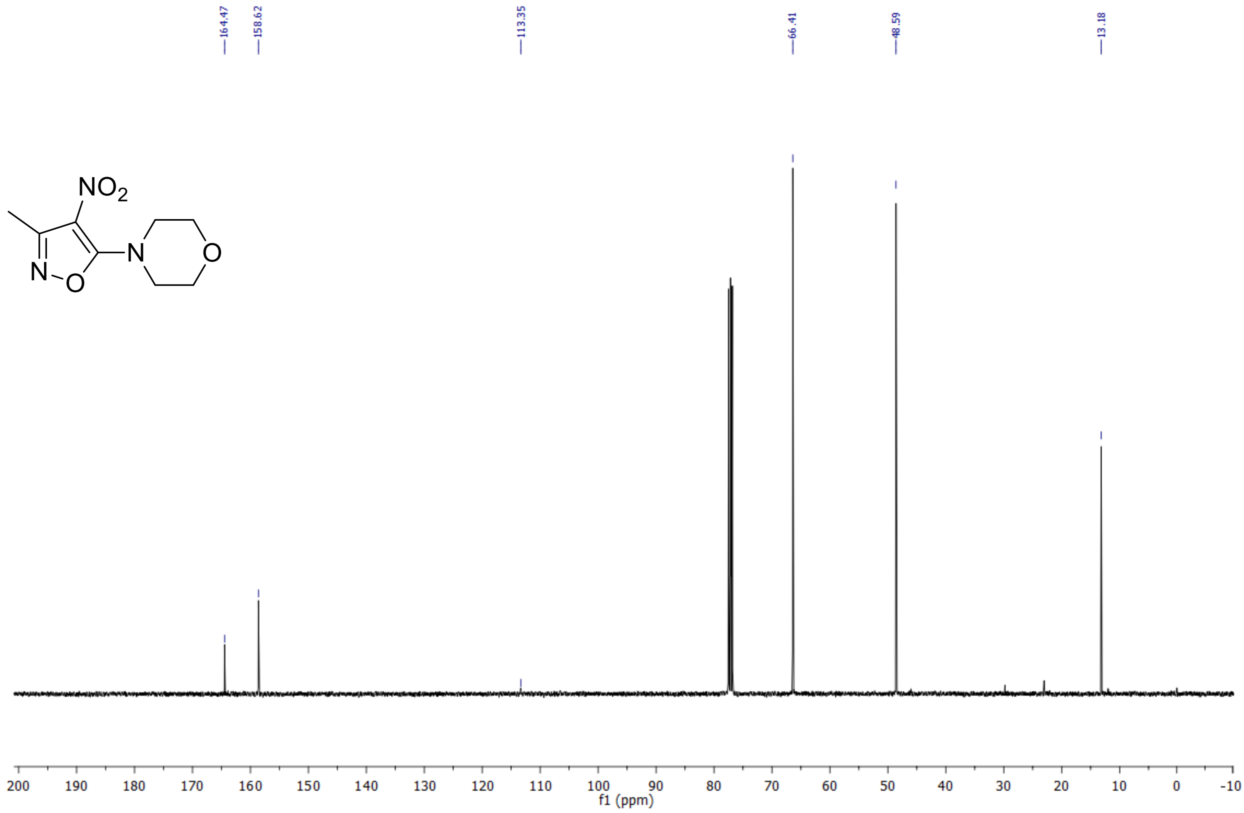

8n ^{1}H-NMR (400 MHz, $CDCl_3$)

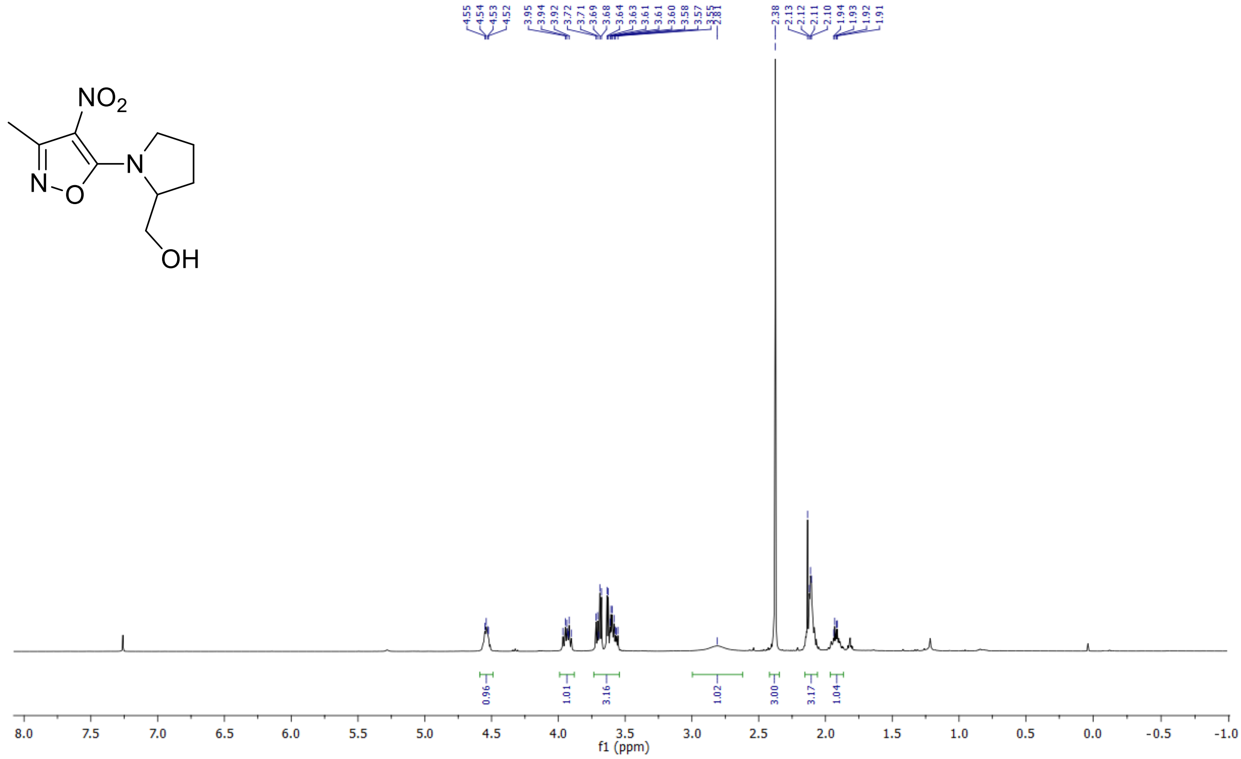

8n ^{13}C-NMR (101 MHz, $CDCl_3$)

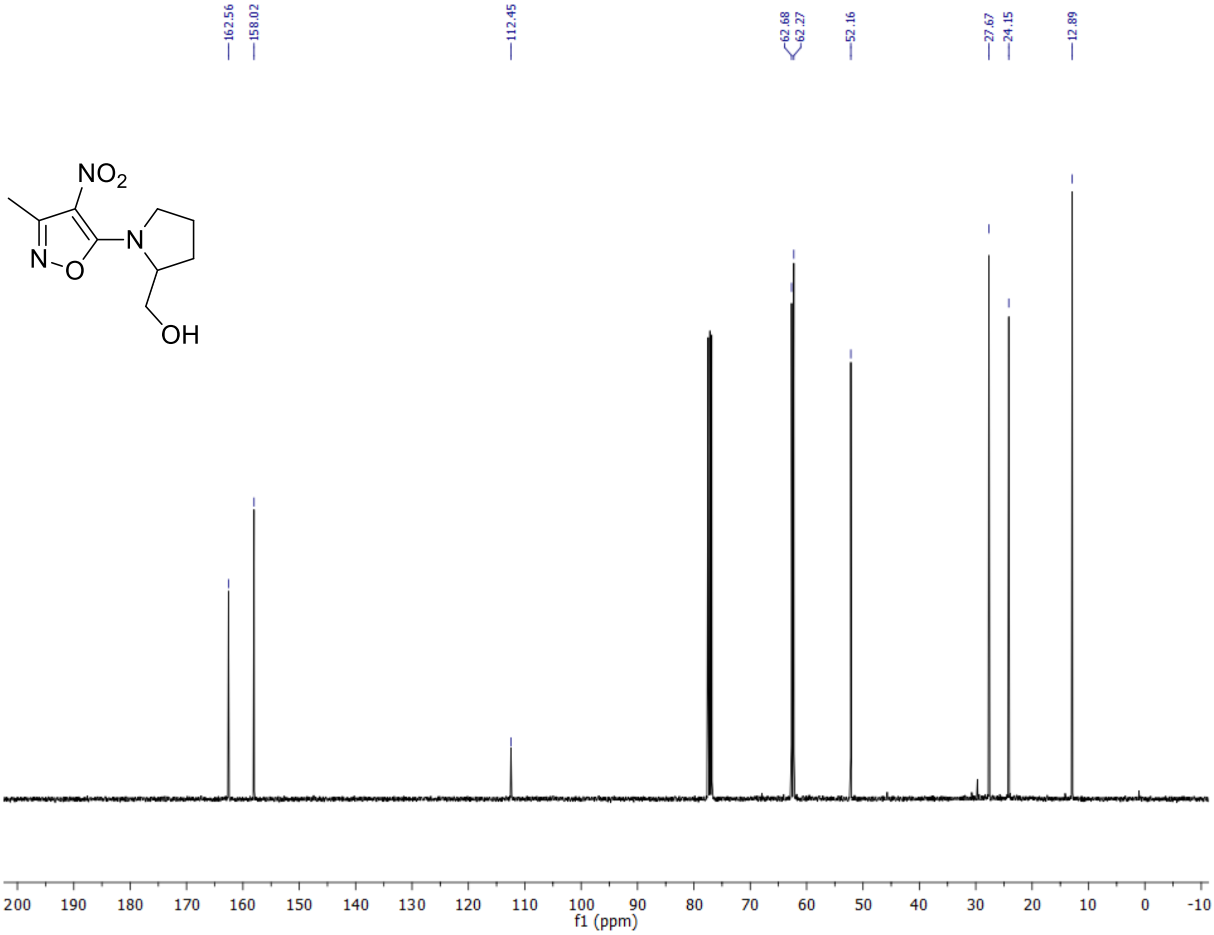

8o ^{1}H-NMR (400 MHz, $CDCl_3$)

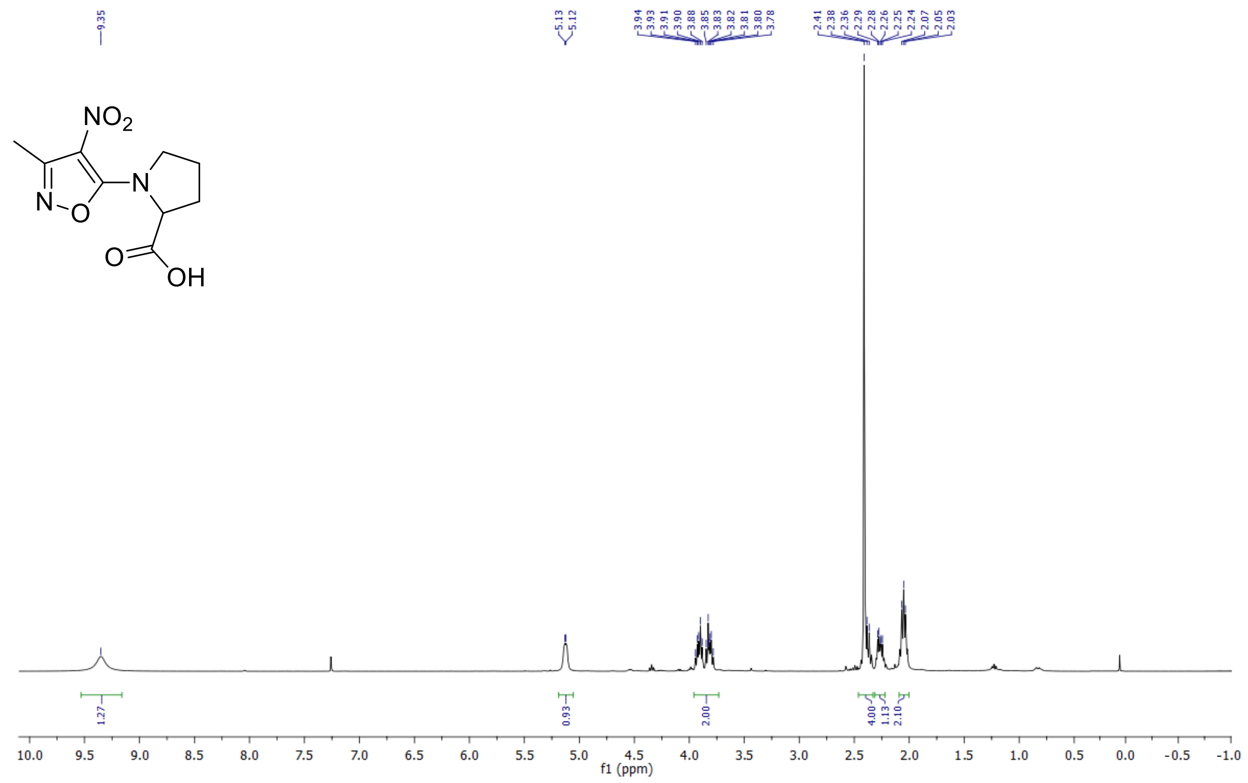

8o ^{13}C-NMR (101 MHz, $CDCl_3$)

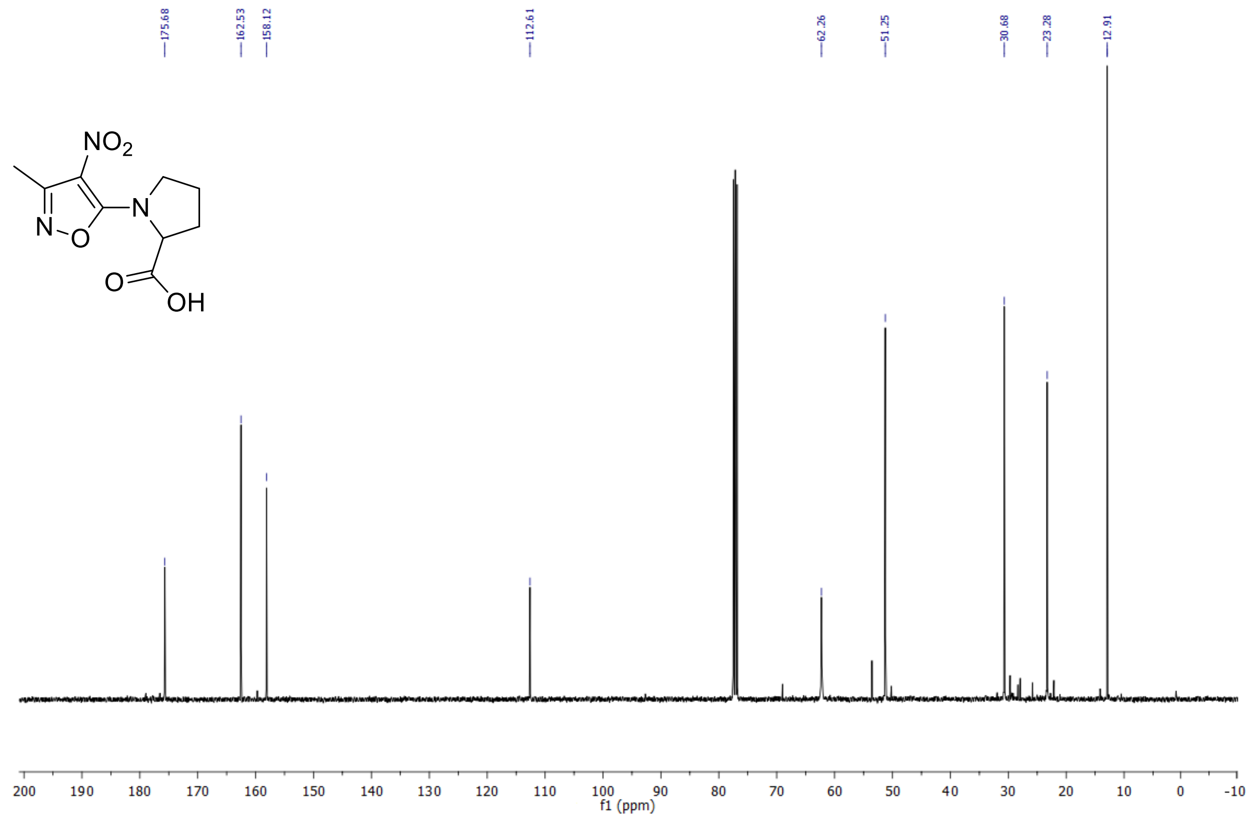

8p ^{1}H-NMR (400 MHz, $CDCl_3$)

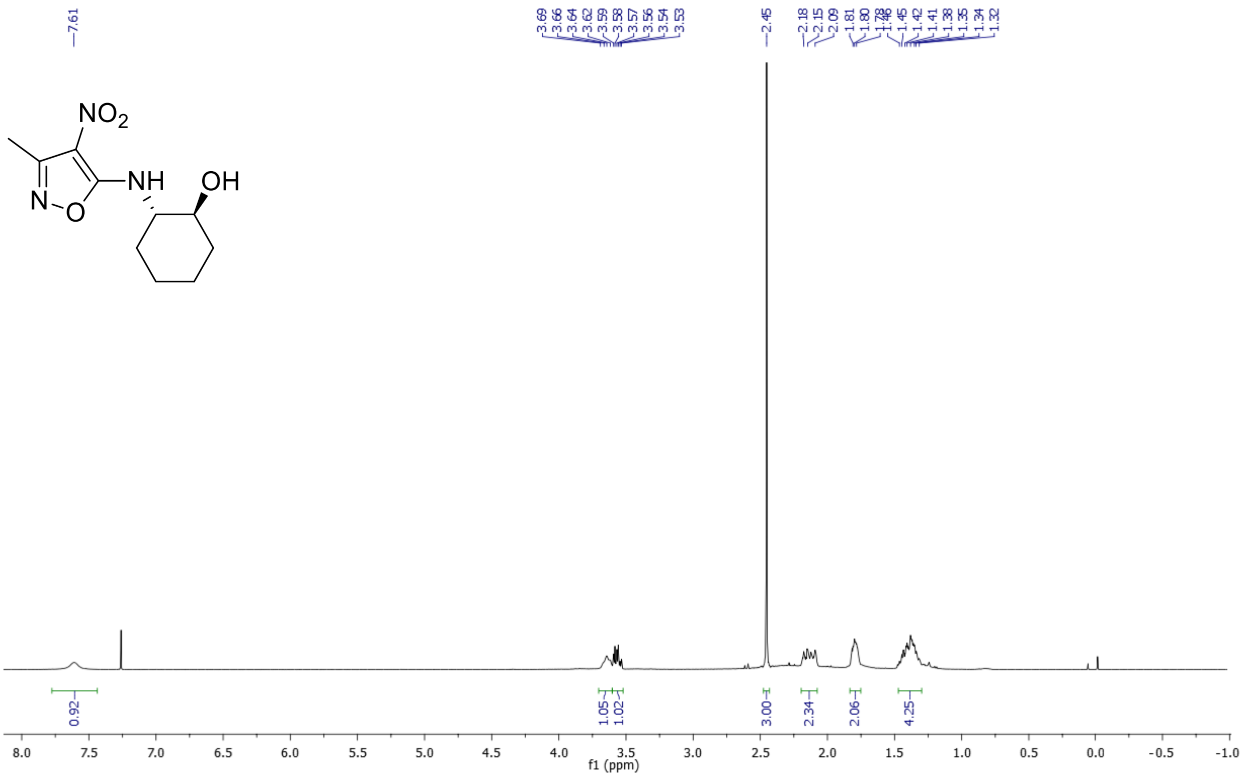

8p ^{13}C-NMR (101 MHz, $CDCl_3$)

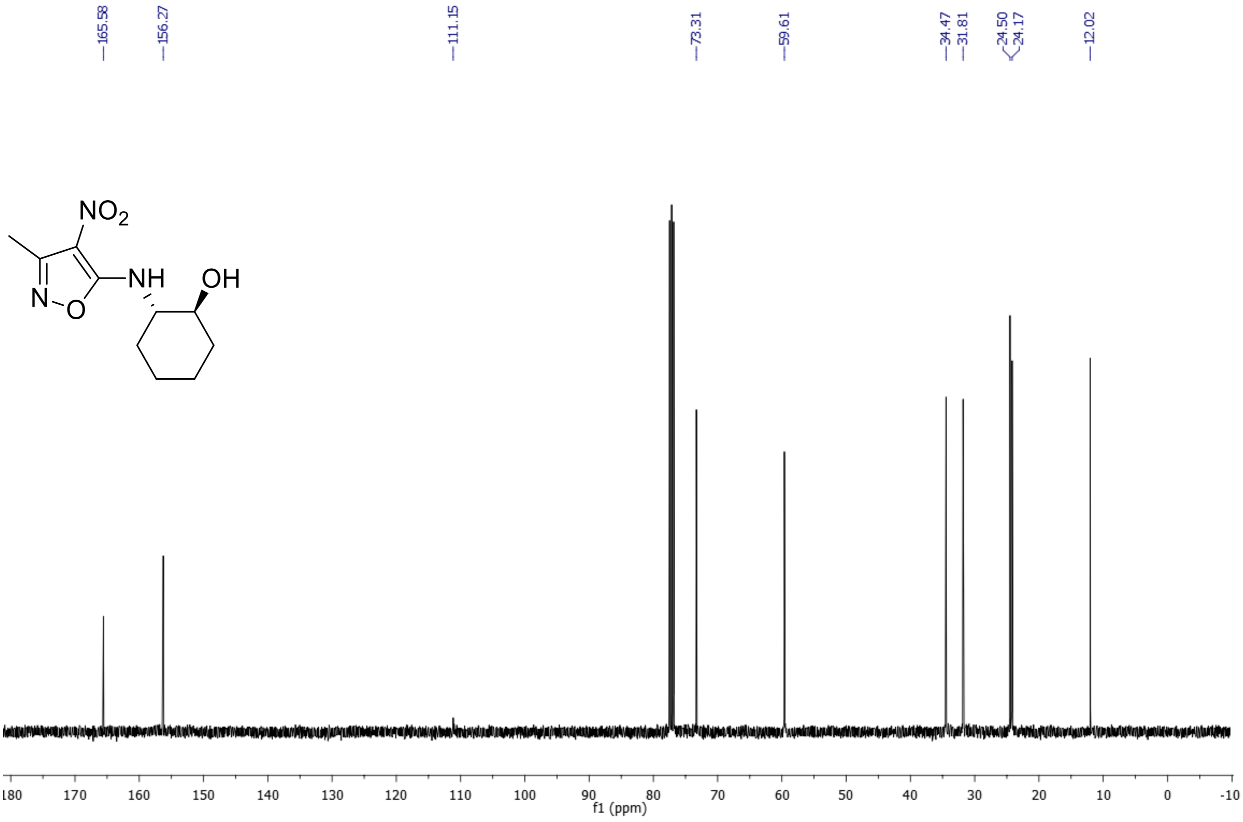

8q ^{1}H-NMR (400 MHz, $CDCl_3$)

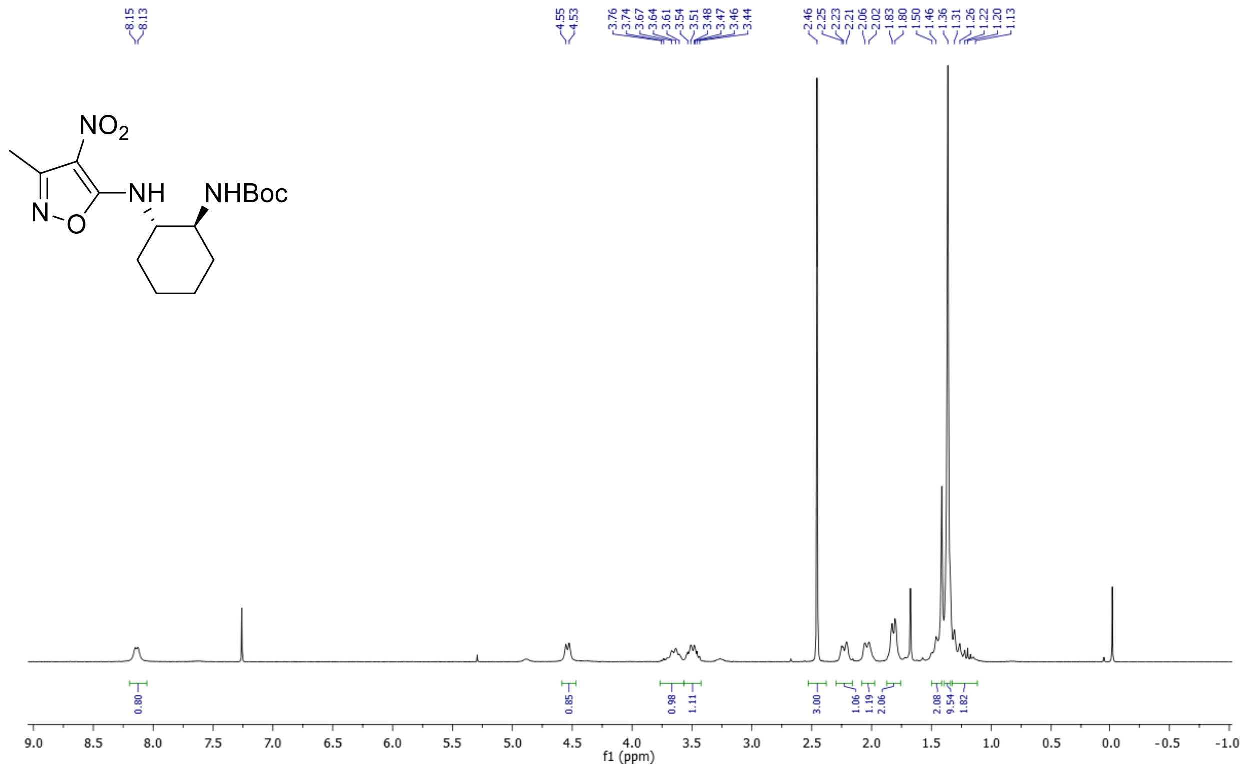

8q ^{13}C-NMR (101 MHz, $CDCl_3$)

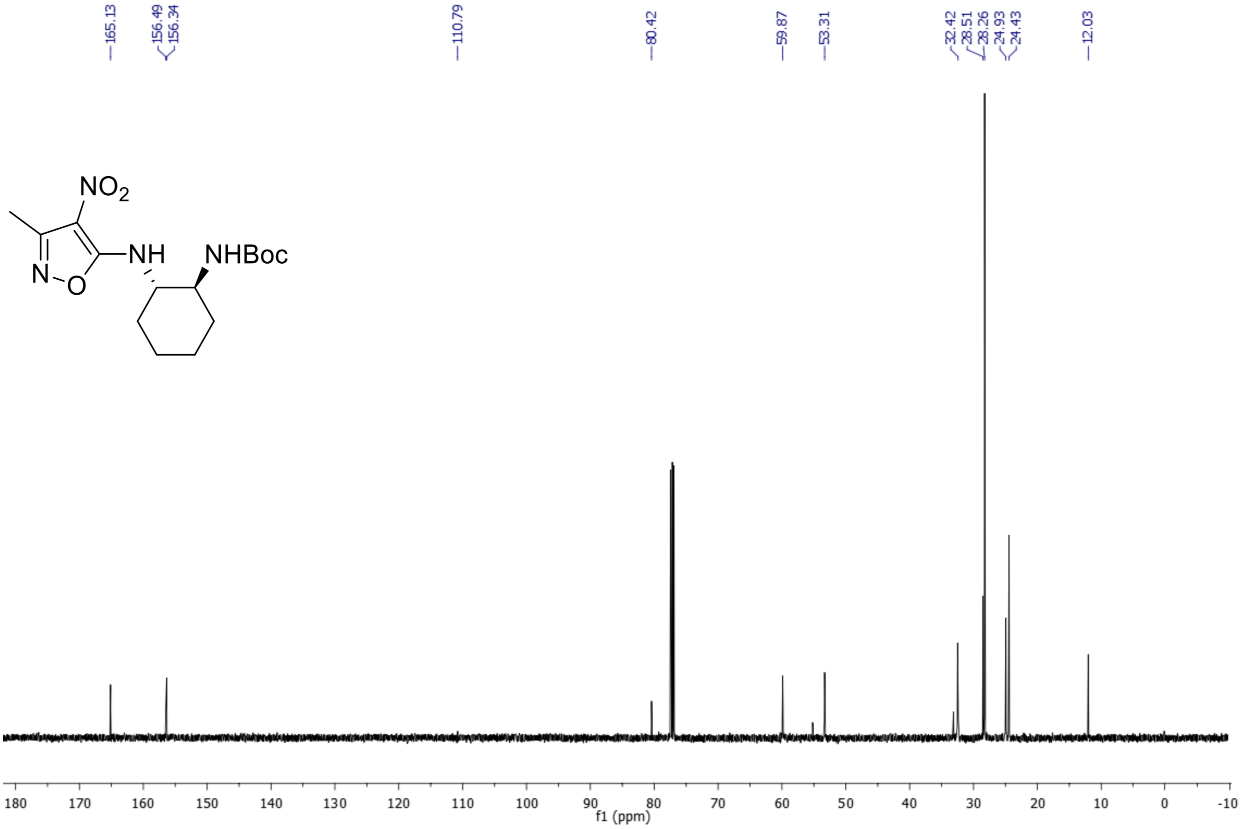

8r ^{1}H-NMR (400 MHz, $CDCl_3$)

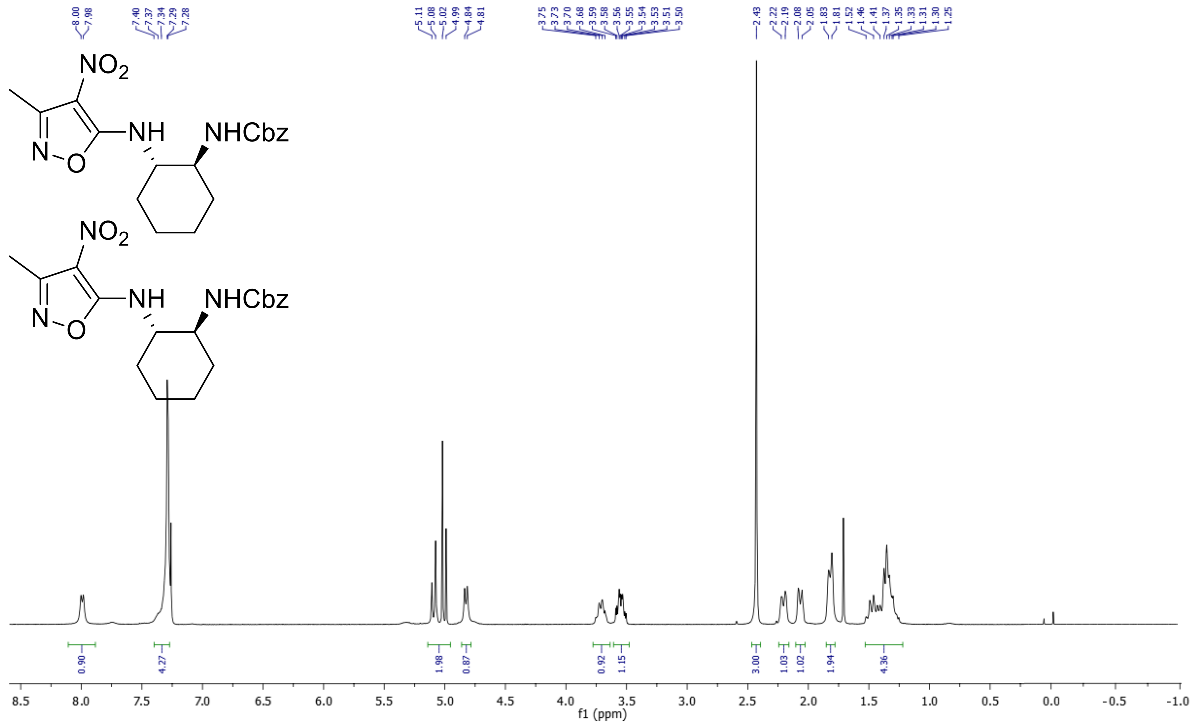

8r ^{13}C-NMR (101 MHz, $CDCl_3$)

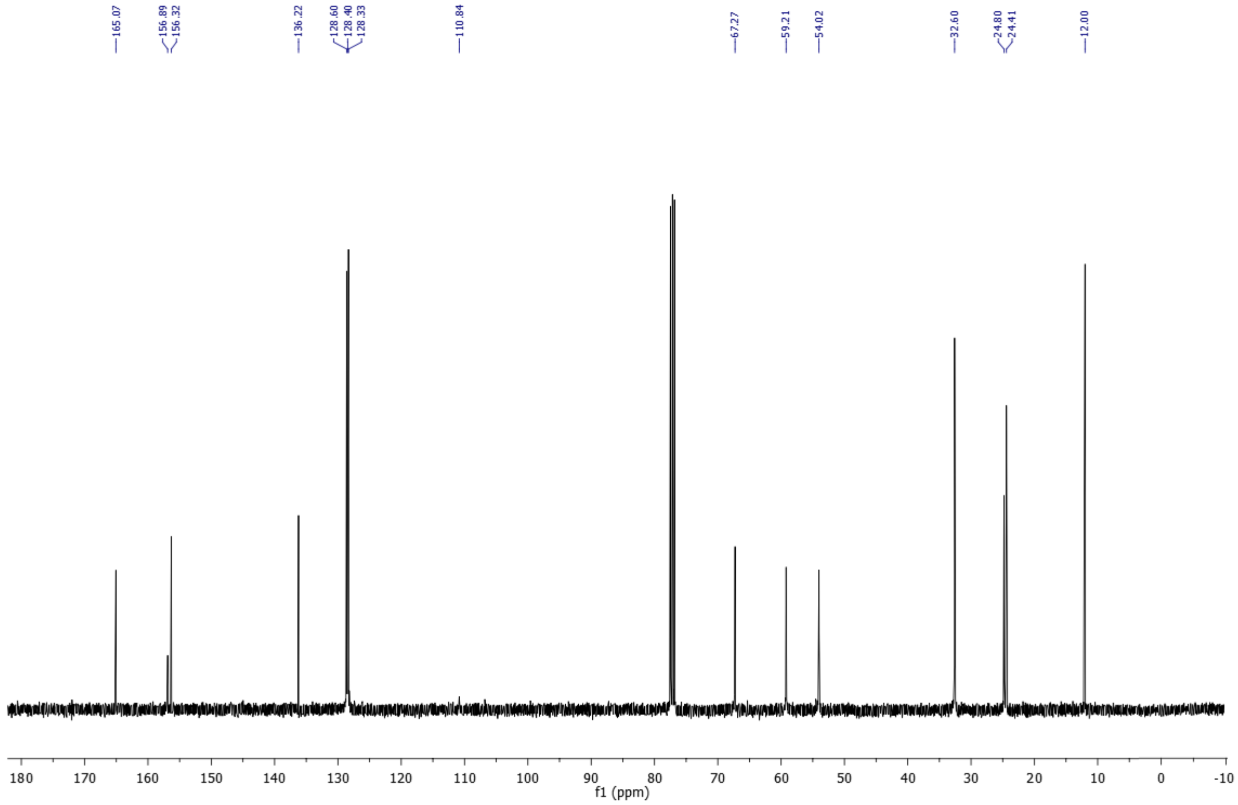

8s ^{1}H-NMR (400 MHz, $CDCl_3$)

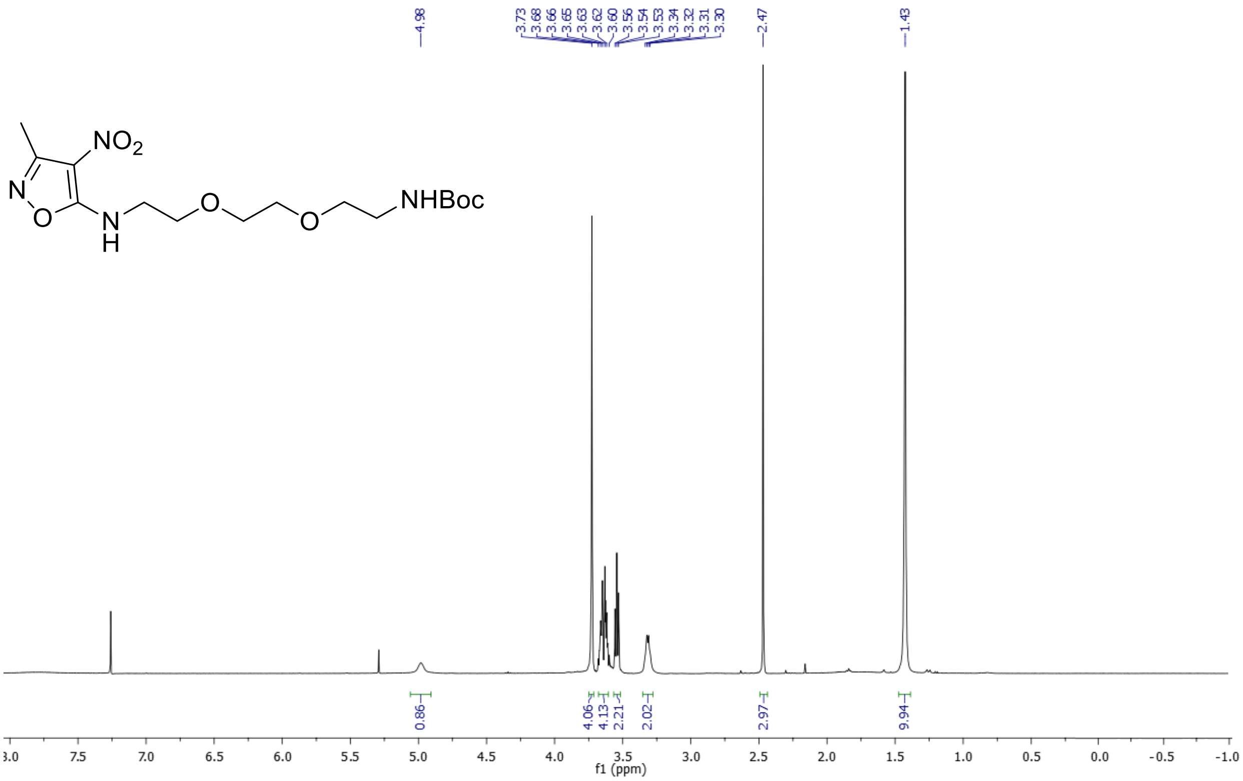

8s ^{13}C-NMR (101 MHz, $CDCl_3$)

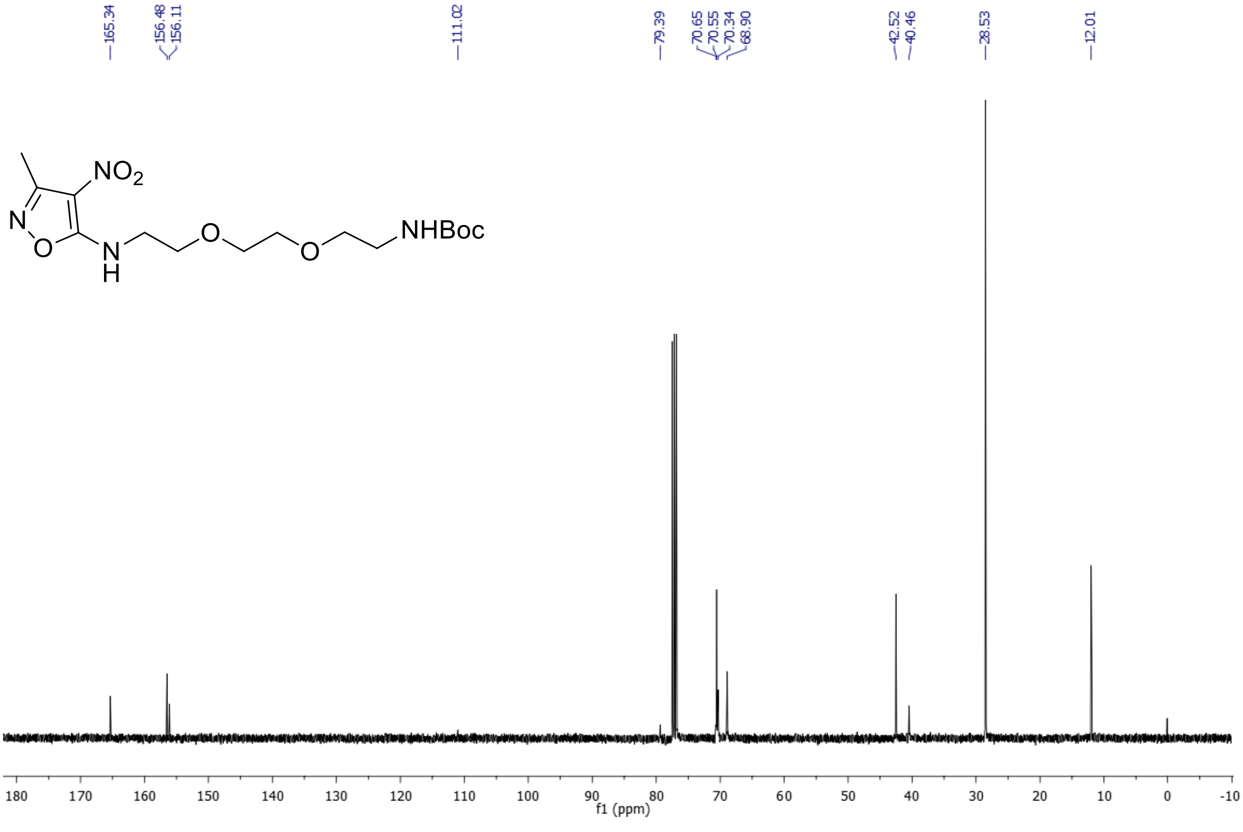

4q ^{1}H-NMR (400 MHz, $CDCl_3$)

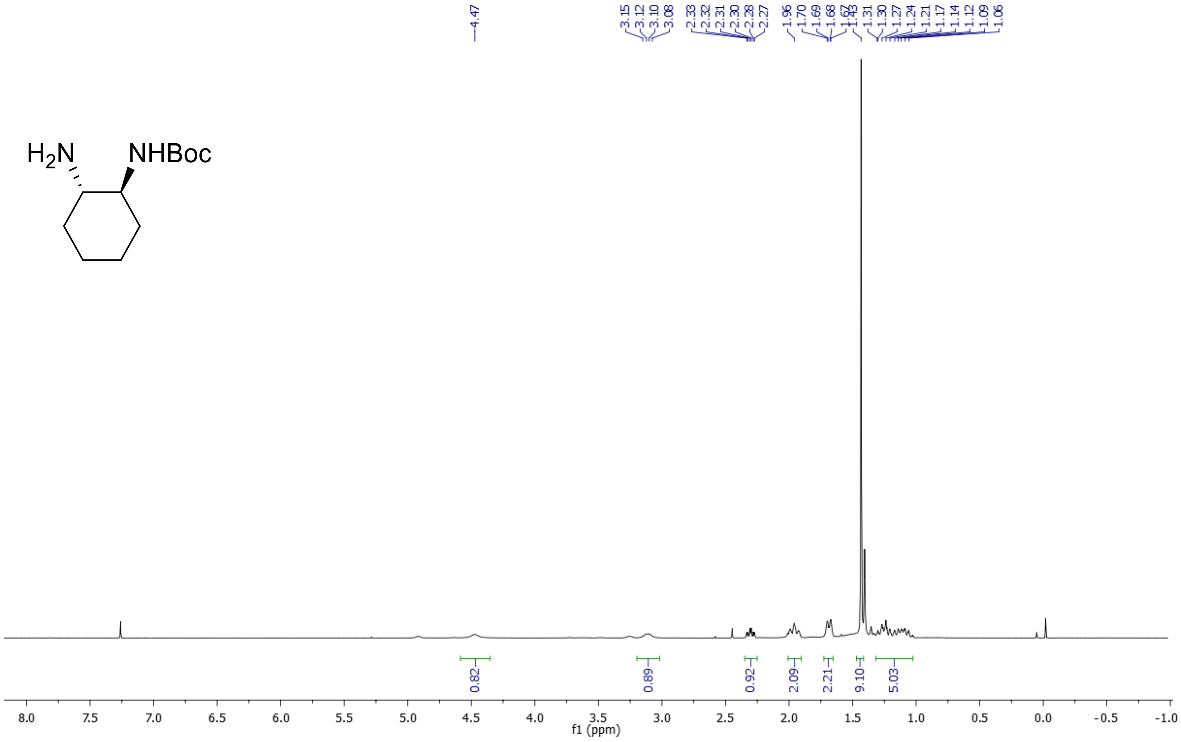

4q ^{13}C-NMR (101 MHz, $CDCl_3$)

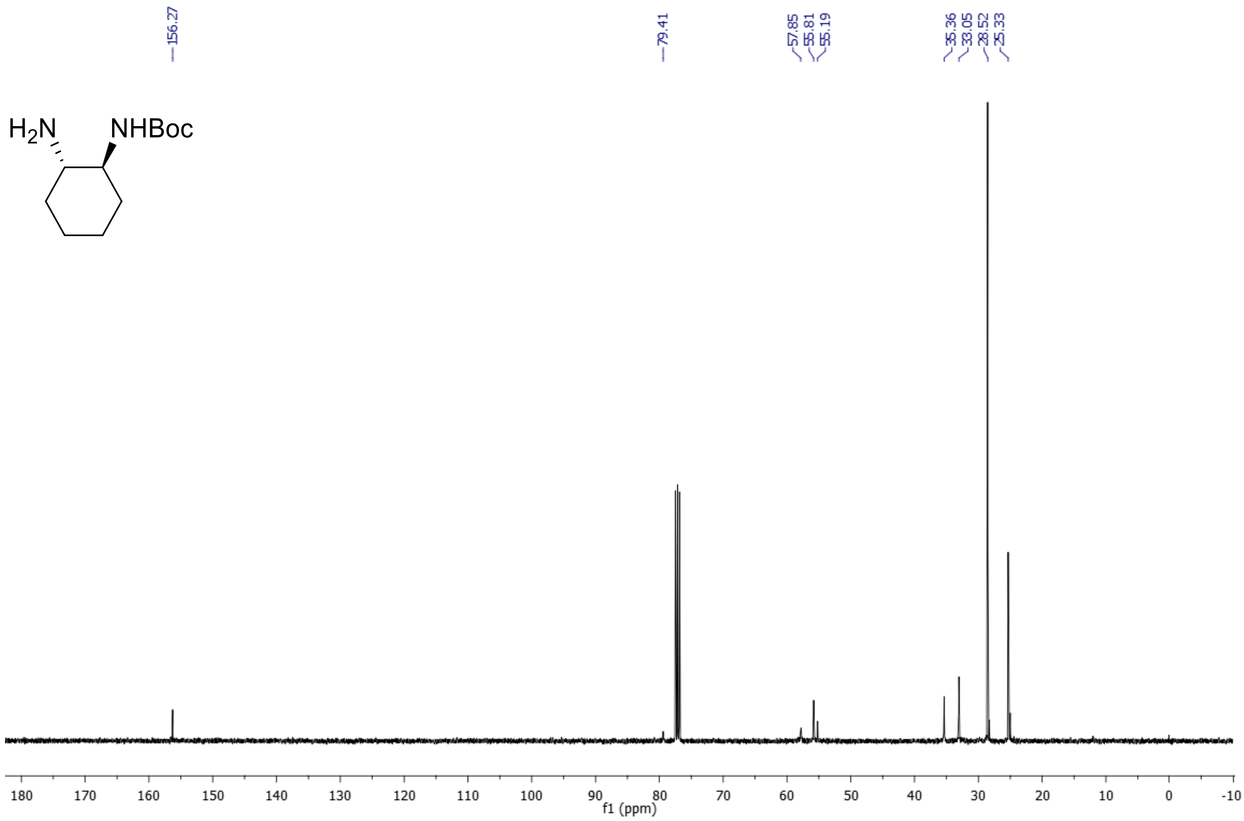

16 ^{1}H-NMR (400 MHz, $CDCl_3$)

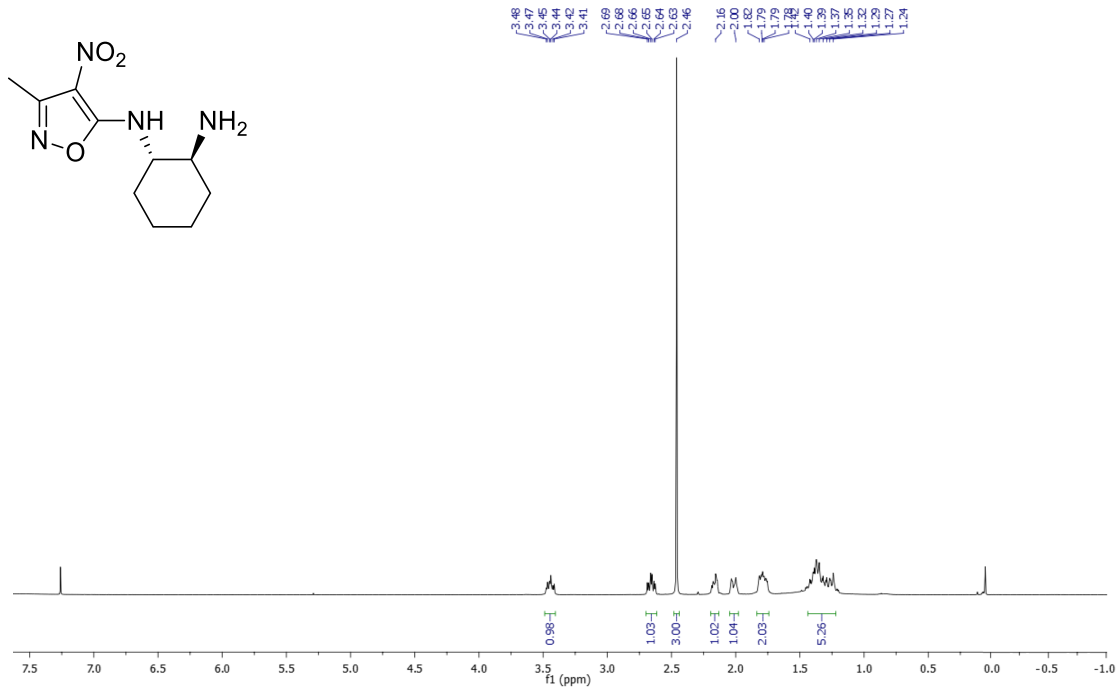

16 ^{13}C-NMR (101 MHz, $CDCl_3$)

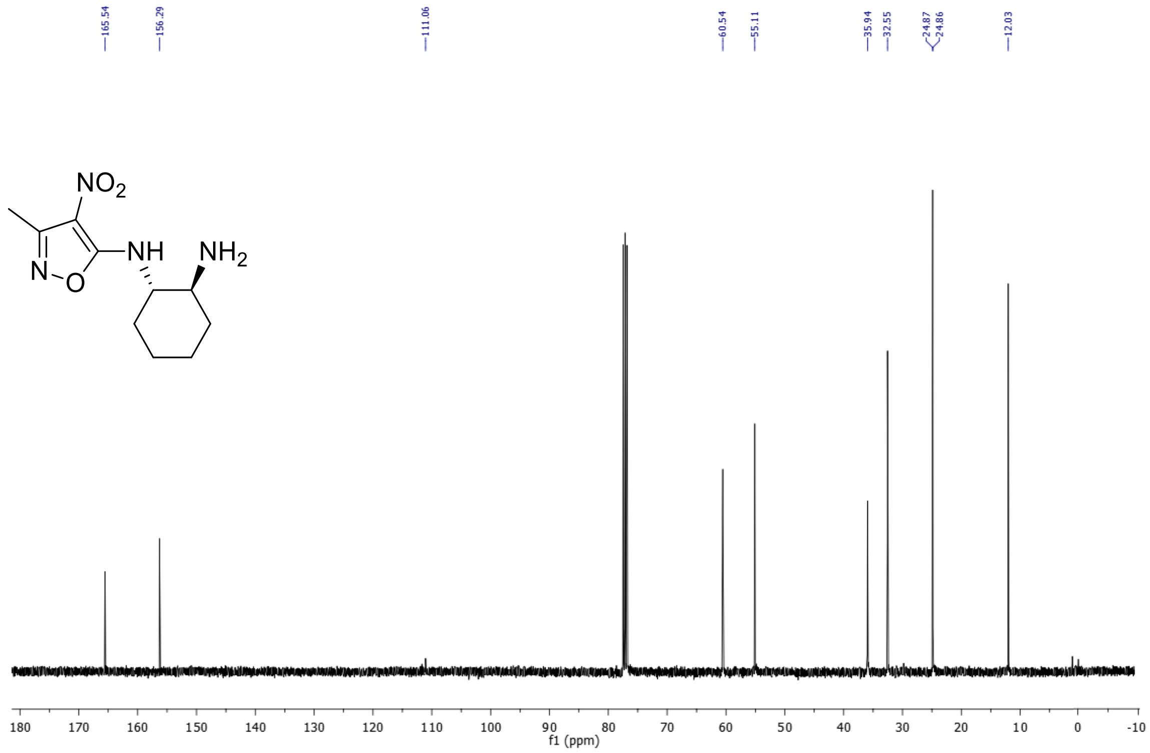

4s ^{1}H-NMR (400 MHz, $CDCl_3$)

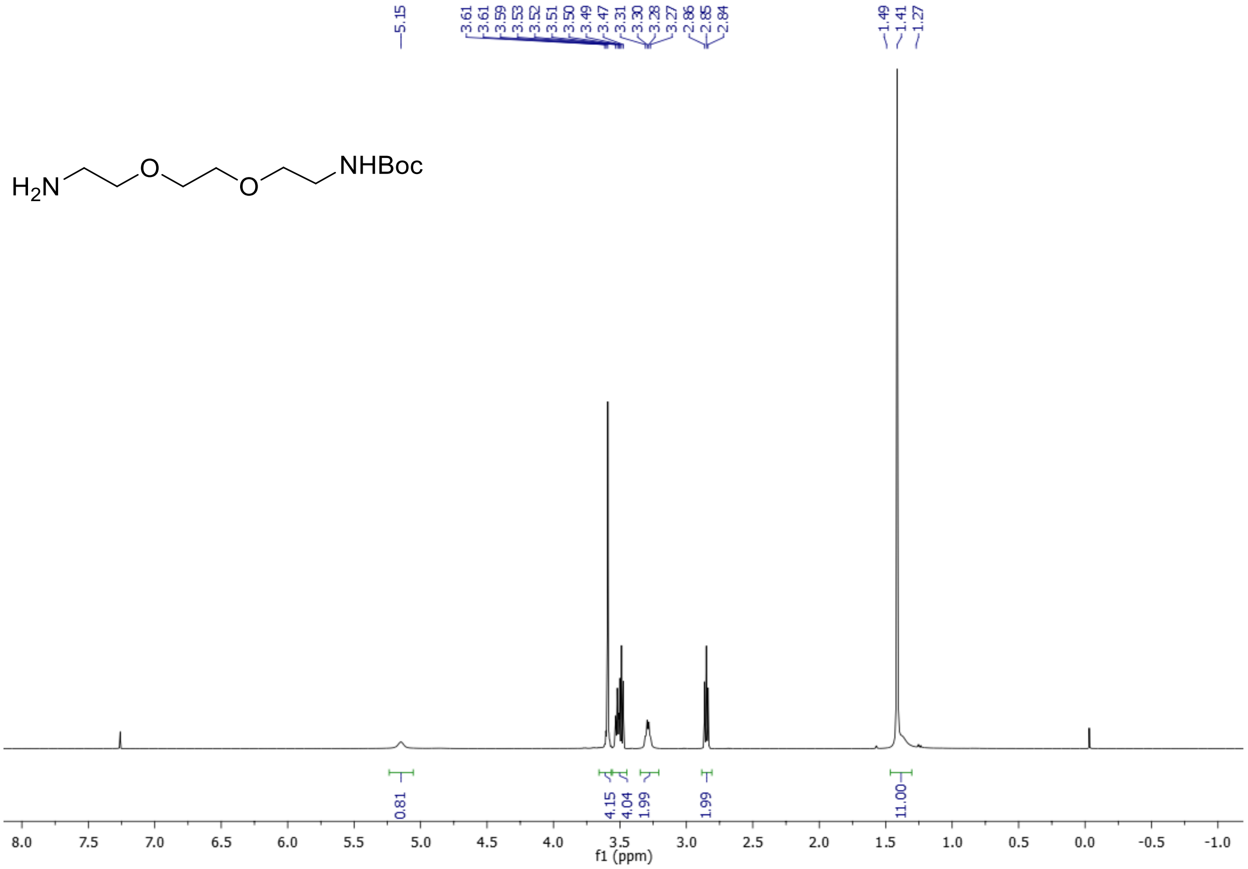

4s ^{13}C-NMR (101 MHz, $CDCl_3$

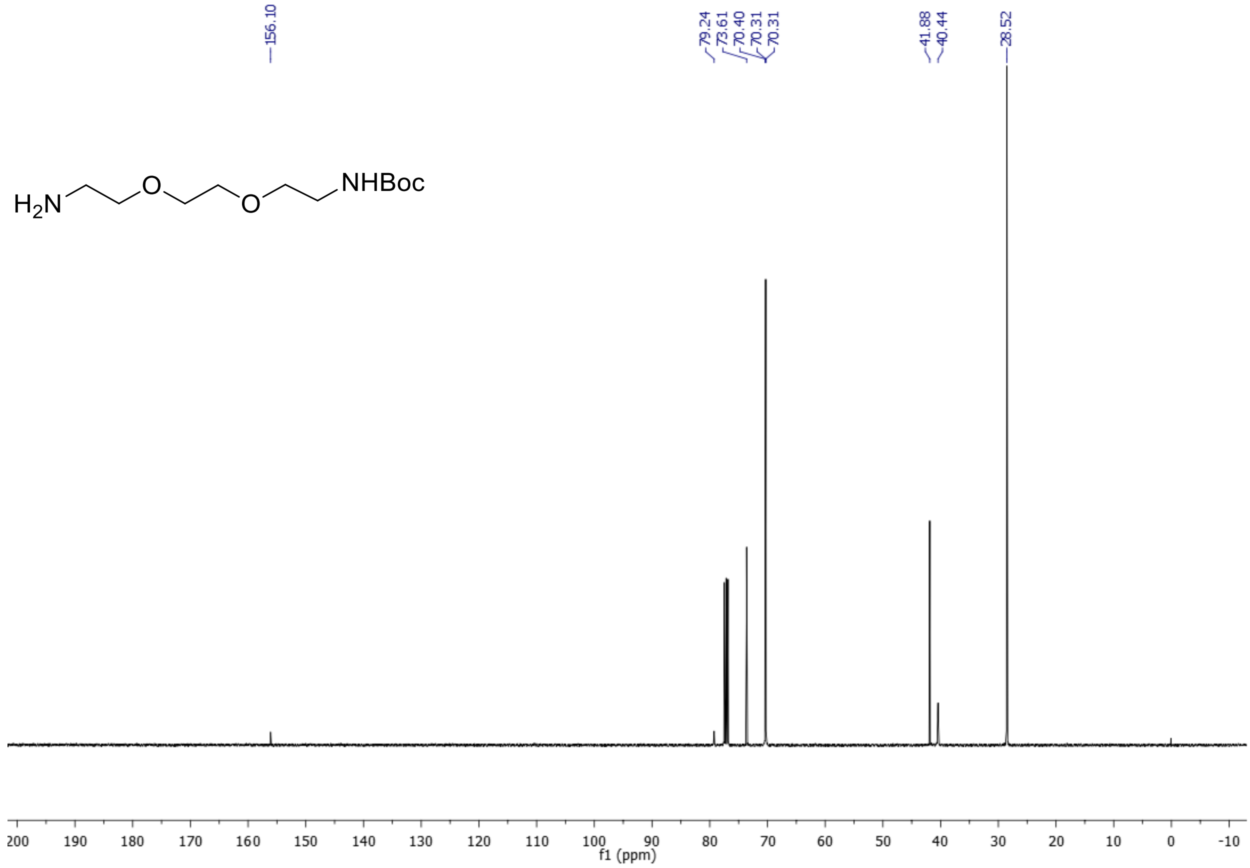

17 ^{1}H-NMR (400 MHz, $CDCl_3$)

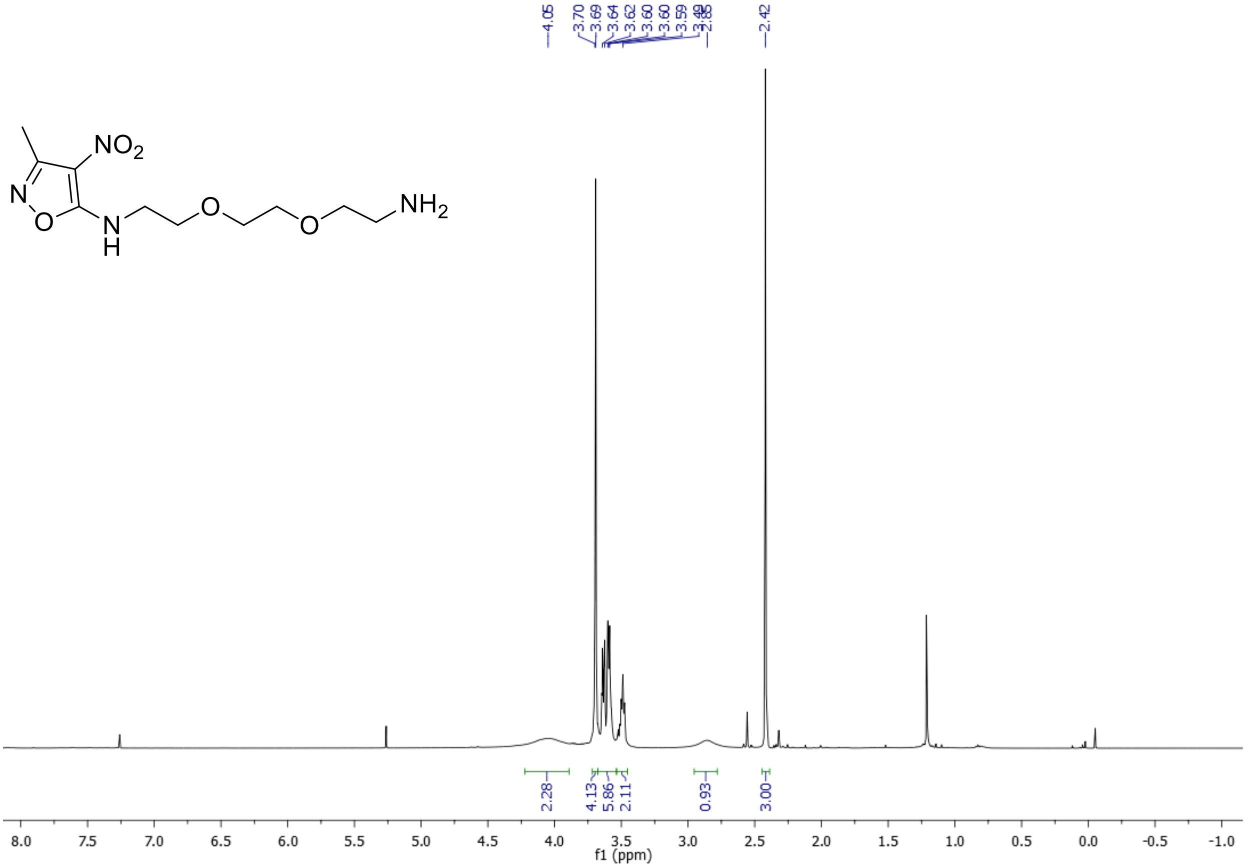

17 ^{13}C-NMR (101 MHz, $CDCl_3$)

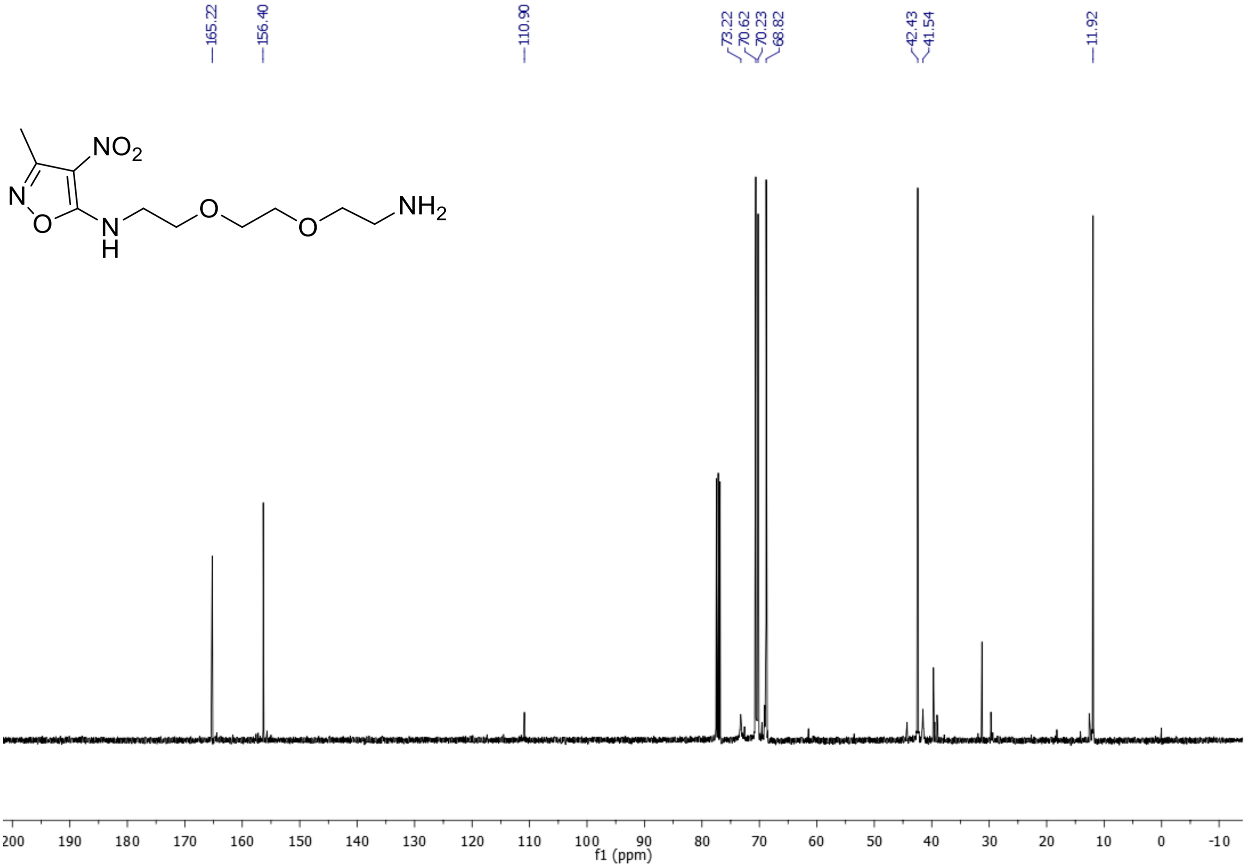

References:

[1] S. C. Virgil, T. V. Hughes, D. Qiu, J. Wang, N-Chlorosuccinimide. *e-EROS Encyclopedia of Reagents for Organic Synthesis*; Wiley, **2012**.

[2] S. C. Virgil, P.R. Jenkins, A. J. Wilson, M. D. G. Romero, N-Bromosuccinimide. *e-EROS Encyclopedia of Reagents for Organic Synthesis*; Wiley, **2006**.

[3] a) K. Błaziak, W. Danikiewicz, M. Mąkosza, *J. Am. Chem. Soc*. **2016**, *138*, 7276–7281; b) J. F. Bunnett, R. E. Zahler, *Chem. Rev.* **1951**, *49*, 273–412.

[4] a) S. Rohrbach, A. J. Smith, J. Hao Pang, D. L. Poole, T. Tuttle, S. Chiba, J. A. Murphy, *Angew. Chem. Int. Ed.* **2019**, *58*, 16368–16388; b) A. J. J. Lennox, *Angew. Chem. Int. Ed.* **2018**, *57*, 14686–14688; c) E. E. Kwan, Y. Zeng, H. A. Besser, E. N. Jacobsen, *Nature Chem.* **2018**, *10*, 917–923.
